# Abstracts of scientific papers and sessions, CPA Congress 2012

**DOI:** 10.3138/physio.64.supp

**Published:** 2012-07

**Purpose/Objectives and Rationale:** The purpose of this study was to search and appraise the current evidence related to the psychometric properties of the Patient-Rated Wrist Evaluation.

**Relevance to Physiotherapy Practice:** The Patient-Rated Wrist Evaluation is a commonly used outcome measure for assessing pain and functions in patients with different wrist/hand injuries. A systematic review of the psychometric properties of this outcome can provide useful information to physiotherapists for making evidence-informed decisions while using it in their practice.

**Materials and Methods:** Medline, PubMed, CINAHL and Embase databases were searched using pre-determined search terms. Studies were selected for the review based on certain inclusion/exclusion criteria. Two raters independently followed standardized guidelines for data extraction and appraisal of the included studies.

**Analysis:** Standardized evaluation tool was used for performing critical appraisal and quality rating of the included studies. Descriptive summary of the psychometric properties of the patient-rated wrist evaluation was prepared.

**Results:** Fifteen articles were included in the review. Quality check determined that 8 studies had quality level of greater than 70%. Construct validity of the patient-rated wrist evaluation was commonly assessed with other similar outcomes such as the Disabilities of the Arm, Shoulder, and Hand questionnaire with Spearman’s correlation coefficient in range of 0.62–0.82 (*p* < 0.001). Test-retest reliability was determined on using Intraclass Correlation Coefficient which ranged between 0.78–0.94. Responsiveness was shown with Standardized Response Mean ranging from 1.51–2.27.

**Conclusions:** The review determined that the Patient-Rated Wrist Evaluation has adequate psychometric properties while using in patients with wrist/hand injuries. The translated versions of the outcome also met or exceeded the benchmarks for acceptable psychometric properties.

**Keywords:** outcome measurement, systematic review, wrist pain and disability, psychometric properties
