# Supplementary material for: Abstracts of scientific papers and sessions, CPA Congress 2012
Source: Physiother Can. 2012 Jul;64(Suppl 1):1–66. doi: 10.3138/physio.64.supp (PMC4098077; doi:10.3138/physio.64.supp)
Supplement: Related texts

# Abstract: A010 – PHYSIOLOGICAL CORRELATES OF HIGH-LEVEL FUNCTIONAL PERFORMANCE IN COPD

**Published:** 2012-05

**Authors:** SJ Butcher, BJ Pikaluk, NM Heynen, RL Chura, JP Farthing, DD Marciniuk

**Affiliations:** University of Saskatchewan, Sask

**Purpose/Objectives and Rationale:** To determine the relationship between functional performance, leg muscle strength/power, aerobic power, and anaerobic power.

**Relevance to Physiotherapy Practice:** Performance of functional activities that require a high level of whole body energy utilization are typically not performed by patients with COPD. Therefore, these patients have reduced functional performance; however, the physiological parameters that contribute to this reduction are unknown.

**Materials and Methods:** 13 patients with COPD (FEV_1_ 46 ± 19% predicted) completed pulmonary function testing, a cardiopulmonary exercise test, quadriceps dynamometry (isometric peak torque (IsoPT) and rate of torque development (IsoRTD), concentric peak torque, and eccentric peak torque at 90° s-1 (EccPT)), a steep ramp (SRAT) anaerobic cycle ergometry test (increments of 25W 10s-1), and three functional measures (timed up and go (TUG), stair climb (SC), and 30 second sit to stand (STS)).

**Analysis:** Pearson’s r correlation coefficients and multiple regression were calculated.

**Results:** TUG time correlated significantly (*p* < 0.05) with the SRAT power output (*r* = -0.74), IsoPT (*r* = -0.92), and EccPT (*r* = -0.76). Multiple regression (*R*^2^ = 0.77) revealed IsoPT as the most significant predictor of TUG time. SC time correlated significantly with the SRAT power output (*r* = -0.92) and IsoRTD (*r* = -0.79). Multiple regression (*R*^2^ = 0.89) revealed only SRAT power output as a significant predictor. STS time correlated only with the SRAT power output (*r* = -0.72).

**Conclusions:** Performance on the SRAT test is reflected in all three functional tests. Enhancement of anaerobic power and leg muscle strength may potentially increase the ability of patients with COPD to perform higher level functional tasks.

**Keywords:** COPD, exercise testing, muscle, functional performance, energy systems

# Abstract: A012 – CURRENT UTILIZATION OF AEROBIC EXERCISE IN ADULT NEUROLOGICAL REHABILITATION BY CANADIAN PHYSIOTHERAPISTS

**Published:** 2012-05

**Authors:** M MacKay-Lyons, L Doyle

**Affiliations:** School of Physiotherapy, Dalhousie University, Halifax, N.S.

**Purpose/Objectives and Rationale:** Although aerobic exercise (AE) has been shown to improve aerobic capacity and reduce morbidity in neurological populations, its clinical application remains challenging. The purpose of this study was to survey Canadian physiotherapists practising in neuro-rehabilitation to document current practices regarding screening for, and prescription of, AE.

**Relevance to Physiotherapy Practice:** Documenting current patterns of utilization is an important preliminary step in the development of clinical guidelines for best practices in AE for neurological populations.

**Materials and Methods:** Canadian physiotherapists in neurological rehabilitation practice were invited to participate in a 30-item online survey about current utilization of AE.

**Analysis:** Relative frequencies (i.e., number selecting a particular choice on an item/total number of responses to that item x 100) were calculated.

**Results:** AE should be incorporated into treatment programs of patients with neurological conditions.” Although 77% prescribed AE, barriers to use included concerns about cardiac status (11%), lack of equipment (10%), and cognitive/perceptual deficits (9%). Lack of time was identified less frequently (< 2%). For screening, 19% relied on health records and 1% used exercise stress tests. Overground walking was the most common AE mode and patient feedback was most frequently for determining exercise intensity.

**Conclusions:** Respondents clearly recognized the importance of AE in neuro-rehabilitation. Identified barriers to its use and limitations in screening procedures and selection of training parameters will be taken into consideration in the development of best practice recommendations.

**Keywords:** rehabilitation, stroke, aerobic exercise, screening, prescription

# Abstract: A022 – STANDARDIZED FUNCTIONAL CAPACITY OUTCOME MEASURES IN POST-OPERATIVE CARDIAC SURGERY: A SURVEY OF CURRENT CLINICAL PRACTICE AND DEVELOPMENT OF A CLINICAL PRACTICE GUIDELINE (CPG)

**Published:** 2012-05

**Authors:** T MacDonald

**Purpose/Objectives and Rationale:** To determine functional capacity outcome measure use among physiotherapists working with post-operative cardiac surgery patients and to develop evidence-based recommendations.

**Relevance to Physiotherapy Practice:** Evidence-based practice contributes to the effective and efficient delivery of health care. The use of standardized outcome measures ensures that physiotherapy practice is evidence-based.

**Materials and Methods:** The study was divided into three phases. The Cochrane Collaboration handbook for systematic reviews guided the systematic review process. The review included published studies of functional capacity outcome measures and their psychometric properties. In Phase II a survey was developed, based on the Revised Ottawa Model of Research Use, and distributed to all Canadian cardiac surgery centers to determine outcome measure use. In Phase III recommendations were developed by a Panel of Experts.

**Analysis:** Descriptive statistics were used to analyze the survey data.

**Results:** The review included 31 functional capacity outcome measures.Only 2.6% of respondents reported using outcome measures ”almost always” in clinical practice with the majority (51.3%) reporting using them ”occasionally”. The Six Minute Walk Test (6MWT) and the Modified Borg Rating Scale of Perceived Exertion (Borg) were the most frequently used outcome measures. The panel of experts recommended that the 6MWT, and the Borg be used with every cardiac surgery patient. Twelve of the outcomes that physiotherapist report using in practice were not recommended by the Panel of Experts.

**Conclusions:** Outcome measure use in clinical practice remains less than optimal. There is also a gap that exists in terms of the outcome measures being used in clinical practice and the recommendations of the Panel of Experts for the clinical practice guideline.

**Keywords:** outcome measures, post-operative, cardiac surgery

# Abstract: A036 – A SYSTEMATIC REVIEW OF THE HEALTH-RELATED QUALITY OF LIFE MEASURES FOLLOWING TOTAL KNEE ARTHROPLASTY

**Published:** 2012-05

**Authors:** SP Mehta, M Law, G Burgos-Martinez

**Affiliations:** School of Rehabilitation Science, McMaster University, Hamilton, Ont

**Purpose/Objectives and Rationale:** To perform systematic review and appraisal of common patient-reported outcomes used in measuring aspects of health-related quality of life following total knee arthroplasty.

**Relevance to Physiotherapy Practice:** Physiotherapy is an essential aspect of rehabilitation following total knee arthroplasty. Therefore, it is important that physiotherapists and other rehabilitation practitioners are aware of best outcome measures for assessing health-related quality of life following total knee arthroplasty.

**Materials and Methods:** A systematic literature search was performed to identify commonly used outcomes for assessing health- related quality of life following total knee arthroplasty. The selection of the outcomes was based on their frequency of use across studies that represent the highest evidence such as the systematic reviews and randomized controlled trials.

**Analysis:** A critical appraisal was performed using standardized guidelines for examining the conceptual basis, reliability, validity, responsiveness, and administrative issues of selected patient-reported outcomes.

**Results:** Seven outcomes were deemed eligible for this review and critical appraisal was performed for them. Five of them were either quality of life or health status measures and two were the measures of functional status. The measures of functional status had excellent psychometric properties in context of knee arthroplasty. The health status measures had low to moderate clinimetric properties though they had greater relevance for assessing health-related quality of life.

**Conclusions:** None of the outcomes reviewed comprehensively assess health-related quality of life following total knee arthroplasty. Qualitative studies can be conducted to expand the knowledge of patient-relvant health-related quality of life domains following total knee arthroplasty. Further, the clinimetric properties of the established health-related quality of life measures can be assessed in this patient group.

**Keywords:** health-related quality of life, total knee arthroplasty, outcome measures, clinimetric properties

# Abstract: A037 – EXAMINING UTILIZATION OF CONSTRAINT INDUCED MOVEMENT THERAPY IN CANADIAN NEUROLOGICAL REHABILITATION

**Published:** 2012-05

**Authors:** A Fleet, M Che, M MacKay-Lyons, D MacKenzie, S Page, S Boe

**Affiliations:** * School of Physiotherapy, Dalhousie University; † Dept. of Medicine, Division of Physical Medicine and Rehabilitation, Capital Health; ‡ School of Occupational Therapy, Dalhousie University; § Division of Occupational Therapy, The Ohio State University; ¶ Dept. of Psychology, Dalhousie University

**Purpose/Objectives and Rationale:** Constraint induced movement therapy (CIMT) combines repetitive task practice, behavioural re-training and restraint of the unaffected upper extremity (UE) to improve motor skills and function of the hemiparetic UE post- stroke. Traditional and adapted CIMT protocols have been described, which differ in treatment duration and intensity. Although evidence supports CIMT effectiveness, little is known about its clinical utilization. Thus, we investigated CIMT use in Canadian neurological rehabilitation.

**Relevance to Physiotherapy Practice:** Understanding clinical CIMT use, including factors affecting implementation, can guide research regarding CIMT feasibility and shape educational initiatives. Increasing the clinical feasibility and use of CIMT can improve rehabilitation outcomes.

**Materials and Methods:** An online survey was distributed to physical (PT) and occupational (OT) therapists practising in neurological rehabilitation. Questions were structured to gather respondent characteristics and CIMT utilization patterns (treatment parameters and barriers to implementation).

**Analysis:** Adjusted relative frequencies (percentage of total respondents/question) were used.

**Results:** An 11% response rate was observed (*n* = 338). 92% of respondents knew about CIMT, while 43% reported using CIMT. The majority of respondents using CIMT (88%) reported employing an adapted form of CIMT. Commonly identified barriers to use included ”lack of knowledge regarding the treatment” (48% of PTs) and ”treatment is not…part of routine practice in my facility” (45% of OTs).

**Conclusions:** Despite the majority of respondents knowing about CIMT, less than half reported using it. Identified barriers to CIMT use illustrate a gap between the evidence supporting CIMT and its clinical utilization. Recognizing barriers to CIMT use can aid in improving CIMT feasibility and identifying strategies to promote its clinical application.

**Keywords:** constraint induced movement therapy, stroke, neurological, rehabilitation, survey

# Abstract: A045 – DEVELOPMENT OF A CLINICAL GAIT ASSESSMENT PROTOCOL USING A PRESSURE MAT SYSTEM

**Published:** 2012-05

**Authors:** A Woznowski-Vu, R Preuss

**Affiliations:** * School of Physical and Occupational Therapy, McGill University; † Constance-Lethbridge Rehabilitation Centre site of the Centre de recherche interdisciplinaire en réadaptation (CRIR), Montreal, Que

**Purpose/Objectives and Rationale:** The purpose of this research project was to produce a clinical gait assessment protocol which optimizes the reliability of the measures using the minimum number of trials (balance between precision and efficiency), using the GAITRite system.

**Relevance to Physiotherapy Practice:** We expect this project to help improve the objectivity and effectiveness of rehabilitation health services.

**Materials and Methods:** Twenty-seven adults (*n* = 25; 2 excluded) were recruited from the patient population at the local rehabilitation centre. Participants attended 2 separate testing sessions (test-retest design), during which they were required to walk the length of the GAITRite walkway 4 or 5 times, depending on their endurance in gait. We determined the gait parameters of interest through a semi-structured focus group with the clinicians at the rehabilitation center.

**Analysis:** We analyzed the data using the Generalizability Theorem, with a target index of dependability (φ) of at least 0.8.

**Results:** The target index of dependability was exceeded (φ > 0.8) for the majority of gait parameters of interest using only one testing session, with only 10 data point. The greatest relative contributor to the variance was the “subject” variable, which explained on average 83.67% of the total variance (standard deviation = 10.32%).

**Conclusions:** Depending on the numbers of sessions and data points, either “time between heel on and toe on” or “base of support” is the most demanding variable. As such, in order to ensure reliable data for all variables, our initial recommendation is that, if fixed at a single testing session for φ > 0.8, a minimum of 48 strides should be acquired.

**Keywords:** generalizability theory, GAITRite, gait analysis, reliability, protocol

# Abstract: A046 – THE EXPERIENCE OF ANTICIPATING FALLING: IMPLICATIONS FOR PHYSIOTHERAPISTS

**Published:** 2012-05

**Authors:** JA Shaw, DM Connelly, CL McWilliam

**Affiliations:** * School of Physical Therapy; † Arthur and Sonya Labatt School of Nursing, The University of Western Ontario, London, Ont

**Purpose/Objectives and Rationale:** The purpose of this project was to explore the lived experience of anticipating falling of older adults living in the community.

**Relevance to Physiotherapy Practice:** For physiotherapists working with older adults to prevent falls, understanding the meaning older adults make out of their experience of anticipating falling will help to inform fall prevention interventions that are effective, meaningful, and relevant for older adult clients themselves.

**Materials and Methods:** Individual semi-structured interviews were completed with 9 older adults living independently in the community, ranging in age from 65–94. Interview questions focused on participants’ experience of falling and anticipating falling, eliciting reflections on the relation between fall risk, physical activity, and quality of life.

**Analysis:** Transcribed interviews were analyzed from a phenomenological perspective by three independent researchers. Phenomenological themes were identified and synthesized into an overall interpretation.

**Results:** The experience of anticipating falling meant confronting lived-identity in the context of aging. Participants described enacting lived-identity by portraying their self-image to those around them and by performing meaningful activities, both which contributed to quality of life. Participants described feeling vulnerable and/or anxious based on their experiences of falling and described taking caution in daily life as a means of continuing to perform meaningful activities.

**Conclusions:** The meaning of the experience of anticipating falling was described as an important influence on whether participants took caution in daily life. Physiotherapists should attend to the experiences and meaning older adult clients have with respect to falling when providing fall prevention interventions.

**Keywords:** fall risk, fall prevention, aging, phenomenology, identity

# Abstract: A054 – A FALL PREVENTION BEST PRACTICE INITIATIVE IN THE HOME CARE SETTING: A PILOT PROJECT

**Published:** 2012-05

**Authors:** J Shaw, K Sidhu, C Kearney, M Keeber, S McKay

**Purpose/Objectives and Rationale:** This project investigates the effects of implementing a comprehensive fall risk management best practice initiative on home care professionals’ fall risk assessment practice patterns. Objectives: To determine whether a falls prevention best practice initiative improves the fall risk assessment practices of home care physiotherapists (PT), nurses, and occupational therapists (OT).

**Relevance to Physiotherapy Practice:** By understanding how to improve fall risk assessment practices of physiotherapists in relation to other service providers, the unique contributions of physiotherapists to fall risk assessment and prevention in the home can be emphasized and advanced.

**Materials and Methods:** Home health service providers (10 nurses, 10 PT, 10 OT) completed an online survey regarding their current practice of fall risk assessment and prevention. A comprehensive fall risk screening tool was then implemented over a 6- week pilot period along with an educational session reviewing fall risk assessment and prevention best practices. Participants completed the same survey after the 6-week pilot period.

**Analysis:** A *t*-test was completed for each service provider group individually, as within-group changes were most operationally relevant for improving fall risk assessment practices.

**Results:** PTs and nurses assessed significantly more risks factors (*p* = 0.0055; 0.00048 respectively) post-intervention while OT’s did not (*p* = 0.11). PTs and nurses demonstrated the greatest improvements in assessing poor vision, poor hearing, and multiple medications as risk factors.

**Conclusions:** The fall risk management best practice initiative showed significant improvements for nurses and PTs. Further research is needed to explore reasons for the lack of OT improvement.

**Keywords:** best practice, fall risk, seniors health, community health

# Abstract: A001 – DEVELOPMENT OF A PHYSIOTHERAPY ENTRY-LEVEL CARDIORESPIRATORY CURRICULUM MODEL: PHASE I – A SYSTEMATIC REVIEW OF THE LITERATURE

**Published:** 2012-05

**Authors:** D Hopkins-Rosseel, K Bergsma, C Van Bavel

**Affiliations:** School of Rehabilitation Therapy, Queen’s University, Kingston, Ont

**Purpose/Objectives and Rationale:** To complete a systematic literature review as the first step in the development of an evidence- based, entry-level cardiorespiratory curriculum.

**Relevance to Physiotherapy Practice:** Through a 2008 Ministry initiative, rehabilitation academic programs in Ontario were offered increased funding to expand student enrolments. Many educational challenges have developed from resultant capital and operational changes.

**Materials and Methods:** The search strategy included 13 databases using 17 keywords in 2 content areas: adult education theories and cardiopulmonary educational models. In addition, association’s and university program’s publications were searched. Articles were evaluated for strength of evidence using a critical appraisal tool from the Published Guides and Recommended Questions for Appraising Reports of Medical Education Interventions (Reed D, 2005).

**Analysis:** Articles were assessed on 7 criteria: study purpose, rationale, objectives, design, intervention, evaluation, and results; the highest possible total score being 24 points. The curriculum models, instructional methods and content most commonly encountered were compared.

**Results:** The majority of the documents we retrieved were organizational reports or descriptive theory papers. Of the 8 studies retrieved, none scored as good quality (≥ 19), 3 scored as moderate quality (15–18) and 5 scored as poor quality (> 15). The most utilized adult education theories were adaptations of: Bloom’s (1956) educational taxonomy, Benner’s (1982) levels of proficiency and Newble’s (1986) learning styles.

**Conclusions:** There are few investigations into effective/efficient entry-level cardiorespiratory physiotherapy curricula. In addition, there are a wide range of educational models with few being used to guide curriculum development. A recommendation for a hybrid model, integrating the Canadian core curriculum with key theories, was proposed.

**Keywords:** curriculum, cardiorespiratory, education, model, theory, entry-level

# Abstract: A002 – TRACKING DIVERSITY OF STUDENTS ADMITTED INTO PHYSICAL THERAPY EDUCATIONAL PROGRAMS

**Published:** 2012-05

**Authors:** M Fricke, J Swinamer

**Affiliations:** Department of Physical Therapy, University of Manitoba, Winnipeg, Man

**Purpose/Objectives and Rationale:** This review tracks the diversity of students admitted into an entry-to-practice physical therapy program.

**Relevance to Physiotherapy Practice:** Changes in population demographics across Canada necessitate enhanced health care delivery to a culturally diverse society as well as health care human resources reflecting these realities. The evolution of physical therapy programs to the graduate level necessitates monitoring of the potential impact on student diversity.

**Materials and Methods:** This retrospective review utilized anonymous survey data from seven years of students admitted into a physical therapy program (*n* = 323), between 2003 and 2010. Characteristics were analyzed including gender, age, rural and ethnic backgrounds. Reasons for pursuing physical therapy as a career were also tabulated. Data from one cohort (*n* = 48) also included sexual orientation, socioeconomic and religious backgrounds.

**Analysis:** Descriptive trend analyses were performed by year and cohort.

**Results:** The majority of students admitted into this physical therapy program across all cohorts were female (87%); 20 to 25 years old (64%); and did not hold a prior degree. Approximately one third of the students reported a rural background; 25% self-identified as belonging to a visual minority; and 9% reported Aboriginal ancestry, compared to a provincial average of roughly 15%. The majority of students pursued physical therapy as a career due to personal experience. All respondents in the sub-cohort self- identified as heterosexual; average economic background was above the provincial median; 54% of these students observed religious beliefs.

**Conclusions:** The survey identified that physical therapy students do not generally represent the broader population. Targeted recruitment strategies are needed to address entrance diversity.

**Keywords:** admissions, diversity

# Abstract: A003 – OUTCOMES OF THE NORTHERN STUDIES STREAM AND REHABILITATION STUDIES PROGRAMS: A RETROSPECTIVE REVIEW AND ANALYSIS

**Published:** 2012-05

**Authors:** CS Winn, J Tryssenaar, BA Chisholm, JA Hummelbrunner, LS Kandler

**Affiliations:** * School of Rehabilitation Science, McMaster University, Hamilton; † Rehabilitation Studies Program, Northern Ontario School of Medicine; ‡ Lakehead University, Thunder Bay, Ont

**Purpose/Objectives and Rationale:** To identify and quantify factors affecting the rate of recruitment and retention of Northern Studies Stream or Rehabilitation Studies Program graduates to rural and/or remote areas of practice.

**Relevance to Physiotherapy Practice:** A shortage of physiotherapists in rural and/or remote practice areas negatively impacts health care delivery. The number of Northern Studies Stream or Rehabilitation Studies Program graduates who choose to live and work in a rural and/or remote area, and the extent to which participation in these programs or other factors contributed to their decision, is unknown.

**Materials and Methods:** An internet-hosted survey was administered to therapists who participated in the Northern Studies Stream or Rehabilitation Studies Program between 2002 and 2010. The survey consisted of 48 questions focusing on personal and professional demographics, post-graduate practice and experience, and factors affecting relocation and retention decisions.

**Analysis:** Simple frequencies and descriptive statistics were used. Respondents were compared using independent and paired samples *t*-tests, chi-square, and binary logistic regression.

**Results:** 281 respondents completed the survey. Multiple significant factors were identified, including that individuals raised in a rural or remote community were 2.60 times more likely to work in a rural or remote community after graduation and that 35.7% of individuals who participated in the Northern Studies Stream academic component reported moving to a rural or remote area.

**Conclusions:** Recruitment and retention of physiotherapists to rural and remote locations appears to be positively and significantly affected by the origins of the physiotherapist, and by the completion of both academic coursework as well as clinical education in a rural setting.

**Keywords:** recruitment, retention, clinical education, placement, rural, remote, northern, rehabilitation, physiotherapy

# Abstract: A004 – TEACHING PHYSICAL THERAPY USING VIDEO CONFERENCING TECHNOLOGY: EARLY EXPERIENCE WITH A RURAL, SATELLITE MASTER’S OF SCIENCE IN PHYSICAL THERAPY PROGRAM

**Published:** 2012-05

**Authors:** L Jasper, J Daniels, RG Haennel, R Johnson, B Martin

**Affiliations:** University of Alberta, Department of Physical Therapy, Edmonton, Alta

**Purpose/Objectives and Rationale:** In 2010, the University of Alberta’s (UofA) professional, entry-level Master’s of Science in Physical Therapy (MScPT) program in Edmonton established a sub-cohort of 10/80 students at a rural satellite campus (UofA Augustana) in Camrose, Alberta. Lecture, seminars and labs are presented via real-time video conferencing from Edmonton. This project focused on the experience of the students at the satellite campus in the first year of the program. It is part of a multifaceted evaluation of this distributed learning format.

**Relevance to Physiotherapy Practice:** Technology offers new opportunities for studying from almost anywhere. Assessment of the student engagement and the learning outcomes when technology is integral to educational delivery is essential.

**Materials and Methods:** Surveys, interviews and academic and clinical performance measures were utilized to gather data about the learning experience of the students at the satellite campus.

**Analysis:** Survey and interview data was analyzed using a qualitative approach in order to identify themes. Academic and clinical performance measurements for Augustana students were compared with those of students from the Edmonton cohort.

**Results:** Early findings suggest that the rural satellite students have embraced the program, the rural location and the distributed learning format. The academic and clinical performance of MScPT satellite students was equivalent to the performance of their counterparts attending classes in Edmonton.

**Conclusions:** Technology in education has the potential to dramatically change how and where we teach and learn. Providing physical therapy students the option to study in a rural locale may ultimately influence the supply of rural physical therapy clinicians.

**Keywords:** satellite campus, real-time video-conferencing, distributed learning, MScPT entry-level program

# Abstract: A016 – REGIONAL VARIATIONS IN CLINICAL PLACEMENTS FOR PHYSIOTHERAPY STUDENTS IN ONTARIO

**Published:** 2012-05

**Authors:** KE Norman, R Booth, B Chisholm, C Ellerton, W Jelley, A MacPhail, P Mooney, B Mori, L Taipalus, B Thomas

**Affiliations:** * Queen’s University, Kingston; † Northern Ontario School of Medicine, Thunder Bay; ‡ University of Toronto, Toronto; § University of Ottawa, Ottawa; ¶ University of Western Ontario, London; ** Northern Ontario School of Medicine, Sudbury; †† McMaster University, Hamilton, Ont

**Purpose/Objectives and Rationale:** To describe a year of physiotherapy student placements in Ontario relative to Ontario’s practising physiotherapists.

**Relevance to Physiotherapy Practice:** Clinical placements are essential to physiotherapy education. University programs continually strive to have sufficient quantity and diversity of clinical education opportunities. A quantitative description of placements by region can contribute to evidence-informed planning of clinical education.

**Materials and Methods:** Information about one year of physiotherapy students’ clinical placements was obtained from Ontario coordinators of physiotherapy clinical education. Information about practising physiotherapists was obtained from the College of Physiotherapists of Ontario. Physiotherapists were retained in the analysis if they reported direct patient care at a primary employment site in Ontario and at least 600 practice hours in their annual renewal. All placements and physiotherapists were attributed to one of the 14 Ontario local health integration networks (LHINs).

**Analysis:** A ratio for each LHIN was calculated using summed counts of student placements (numerator) and practising physiotherapists (denominator). Counts of placement types by setting, patient mix and practice area were also calculated for each LHIN.

**Results:** The range of ratios across LHINs was large. The five LHINs in which the programs are located had the highest ratios, from 0.92 to 0.38. The other nine LHINs had lower ratios, three at approximately 0.15. There was a wide mix of clinical placement types across LHINs.

**Conclusions:** There was a three- to six-fold difference across Ontario regions for participation of physiotherapists in students’ clinical education. There are opportunities to increase clinical education opportunities in Ontario if potential barriers for physiotherapists in those locations can be addressed.

**Keywords:** clinical education, student, health human resources

# Abstract: A018 – STROKE PATIENT EDUCATION: PROGRAM ASSESSMENT AND EVALUATION OF CLINICIANS’ PERCEPTIONS

**Published:** 2012-05

**Authors:** G Duval, S Ghali, A Nasirian, G Santillo, XL Yang, A Thomas, P McKinley, F Kaizer

**Affiliations:** McGill University, School of Physical and Occupational Therapy, Montreal, Que

**Purpose/Objectives and Rationale:** To integrate findings from a literature review and focus group to provide recommendations on how a rehabilitation stroke education program may be improved and how physical therapists can participate in these groups.

**Relevance to Physiotherapy Practice:** While patient and caregiver education is a vital element of stroke rehabilitation and physical therapy practice, a gap exists between best practice guidelines and current information provision practices.

**Materials and Methods:** First, a search of the scientific literature on stroke education was conducted. Second, a focus group with experienced clinicians from different disciplines was carried out to obtain practitioners’ perspectives on the characteristics of an ideal stroke education program.

**Analysis:** Analysis involved coding of the transcript and organization of excerpts into major themes.

**Results:** Six themes representing major aspects of a stroke education program emerged from the analysis: content, format & delivery, frequency & duration, evaluation, follow-up, and perceptions & beliefs of clinicians. For each theme, a number of evidence- based recommendations were made for clinicians providing the program. These included: provision of more practical information rather than medical facts, matching content delivery with stage of psychosocial readiness, provision of psychosocial support, and improving interprofessional communication.

**Conclusions:** This project contributes to the stroke education body of knowledge by providing evidence-based recommendations for improving a stroke education program. Physical therapists can use these findings to implement stroke education interventions or to improve an existing program. Future research is needed to determine if participation in such programs has health benefits, and ultimately, if it is preventative of secondary stroke.

**Keywords:** stroke education

# Abstract: A025 – EXPLORING THE CONCEPT OF LEADERSHIP FROM THE PERSPECTIVE OF PHYSICAL THERAPISTS IN CANADA

**Published:** 2012-05

**Authors:** L Desveaux, G Nanavaty, P Howell, R Sundar, J Ryan, M Verrier

**Affiliations:** University of Toronto, Department of Physical Therapy, Toronto, Ont

**Purpose/Objectives and Rationale:** To explore the concept of leadership from the perspective of Physical Therapists in Canada across settings of the workplace, the health care system and society.

**Relevance to Physiotherapy Practice:** With the increasing number of leadership roles available throughout the health care system from the ground level to administration, it is important to understand the perceptions of physical therapists with respect to leadership at all levels of health care delivery. Results from this study will help guide leadership development initiatives in Canada.

**Materials and Methods:** A quantitative, cross-sectional nationwide study was performed using a web-based survey administered to members of the Canadian Physiotherapy Association with a registered email address (*n* = 6156).

**Analysis:** Frequency distributions were obtained for all characteristics and chi-squared tests were utilized to determine significance (*p <* 0.05).

**Results:** 1875 members responded for a 30% response rate. Communication, professionalism and credibility were rated as being extremely important by the majority of respondents across all three settings. Practising in the private sector was a significant factor contributing to the perceived importance of business acumen. 79.6% of respondents self-declared as a leader with gender, primary work facility, and supervision of students identified as factors associated with self-declaration.

**Conclusions:** The top three characteristics that physical therapists perceived to be important differ from those reported among other health care professions. Future research is required to understand if the presence of multiple health care professionals in a workplace setting facilitates the opportunity for learning or if physical therapists feel overshadowed. As well, future studies should investigate whether individuals practising outside the private setting recognize the business aspects of their workplace.

**Keywords:** physical therapy, leadership, characteristics, leadership development, health care, Canada

# Abstract: A029 – READINESS TO PRACTICE IN CANADA: DEVELOPING A SELF-ASSESSMENT TOOL FOR INTERNATIONAL PHYSIOTHERAPISTS

**Published:** 2012-05

**Authors:** TL Dutton

**Affiliations:** Nova Scotia Community College, Halifax, N.S.

**Purpose/Objectives and Rationale:** To develop an on-line, interactive tool, designed to provide internationally-trained physiotherapists with information related to physiotherapy practice environments in Canada, and to provide international physiotherapists the ability to self-assess their present skills and abilities against established Canadian competencies.

**Relevance to Physiotherapy Practice:** Ensuring physiotherapists can make informed decisions related their own skills and abilities, as well as understanding the Canadian practice context, is critical for the success of internationally-trained physiotherapists considering practice in Canada.

**Materials and Methods:** Through government funding from Health Canada and Immigration, an on-line, self-assessment practice tool for internationally-trained physiotherapists was developed using existing tools from other disciplines. A collaborative team from information technology, the Canadian Physiotherapy Association, the Canadian Alliance of Physiotherapy Regulators, and a number of internationally-trained physiotherapists came together to develop the tool content and design.

**Analysis:** The on-line physiotherapist self-assessment tool was hosted on the regulatory and association websites for three months and data was gathered from users on the usability of the tool. Modifications were made to the tool based on the feedback gathered by users as well as using Google Analytics (web click-through analysis) to obtain objective feedback.

**Results:** A bilingual, three-part on-line tool for internationally-trained physiotherapists has been rolled out and is accessible from both the Alliance and Association websites.

**Conclusions:** Providing information to attract and recruit talented international physiotherapists to Canada will assist sustainable health human resource planning. Accessible, centralized information may enable internationally trained physiotherapists from other countries to make informed decisions related to the individual’s ability and interest to practice in Canada.

**Keywords:** competency, international, assessment

# Abstract: A041 – THE POST-PROFESSIONAL EDUCATION NEEDS OF OCCUPATIONAL THERAPIST AND PHYSIOTHERAPIST ASSISTANTS IN ONTARIO

**Published:** 2012-05

**Authors:** T Langendoen

**Affiliations:** University Health Network

**Purpose/Objectives and Rationale:** There are few post-professional educational opportunities specifically designed for occupational therapist assistants and physiotherapist assistants (OTA/PTA) in Ontario.

Objectives:

1. To determine how OTA/PTA identify and prioritize their post-professional education needs in terms of preference, areas of interest, and factors that influence their decision to attend courses;
2. The impact of demographic factors on these choices;
3. The preferred delivery format.

**Relevance to Physiotherapy Practice:** OTA/PTA work alongside Physiotherapists in a variety of settings and require similar continuing education to meet the needs of an ever changing health care environment.

**Materials and Methods:** A quantitative, descriptive, cross-sectional study design using an on-line questionnaire sent to OTA/PTA in Ontario with accessible e-mail addresses in October 2010.

**Analysis:** Descriptive statistics were used to describe demographics. Mean ± standard deviations were calculated for ranking factors.

**Results:** Demographically, respondents were primarily female, with an average age of 35.2 ± 9.8 years, working full-time for 8.1 ± 5.2 years in larger urban hospitals, rehabilitation settings or out-patient clinics, and providing care to orthopaedics, stroke, and geriatric or general medicine patients. Experienced OTA/PTA were more likely (*p* = 0.04) to work in rehabilitation settings, while less experienced respondents tended to work in acute care or out-patient settings. The preferred education delivery methods were hands-on skills, workshops and courses. The primary rationale for pursuing post-professional education was to upgrade or develop clinical skills, participate in inter-professional collaboration or network with colleagues.

**Conclusions:** Despite a variety of learning methods and course content identified by respondents, OTA/PTA recognize a need for ongoing education throughout their professional careers.

**Keywords:** education, post-professional education

# Abstract: A005 – VALIDITY OF THE NECK DISABILITY INDEX IN SCREENING IMPAIRMENT IN CHRONIC WHIPLASH

**Published:** 2012-05

**Authors:** J Marshall, C Coghill, C Pederson, C Blechinger, M Marshall

**Affiliations:** FIT for Active Living/Kinetik Program, Saskatoon City Hospital, Saskatoon, Sask

**Purpose/Objectives and Rationale:** To assess validity of the Neck Disability Index (NDI) in determining the extent of impairment of chronic whiplash-associated disorders (WAD) in occupational rehabilitation. Rationale: WAD clients without neurological involvement often demonstrate difficulty performing upper extremity functional tasks due to altered motor control of the cervical spine and shoulder girdle.

**Relevance to Physiotherapy Practice:** The Neck Disability Index may distinguish levels of impairment and disability for clients with signs and symptoms of mechanical neck pain (WAD II or less).

**Materials and Methods:** Patients referred to rehabilitation 3 or more months post motor vehicle collision were screened for neurological compromise (WAD III) and spinal fractures (WAD IV) prior to inclusion in the cross-sectional study. Neck Disability Index (NDI) and Disabilities of Arm, Shoulder and Hand (DASH) were administered on one occasion. Subjects were assessed on two occasions within 1 week for cervical spine and shoulder range of motion, cranio-cervical flexion test, posture, shoulder isometric strength and Numerical Pain Scale ratings (NPRS) by blinded physiotherapists.

**Analysis:** Correlation coefficients were evaluated for all variables. Participants were grouped according to established neck disability cut-off scores: mildly disabled (*n* = 10) or moderate/severely disabled (*n* = 27). Analysis of variance was performed.

**Results:** Significant correlations existed between the NDI, DASH, and NPRS (*r* = 0.45–0.77; *p* < 0.001). Post-hoc testing revealed clients with moderate/severe neck disability demonstrated significantly reduced cervical spine flexion (*p* < 0.01) and extension (*p* < 0.05) range associated with greater neck and shoulder pain (*p* < 0.001).

**Conclusions:** Convergent validity of the Neck Disability Index was substantiated for screening distinct impairment variables prior to functional testing in chronic WAD clients with mechanical neck pain.

**Keywords:** validity, Neck Disability Index, chronic whiplash

# Abstract: A006 – RASCH ANALYSIS OF THE UPPER EXTREMITY FUNCTIONAL INDEX (VERSION 2)

**Published:** 2012-05

**Authors:** CB Hamilton, BM Chesworth

**Affiliations:** * UEFI Study Group; † School of Physical Therapy, University of Western Ontario, London, Ont

**Purpose/Objectives and Rationale:** To evaluate the psychometric properties of the Upper Extremity Functional Index (version 2) using Rasch model criteria.

**Relevance to Physiotherapy Practice:** The Upper Extremity Functional Index is a 20-item self-report questionnaire used by physiotherapists to assess patients’ upper extremity function. Its psychometric properties have been supported by classical test theory, but have not been evaluated using Rasch analysis.

**Materials and Methods:** Upper Extremity Functional Index data (*n* = 263, 50.2% female, age range 14–83 years) were collected at the initial physiotherapy visit from patients with musculoskeletal upper extremity dysfunction. Conformity of the measure to the Rasch Partial Credit model was evaluated using the gold standard software for Rasch analysis.

**Analysis:** We assessed the data for overall fit, item and person fit, targeting, reliability, local response dependency, unidimensionality, and differential item functioning by gender, by whether the affected limb was the dominant limb, and by whether the affected limb was post- surgery. When expectations of the Rasch model were violated, we performed planned item rescoring, deletion and re-analysis.

**Results:** Rasch analysis supported a 14-item Upper Extremity Functional Index as a unidimensional measure of upper extremity function. It showed excellent person separation index reliability of 0.91. Two items exhibited differential item functioning by gender; but were retained after removal did not alter findings. Items removed from the 20-item measure were: hobbies/recreation/sports, food preparation, dressing, tying/lacing shoes, sleeping and throwing.

**Conclusions:** The results support the measurement properties, unidimensionality, and internal consistency of a 14-item Upper Extremity Functional Index. Future study should compare its validity with its predecessor.

**Keywords:** Rasch analysis, upper extremity function, outcome assessment

# Abstract: A008 – EARLY MOBILIZATION FOLLOWING MINI-OPEN ROTATOR CUFF REPAIR: A RANDOMIZED CONTROL TRIAL

**Published:** 2012-05

**Authors:** F Styles-Tripp, D Sheps, J Bury, M Bouliane, R Glasgow, D Otto, C Luciak-Corea, L Beaupre, R Balyk

**Affiliations:** * Department of Rehabilitation Medicine, Covenant Health Group; † Department of Surgery, University of Alberta; ‡ Department of Surgery, Covenant Health Group; § Department of Physical Therapy, University of Alberta, Edmonton, Alta

**Purpose/Objectives and Rationale:** Purpose: To compare early active range of motion (ROM) to 6 weeks of immobilization following a mini-open rotator cuff repair (MORCR). Objective: To evaluate the clinical outcomes following MORCR treated with early ROM compared to those in the standard immobilization protocol.

**Relevance to Physiotherapy Practice:** Can we allow early active range of motion following a MORCR without compromising the repair?

**Materials and Methods:** 187 patients with a full-thickness rotator cuff (RC) tear underwent MORCR and were randomized into either the immobilization (*n* = 90) or the early motion group (*n* = 97). Shoulder pain, ROM, abduction strength and health-related quality of life (HRQL) were assessed preoperatively. During the first 6 postoperative weeks, the early motion group began active ROM to help with activities of daily living, while the immobilization group remained in the sling. Shoulder ROM and pain were assessed at 6 weeks and 3 months postoperatively. At 6 months, abduction strength, HRQL, ROM and pain were assessed.

**Analysis:** Standard bivariate tests were performed at baseline to detect any systematic group differences. Two-way repeated measures ANOVA were performed using an intention to treat analysis.

**Results:** The groups were similar preoperatively in power, ROM, HRQL and pain (*p* > 0.10). Six-week ROM comparisons demonstrated that the early mobilization group had increased abduction (*p* = 0.03), flexion (*p* = 0.01) and scaption (*p* = 0.003); these differences disappeared by 3 months (*p* > 0.32). At 6 months, no group differences were found in power (*p* = 0.85) or HRQL (*p* = 0.72).

**Conclusions:** Early ROM showed neither significant benefits for minimizing long-term stiffness and pain nor any compromise of shoulder power or HRQL. Consideration should be given to allow controlled early active ROM following MORCR.

**Keywords:** rotator cuff tear, arthroscopically assisted, health related quality of life, functional outcome

# Abstract: A009 – COMPARING THE IMPACT OF ON-SITE VERSUS COMMUNITY-BASED EXERCISE ON AEROBIC CAPACITY AND ANTHROPOMETRIC MEASURES AT A LOCAL CARDIAC REHABILITATION CENTRE

**Published:** 2012-05

**Authors:** D Hopkins-Rosseel, S Carson, J Graham, W Hopman, T Parsons

**Affiliations:** * Queen’s University; † Hotel Dieu Hospital; ‡ Kingston General Hospital, Kingston, Ont

**Purpose/Objectives and Rationale:** To determine if individuals in the Community-Based exercise component of a cardiac rehabilitation program experience equal benefits to individuals in the On-Site group.

**Relevance to Physiotherapy Practice:** Cardiac rehabilitation is a cost-effective intervention which maximizes physical and psychological status in individuals with cardiometabolic disease (CACR, 2009). However, availability of On-Site exercise components presents a barrier to universal access.

**Materials and Methods:** Participants: Individuals (*n* = 508) completing 4 months at a cardiac rehabilitation centre between January 1st, 2004 and December 31st, 2009. Design: We performed a retrospective analysis of the relevant Vascular Health Protection Network patient data. Intervention: The On-Site (*n* = 277) group exercised twice per week in-centre, and once per week independently. The Community-Based (*n* = 231) group exercised independently three times per week. Outcomes determined at admission and discharge were: peak metabolic equivalents derived from graded exercise testing, waist circumference (cm), body mass index (kg/m^2^), and high density

**Analysis:** Between group differences (admission, discharge, change score) in each of the outcomes were analyzed using two-tailed independent *t*-tests (*p* < 0.05).

**Results:** The only difference in baseline characteristics between groups was the Community-Based group being triaged as lower risk for an adverse cardiovascular event. There were no significant differences in the amount of change from admission to discharge for any of the measures.

**Conclusions:** Cardiac rehabilitation participants who exercised On-Site experienced equivalent benefits with respect to the selected measures as compared to those who exercised in the community. Further research with high risk individuals in the Community- Based component should be undertaken to confirm the generalizability of these findings.

**Keywords:** cardiac rehabilitation, home-based, community-based, on-site, off-site, exercise

# Abstract: A011 – EXERCISE AS AN ANABOLIC INTERVENTION IN PEOPLE ON MAINTENANCE HEMODIALYSIS: A SYSTEMATIC REVIEW AND META-ANALYSIS

**Published:** 2012-05

**Authors:** A Sawant, T Overend

**Affiliations:** * Graduate Program in Health and Rehabilitation Sciences, Physical Therapy Field; † London Health Sciences Center, London, Ont; ‡ School of Physical Therapy, The University of Western Ontario

**Purpose/Objectives and Rationale:** People with end-stage kidney disease on maintenance haemodialysis (MHD) encounter multiple catabolic processes resulting in muscle atrophy and loss of muscle mass, a proxy for strength and function. Muscle wasting is one of the most significant predictors of morbidity and mortality in this population. The objective of this study was to systematically review the literature on the effect of exercise on muscle mass in adults on MHD.

**Relevance to Physiotherapy Practice:** Physiotherapy practice includes maintenance and/or restoration of impaired structure and function. Prevention of muscle wasting is thus important clinically and to reduce health care costs.

**Materials and Methods:** Electronic databases were searched from inception to September 2011. Inclusion criteria included randomized clinical trials published in English, with adult participants on MHD undergoing an exercise intervention, and muscle mass measured as an outcome.

**Analysis:** Included studies were combined in a meta-analysis. An effect size and 95% confidence interval (CI) of efficacy of exercise intervention was computed using a random effects model. Effect sizes for two moderator variables (type of intervention and measurement tool used for assessing muscle mass), were compared.

**Results:** The effect size of exercise intervention on muscle mass was statistically significant [0.256 (95% CI, 0.037–0.474)]. The differences in effect sizes for exercise intervention and type of measurement tool were not statistically significant.

**Conclusions:** These results indicate a small but significant anabolic effect of exercise interventions on muscle mass in people on MHD. Specifics of intensity and duration of exercise leading to increments in muscle mass requires further investigation.

**Keywords:** haemodialysis, exercise, muscle mass, skeletal muscle, systematic review

# Abstract: A015 – DELAY IN ACCESSING HEALTH CARE FOR KNEE OSTEOARTHRITIS – EXPLORING HEALTH PROFESSIONAL AND LAYPERSON PERCEPTIONS OF REASONS

**Published:** 2012-05

**Authors:** SS Prasanna, N Korner-Bitensky, S Ahmed

**Affiliations:** School of Physical and Occupational Therapy, McGill University, Montreal, Que

**Purpose/Objectives and Rationale:** In knee osteoarthritis (OA) the opportunity for non-surgical intervention is reduced due to time lost between symptom onset and diagnosis. This study was conducted to understand, from the perspective of various stakeholders, the reasons for delay and useful strategies to enhance early awareness of knee OA.

**Relevance to Physiotherapy Practice:** Knee OA has a high personal and societal burden. Early intervention can improve patient outcomes and prevent surgery. This study is a first step in a research agenda aimed at developing effective strategies to facilitate early intervention in knee OA.

**Materials and Methods:** Qualitative study using focus groups (*n* = 13), one with health professionals, the other with community- dwelling individuals. Question domains included: knowledge, attitudes, and beliefs about OA; experiences with people with OA; health care seeking behavior and access to services; and, suggested strategies to enhance public awareness.

**Analysis:** A grounded theory approach helped identify dominant themes.

**Results:** Reasons for delay from the layperson perspective included: lack of knowledge about risk factors and prevention, and a prominent belief that knee pain is expected with age. Reasons related to the health care system included: long wait times and frustration getting appointments. Health professionals indicated it is unclear which discipline should discuss prevention and risk factors. Strategies included: advocating a healthy lifestyle, developing prevention programs and use of celebrities to inform the public.

**Conclusions:** Multiple reasons for delays as well as strategies to counter them were identified. Understanding the factors contributing to delay is essential for building effective knowledge translation strategies.

**Keywords:** knee osteoarthritis, health attitudes, health seeking behavior, focus groups, health promotion

# Abstract: A017 – VALIDATION OF A PORTABLE DEVICE FOR MEASURING PERIODIC LIMB MOVEMENTS IN RESTLESS LEGS SYNDROME

**Published:** 2012-05

**Authors:** MJ O’Donovan, H Driver

**Affiliations:** * Queen’s University; † Kingston General Hospital, Kingston Ont

**Purpose/Objectives and Rationale:** To establish whether the auxiliary EMG channel of a portable monitor validated for screening of obstructive sleep apnea — Braebon Medibyte¯ — reliably records limb movements during sleep in comparison with polysomnography.

**Relevance to Physiotherapy Practice:** Restless Legs Syndrome (RLS), a sensory motor condition causing significant discomfort and sleep disturbance, is under-recognized and under-treated. Recent studies show improvements with exercise and infrared therapy. The presence of Periodic Limb Movements (PLMs) aids in the evaluation of RLS. Objective assessment of RLS is often inaccessible due to a lack of efficacious methods to measure PLMs outside of the sleep laboratory. With a reliable portable monitor, physical therapists could measure PLMs as an aid to diagnosis and assessing treatment response.

**Materials and Methods:** The Medibyte¯ was tested simultaneously with polysomnography during overnight sleep studies at Kingston General Hospital with 28 participants referred for screening of sleep disorders. PLMs were scored using standard criteria.

**Analysis:** Correlation between methods was examined using the Wilcoxon Signed-ranks Test for nonparametric data. Reliability of the device was tested with Intraclass Correlation Coefficient and agreement by Bland-Altman analysis

**Results:** The median PLM Index by polysomnography (9.5/h, Interquartile Range 2.6–21.6) and Medibyte¯ (16.0/h, Interquartile Range 6.4–34.1) were not significantly different. Intraclass Correlation Coefficient (absolute agreement) was 0.84. Bland-Altman analysis demonstrated a mean bias of +3.7(± 12.75) for the Medibyte¯ with limits of agreement of -21.3 to 28.7.

**Conclusions:** These data suggest there is sufficient agreement between PLM Index collected by Medibyte¯ and polysomnography to support the use of the Medibyte¯ for measuring PLMs.

**Keywords:** restless legs syndrome, periodic limb movements, portable monitor

# Abstract: A019 – REPETITIVE TRANSCRANIAL MAGNETIC STIMULATION IMPROVES HANDWRITING IN INDIVIDUALS WITH PARKINSON DISEASE

**Published:** 2012-05

**Authors:** BK Randhawa, BG Farley, LA Boyd

**Affiliations:** * Graduate Program in Rehabilitation Sciences; † Department of Physiology, University of Arizona, Tucson, Arizona; ‡ Department of Physical Therapy, University of British Columbia, Vancouver, B.C.

**Purpose/Objectives and Rationale:** Parkinson Disease is characterized by hypometric and bradykinetic movements resulting from loss of dopaminergic neurons in the substantia nigra. Parkinson’s disease leads to decreased activation of the supplementary motor area (SMA); the net result of these changes is a poverty of movement. The present study determined the impact of 5 hertz repetitive transcranial magnetic stimulation (rTMS) over the SMA on a fine motor movement, handwriting (writing cursive “l’s), and on cortical excitability, in individuals with Parkinson’s disease (PD).

**Relevance to Physiotherapy Practice:** The data reported here represent a first step in determining the potential utility of rTMS, which can be combined with therapeutic exercises to further enhance the benefit in individuals with PD.

**Materials and Methods:** In a crossover design, ten individuals with PD were randomized to receive either 5 hertz or control stimulation over the SMA. Immediately following brain stimulation right handed writing was assessed.

**Analysis:** For each kinematic variables of handwriting, quantified by ScriptAlyzer software, 2 by 2 ANOVAs were performed. The same statistical test was employed with resting motor threshold to index cortical excitability.

**Results:** 5 hertz stimulation increased handwriting vertical size and diminished axial pressure. In addition, 5 hertz rTMS significantly decreased the threshold for excitability in the primary motor cortex.

**Conclusions:** These data suggest that in the short-term 5 hertz rTMS benefits functional fine motor task performance, perhaps by altering excitability across a network of brain regions. Further, these data may provide the foundation for a larger investigation of the effects of non-invasive brain stimulation over SMA in individuals with PD.

**Keywords:** repetitive transcranial magnetic stimulation, supplementary motor area, Parkinson Disease, motor control, hand function

# Abstract: A020 – THE EFFECT OF ALTERED ENVIRONMENTAL LIGHT CONDITIONS ON SPATIO-TEMPORAL GAIT PARAMETERS, GAIT ADAPTATION, DYNAMIC BALANCE AND FEAR OF FALLING USING BODY-WORN SENSOR TECHNOLOGY

**Published:** 2012-05

**Authors:** R Henderson, B Najafi, J Bansberg, G Druja, S Hinch, A Sikkema

**Affiliations:** Rosalind Franklin University of Medicine and Science, North Chicago, Ill., USA

**Purpose/Objectives and Rationale:** The purpose of this study was to investigate differences in spacio-temporal gait parameters (stride velocity, stride length, gait symmetry, gait cycle time, gait initiation time), gait adaptation time, dynamic balance (center of mass displacement) and fear of falling during ambulation in two conditions; light and near dark. We hypothesized deterioration of spacio-temporal gait parameters and dynamic balance, and increase in gait adaptation time and fear of falling in the near dark condition. Falls are prevalent in the elderly yet little research has quantitatively investigated changes in gait, balance and fear of falling in altered light conditions.

**Relevance to Physiotherapy Practice:** This study informs assessment and treatment of gait and balance problems, and fall prevention.

**Materials and Methods:** A quasi-experimental, single group, pretest-posttest design was used. The sample consisted of twenty volunteer non-pathologic subjects, 22–46 years of age (mean 24.5). Gait and balance variables (named previously) were measured continuously with body- worn sensor (3-D gyroscope) technology, permitting measurement in an authentic situation. Subjects walked a straight/level 40’ pathway (one trial) for five trials in each of the two conditions. Fear of falling was assessed via questionnaire.

**Analysis:** The Dependent *t*-test investigated intra-subject differences between conditions. ANOVA and Sheffé tests investigated intra-condition differences between trials. The Wilcoxon Signed Rank Test investigated fear of falling difference between conditions.

**Results:** As hypothesized, statistically significant differences were identified for all spatio-temporal gait parameters, gait adaptation time, dynamic balance and fear of falling (*p* < 0.05).

**Conclusions:** This study strongly suggests that diminished light compromises gait and balance, and increases fear of falling, thus increasing the risk of falls.

**Keywords:** falls, gait, balance, body-worn sensor

# Abstract: A021 – RANDOMIZED CONTROLLED TRIAL OF EFFECT OF VIRTUAL REALITY VERSUS PHYSICAL TRAINING ENVIRONMENTS ON ARM MOTOR RECOVERY IN CHRONIC STROKE

**Published:** 2012-05

**Authors:** SK Subramanian, CB LourenÇo, G Chilingaryan, H Sveistrup, MF Levin

**Affiliations:** * School of Physical and Occupational Therapy, McGill University, Montreal, Que; † Department of Social and Preventive Medicine, Université de Montréal, Montreal, Que; ‡ Faculty of Health Sciences, University of Ottawa , Ottawa, Ont; § Feil/Oberfeld Research Centre, Jewish Rehabilitation Hospital site of Center for Interdisciplinary Research in Rehabilitation (CRIR), Laval, Que

**Purpose/Objectives and Rationale:** Despite recent interest in the use of virtual reality environments (VEs) for post-stroke arm motor rehabilitation, advantages of training in a VE over that in a physical environment (PE) have not been elucidated. We compared kinematic and clinical outcomes of dose-matched upper-limb training between a 3D-VE and PE in chronic stroke subjects.

**Relevance to Physiotherapy Practice:** This study will inform neurological physical therapy practice on the effectiveness of different training environments.

**Materials and Methods:** Thirty two participants were randomized to practice pointing tasks in two environments (PE/VE) and further subdivided by motor impairment into mild and moderate-to-severe groups. Participants pointed in a random sequence to 6 targets (12 trials/target, 4wk, 12 sessions). Feedback on precision, movement speed and trunk displacement was provided in both environments. Pre, post and follow-up kinematic and clinical assessments were performed.

**Analysis:** Data were analyzed using mixed-model ANOVAs.

**Results:** Overall, both groups increased movement speed and shoulder horizontal adduction range and improved motor function (Wolf Motor Function) at post- and follow-up testing (*p* < 0.05). Shoulder horizontal adduction increased at post-test (13°, *p* < 0.01) and shoulder flexion range increased at both post-test (6°, *p* < 0.05) and follow-up (13°, *p* < 0.01) only in VE. Group analysis revealed that moderate-to-severe group training in VE improved on Motor Activity Log (MAL) at post-test (0.5, *p* < 0.05). The moderate-to-severe group training in PE increased elbow extension (11°, *p* < 0.05) with greater trunk displacement (41 mm, *p* < 0.05) at follow-up. They also improved on MAL at follow-up (0.4, *p* < 0.05). The mild group in VE increased elbow extension range (30°, *p* < 0.01) at follow-up.

**Conclusions:** VE training led to more adaptive pattern of arm motor recovery possibly due to better use of feedback.

**Keywords:** feedback, recovery, rehabilitation, kinematics, upper-limb

# Abstract: A023 – PATIENT EDUCATION BEFORE LUNG SURGERY: WHAT PATIENTS PERCEIVE AS IMPORTANT

**Published:** 2012-05

**Authors:** J King, P Chamberland, A Agar,, R Leger, R Michaels, R Poitras, A Rawji, D Skelton, M Warren

**Affiliations:** * University of Ottawa; † The Ottawa Hospital, Ottawa, ON; ‡ Canadian Forces,Quebec City, PQ; § Montreal Children’s Hospital, Montreal, PQ; ¶ Stanton Territorial Hospital/Health Authority, Yellowknife, NWT

**Purpose/Objectives and Rationale:** To investigate the preoperative patient education needs of adults undergoing surgical lung cancer resection.

**Relevance to Physiotherapy Practice:** Physiotherapists routinely provide preoperative education to prepare patients for surgery and to facilitate postoperative participation. Preoperative education is known to improve patient outcomes, decrease anxiety, frustration and postoperative complications. Yet, there can exist a discrepancy between the information that physiotherapists and other team members provide and the information patients perceive as important to their care.

**Materials and Methods:** Using a mixed-methods approach, 15 adults who underwent surgical removal of a lung cancer participated in semi-structured interviews preoperatively and postoperatively (one to four weeks after hospital discharge). Following preliminary qualitative analysis, 9 of them were also asked about their pain using the McGill Pain Questionnaire.

**Analysis:** Using a generalized qualitative approach, participants’ interviews were read individually. Core categories were determined by using constant comparison, and then categories were compared to determine emerging themes. Quantitative analysis included frequency counts.

**Results:** Overall themes that emerged included satisfaction with the information participants received, feeling prepared for surgery, importance of physiotherapy in their recovery and limited explanation of postoperative pain. Pain descriptors commonly used included sharp, stabbing and tender.

**Conclusions:** Future work will explore physiotherapists and other team members’ description and explanation of pain preoperatively and the actual description of pain experienced by patients postoperatively. This could help physiotherapists better prepare patients preoperatively and thus, improve their post operative recovery.

**Keywords:** patient education, lung cancer, lung surgery, pain

# Abstract: A024 – THE EFFICACY OF THREE PORTABLE OXYGEN CONCENTRATORS IN SUSTAINING OXYGEN CONCENTRATION GREATER THAN 90% DURING EXERTION IN PATIENTS WITH CHRONIC LUNG DISEASE

**Published:** 2012-05

**Authors:** L Lavallée, C LeBlanc, D McKim, J King, A Woolnough

**Affiliations:** The Ottawa Hospital Rehabilitation Centre, Ottawa, Ont

**Purpose/Objectives and Rationale:** The use of portable oxygen concentrators (POCs) is essential to maintain and increase mobility in individual with chronic lung disease, but not all are equally effective. The purpose of this study was to compare the capability of three POCs, in maintaining SpO_2_ ≥ 90% on exertion in patients with Chronic Lung Diseases.

**Relevance to Physiotherapy Practice:** Physiotherapists are increasingly involved in the use and recommendation of POCs and need to know which POC’s is most effective in responding to patients’ oxygen requirements.

**Materials and Methods:** Six minute walk tests (6-MWTs) were performed in 23 patients with documented room air exertional SpO_2_ ≤ 85%. A control walk was performed with the participants’ current oxygen system set at their prescribed exertional flow rate. They then performed 6-MWTs with the 3 POCs (Evergo, Eclipse 3, iGo), randomly assigned using a sequence generator, and set at their maximum pulse dose. Oxygen saturation and pulse rate were measured throughout.

**Analysis:** Statistical analysis was performed using SPSS Version 18 for Windows using a one-way ANOVA and Tukey’s Post-Hoc.

**Results:** Twenty-three patients participated (14 male, 9 female, average age 65.8 ± 8.5 years old), 20 had Chronic Obstructive Pulmonary Disease, three had Idiopathic Pulmonary Fibrosis. The Eclipse 3 resulted in significantly higher Pre and Post exercise SpO_2_ and increased total time and distance walked.

**Conclusions:** Only the Eclipse 3 resulted in SpO_2_ > 90%. Its clinical performance, total time and distance walked also exceeded the other POCs, which may translate to improved cardiopulmonary endurance, activities of daily living and quality of life.

**Keywords:** COPD, 6 MWT, pulmonary rehabilitation, portable oxygen concentrator

# Abstract: A028 – THE PREVALENCE OF DISABILITY IN THE NORTH WEST REGION OF CAMEROON

**Published:** 2012-05

**Authors:** L Cockburn, J Wango, E Benuh, S Cleaver

**Affiliations:** * Department of Occupational Therapy and Occupational Science; § Graduate Department of Rehabilitation Science, University of Toronto, Toronto, Ont; † Bamenda Coordinating Center for Studies in Disability and Rehabilitation; ‡ Cameroon Baptist Convention Health Board, Bamenda, Cameroon

**Purpose/Objectives and Rationale:** To determine the prevalence of disability and specific-categories of activity limitations and participation restrictions in the North West Region of Cameroon.

**Relevance to Physiotherapy Practice:** The World Disability Report has highlighted the large numbers of persons with disabilities residing in low-income countries and the disadvantages faced by this population. Rehabilitation has the potential to improve the functional status and quality of life for many of these people, but in order to most effectively allocate scarce resources we must know more about the situations of these potential beneficiaries.

**Materials and Methods:** The study was planned and carried out using inclusive, participatory processes appropriate to the context, and engaged several key people in the region and externally. The survey design was a household survey using a multi-stage cluster design. The International Classification of Functioning, Disability, and Health (ICF) was used as an organizing framework.

**Analysis:** Epi-Info was used to calculate the prevalence according to the study’s cluster design.

**Results:** The overall prevalence of disability was 6.9% (95% CLs = 5.7–8.2%). We also estimated disabilities in several domains, such as learning and applying knowledge, managing self-care, and mobility. In each of these areas, we found that several thousands of people are living with disabilities.

**Conclusions:** This important study confirms that thousands of people are affected by impairments and disabilities in this region of Cameroon. The information gained from this study should encourage government and non-government programs and services to provide more relevant and needed services to the population.

**Keywords:** disability, prevalence, population health research, Cameroon, global health

# Abstract: A030 – THE EFFECT OF PELVIC FLOOR MUSCLE EXERCISE ON STRIATED URETHRAL SPHINCTER MORPHOLOGY IN OLDER WOMEN WITH STRESS URINARY INCONTINENCE

**Published:** 2012-05

**Authors:** SJ Madill, S Pontbriand-Drolet, A Prud’homme-Delage, A Tang, C Dumoulin

**Affiliations:** Centre de recherche de l’institut universitaire de gériatrie de Montréal, Que

**Purpose/Objectives and Rationale:** Pelvic floor muscle (PFM) training increases PFM volume and urethral pressure, but it is not known if the training also affects the morphology of the striated urethral sphincter (SUS). The purpose of this study was to determine the effect of PFM exercise on the SUS in older women with SUI.

**Relevance to Physiotherapy Practice:** Physiotherapists are primary providers of PFM rehabilitation services. In order to provide the most effective treatments, we must understand how they work.

**Materials and Methods:** Eighteen women with SUI, mean age 67.6 (± 5.6) years, were recruited. Before and after the intervention they completed a three-day bladder diary, the Urogenital Distress Inventory (UDI) and the Incontinence Impact Questionnaire (IIQ). SUS morphology was evaluated with MRI recorded at rest in the axial plane using a Seimens 3.0T Magnetom Trio. The thickness of the SUS was measured from the images, the area and volume were calculated according to Morgan et al.’s method. The intervention consisted of 12 weekly, one-hour PFM group exercise classes.

**Analysis:** The data were analyzed using repeated measures analyses of variance, α ≤ 0.05.

**Results:** Incontinence symptoms improved on all three measures used: diary *p* = 0.005, UDI *p* < 0.001, and IIQ *p* = 0.015. SUS thickness increased: 2.97 (± 0.92) to 3.57 (± 0.84) mm, *p* = 0.049. The sphincter’s area and volume also increased, but not significantly (*p* = 0.089 and *p* = 0.075, respectively).

**Conclusions:** PFM training for older women with SUI concurrently trains the SUS and produces sphincter hypertrophy. This appears to be one mechanism by which PFM exercise improves continence.

**Keywords:** pelvic floor muscle training, stress urinary incontinence, urethral sphincter, magnetic resonance imaging

# Abstract: A031 – VALIDATION OF A CLASSIFICATION FOR PATIENTS WITH PATELLAR INSTABILITY

**Published:** 2012-05

**Authors:** LA Hiemstra, M Lafave, S Kerslake, M Heard, G Buchko

**Affiliations:** * Banff Sport Medicine, Banff; † Mount Royal University, Calgary, Alta

**Purpose/Objectives and Rationale:** Patients with patellar instability present with a wide range of etiologies and symptoms as well as varied anatomic and neuromuscular characteristics. For patella instability, the authors propose that two main subsets of patients present. This study introduces a novel classification system, WARPS (Weak, Atraumatic, anatomy Risky, Pain, and Subluxation) and STAID (Strong, Traumatic, Anatomy normal, Instability and Dislocation) The purpose of this study was to demonstrate inter- observer reliability of the classification continuum of WARPS and STAID for patella instability.

**Relevance to Physiotherapy Practice:** Identification of two distinct patellar instability subsets will enable physiotherapists to assess and then tailor treatment programmes to the unique anatomic and neuromuscular characteristics.

**Materials and Methods:** Twenty-five consecutive patients with a confirmed diagnosis of patellar instability were included. Each patient underwent a thorough history and physical examination concurrently with 3 assessors (orthopaedic surgeon, physiotherapist and athletic therapist). Each characteristic of the WARPS-STAID classification continuum was graded independently by each examiner on a 10 cm VAS. Each assessor also assigned a cumulative WARPS-STAID score to each patient.

**Analysis:** Intraclass Correlation Coefficient (ICC 3,3) was calculated for each pair of characteristics on the continuum and for the cumulative scores.

**Results:** Intraclass Correlation Coefficient (3,3) of the WARPS-STAID classification continuum were W-S = 0.92; A-T = 0.95; R-A = 0.81; P-I = 0.84; and S-D = 0.91. The ICC (3,3) of the composite score was 0.84.

**Conclusions:** These results demonstrate strong reliability of the classification system, WARPS-STAID for patients with patella instability. The classification system reliably differentiates between two distinct subsets of patella instability patients.

**Keywords:** patellar instability, classification, inter-observer reliability

# Abstract: A032 – INITIAL VALIDITY AND RELIABILITY OF THE BANFF PATELLAR INSTABILITY INSTRUMENT (BPII)

**Published:** 2012-05

**Authors:** LA Hiemstra,, M Lafave, S Kerslake, M Heard, G Buchko, N Mohtadi

**Affiliations:** * Banff Sport Medicine, Banff; † Mount Royal University, Calgary; ‡ University of Calgary, Calgary, Alta

**Purpose/Objectives and Rationale:** Patellar instability is a common problem yet there are currently no published, patient-reported outcome-measures that are disease- specific for this population. The purpose of this study was to determine if the Banff Patellar Instability Instrument (BPII) is a valid and reliable outcome for measuring quality of life in subjects with recurrent patellar instability.

**Relevance to Physiotherapy Practice:** Establishing the BPII as a valid and reliable tool will provide a disease-specific outcome- measure for use by physiotherapists.

**Materials and Methods:** A modified Ebel procedure was initially employed to validate the content of the BPII using an international expert panel. One hundred and twenty patients with a confirmed diagnosis of patella instability completed the BPII at the initial orthopaedic surgeon consult, 6 month and 12 month follow-up appointments.

**Analysis:** As a measure of internal consistency, Cronbach’s Alpha was utilized to assess how reliably the 32-items of the BPII measured a similar construct (patellar instability). Data from each item of the BPII, and the cumulative score, was used in the Reliability Coefficient analysis.

**Results:** Content validity was clearly established as each item in the BPII achieved a minimum of 83.3% (range 83.3–100%) agreement for relevance among the expert panelists. The average agreement was 96.9% and 24/32 questions achieved 100% agreement. Reliability of the BPII was established at the initial orthopaedic consult (α= 0.91), 6 months post-operatively (α = 0.96) and 12 months post-operatively (α = 0.96).

**Conclusions:** This study has established the BPII is valid and reliable in patients with recurrent patellar instability as well as patients who have had a patellar stabilization procedure.

**Keywords:** patellar instability, quality of life, validity, reliability

# Abstract: A035 – AN EXTENDED EXERCISE REHABILITATION PROGRAM POST HIP FRACTURE IMPROVES PATIENTS’ PHYSICAL FUNCTIONING: A SYSTEMATIC REVIEW AND META-ANALYSIS

**Published:** 2012-05

**Authors:** M Auais, O Eilayyan, N Mayo

**Affiliations:** School of Physical & Occupational Therapy, Faculty of Medicine, McGill University, Que

**Purpose/Objectives and Rationale:** Although the principal goal of management for survivors of hip fractures is a return to pre- event functional level, most survivors fail to regain their former levels of autonomy. One of the most effective strategies to mitigate the fracture’s consequences is exercise. Our objective is to review and quantify the reported effect of an extended exercise rehabilitation program offered beyond the regular rehabilitation period on improving physical functioning for hip fractures patients.

**Relevance to Physiotherapy Practice:** The positive effect of exercise interventions at the acute and the sub-acute phases is well- known; however, the effect of an exercise program for patients in the community is unclear.

**Materials and Methods:** The Cochrane, PubMed, CINAHL, PEDro, EMBASE databases, and reference lists of articles were searched from inception to October, 2010. All randomized controlled trials comparing extended exercise programs to usual care where the participants are community-dwelling were included. A structured form was used for data collection and two reviewers conducted each step independently.

**Analysis:** The data from included studies were summarized, tabulated, and then pooled for nine functional outcomes. Hedges’ g effect size and 95% confidence intervals were used to describe the effect of exercise programs.

**Results:** We found significant positive impact on: knee-extension strength for affected and none-affected sides, physical performance-based tests, balance, fast gait speed, and SF36-physical functioning.

**Conclusions:** To our knowledge this is the first meta-analysis to provide evidence that an extended exercise rehabilitation program for patients with hip fractures has a significant impact on functional abilities. The focus of future research should go beyond just effectiveness and study cost-effectiveness of extended programs.

**Keywords:** systematic review, extended rehabilitation exercise program, hip fracture, physical therapy

# Abstract: A038 – ADAPTATION OF RESPIRATORY BREATHING PATTERNS POST-STROKE IN RESPONSE TO AN INCREASE IN WALKING SPEED

**Published:** 2012-05

**Authors:** C Otfinowski, J Fung, P Smallhorn, d’Aux N Diez, He F Shan, L Li, Y Ren, C Perez, J Spahija

**Affiliations:** * Feil/Oberfeld/CRIR Research Centre, Jewish Rehabilitation Hospital; † School of Physical and Occupational Therapy, McGill University; ‡ Research Center, Hôpital du Sacre-Cœur de Montréal, Université de Montréal; Montreal, Que

**Purpose/Objectives and Rationale:** Respiratory dysfunction can occur post-stroke as a result of impairment in chest wall expansion due to hemiparesis. Healthy people increase tidal volume with increased walking speed, but it is not known whether increasing gait speed leads to an increased tidal volume post-stroke. The purpose of this study is to assess, in a pilot study, the respiratory response during quiet and deep breathing during slow and fast walking speeds in people with chronic stroke, as compared to age- and sex-matched healthy controls.

**Relevance to Physiotherapy Practice:** This study improves our understanding of the mechanisms controlling breathing during walking in individuals post-stroke.

**Materials and Methods:** Five chronic stroke survivors and five healthy age- and sex-matched controls performed one session of four randomized walking trials, with a combination of comfortable or fast walking speeds, while breathing quietly or deeply. Parameters of the respiratory and gait cycles were measured using a pneumotachograph (tidal volume, respiratory time) and the VICON motion capture system (stance and swing time, gait velocity) combined with a self-paced treadmill.

**Analysis:** Descriptive statistics were used to calculate differences within and between participants.

**Results:** Preliminary results show that variable breathing pattern responses occur as a result of changes in walking speed in five people with stroke. Only one individual post-stroke compared to all control subjects, increased tidal volume with increases in gait speed. Thus, increased gait speed did not have a similar effect on breathing pattern in all individuals post-stroke.

**Conclusions:** The inability of people post-stroke to increase tidal volume during fast walking suggests a compromise in respiratory function post-stroke.

**Keywords:** respiratory, stroke, gait speed, breathing pattern, gait

# Abstract: A039 – TIMING OF MUSCLE PREACTIVATION DURING LANDING FROM A JUMP IN ATHLETES WITH FUNCTIONAL ANKLE INSTABILITY

**Published:** 2012-05

**Authors:** M Sadeghi, S Ebrahimi, N Maroufi, AA Jamshidi

**Affiliations:** Kerman University of Medical Sciences, Kerman, Iran

**Purpose/Objectives and Rationale:** To evaluate the feedforward mechanism of motor control system during landing from a jump in volleyball players with functional ankle instability (FAI)

**Relevance to Physiotherapy Practice:** Altered motor program may underlie the instability of the ankle but there is no sufficient evidence in this area.

**Materials and Methods:** In this cross-sectional case control study, sixteen athletes with FAI and sixteen asymptomatic controls participated. We measured onset times and sequence of Gluteus Maximus (G.Max), Gluteus Medius (G.Med), Vastus Medialis (VM), Peroneus Longus (PL) and Medial Gastrocnemius (MG) muscles preactivation using Surface EMG during a jump-landing task.

**Analysis:** The independent *t*-test and the one-way ANOVA used for statistical analysis.

**Results:** Preactivation onset times of G.Med and VM muscles during landing in the FAI group were significantly greater than those of the healthy group (*p* < 0.05). G.Max, PL and MG preactivation onset times showed no significant differences between the two groups (*p* > 0.05). In the healthy group, G.max, G.med, VM were activated simultaneously while PL and MG were activated later than other muscles but at the same time. In the FAI group, G.max onset was earlier than other muscles whereas G.med, VM, PL, MG were activated concurrently.

**Conclusions:** The preactivation onset times of proximal muscles and the sequence of muscles preactivation alter in athletes with FAI. Altered timing of feedforward mechanism may be associated with repeated events of ankle instability during landing.

**Keywords:** muscle preactivation, landing, ankle instability

# Abstract: A040 – EFFECTIVENESS OF AN 8-WEEK POSTERIOR SHOULDER STRETCHING PROGRAM ON VARSITY-LEVEL OVERHEAD ATHLETES: A RANDOMIZED CONTROLLED TRIAL

**Published:** 2012-05

**Authors:** JC Chepeha, DJ Magee, M Bouliane, D Sheps, S Warren, L Beaupre

**Affiliations:** Department of Physical Therapy, Faculty of Rehabilitation Medicine, Edmonton, Alta

**Purpose/Objectives and Rationale:** To determine whether an 8-week posterior shoulder stretch was effective in increasing dominant arm internal rotation (IR) and horizontal adduction (HAd) range of motion (ROM) in a group of overhead athletes identified as having tightness of their posterior shoulder structures.

**Relevance to Physiotherapy Practice:** Individuals involved in repetitive overhead rotation demonstrate altered shoulder rotation patterns; namely increased ER- and decreased IR-ROM. Deficits in IR-ROM are believed to be an important risk factor in the development of shoulder pathology.

**Materials and Methods:** Thirty-seven male and female varsity-level overhead athletes in volleyball, swimming and tennis, identified with an IR-ROM deficit ≥ 15°, were randomized into a stretch or control group. Athletes assigned to the stretch group performed the “sleeper stretch” daily for 8 weeks.

**Analysis:** Independent *t*-tests determined whether significant differences existed between the 2 groups’ IR- and HAd-ROM after 8- weeks and 2-way repeated measures ANOVA tests determined the rate of ROM change over the 8-week evaluation.

**Results:** Statistically significant differences were found between the stretch and control groups’ IR- and HAd-ROM at 8 weeks (*p* = < 0.001 and *p* = 0.003 respectively) compared to baseline (0 weeks) (*p* = 0.188 and *p* = 0.819 respectively). Significant changes in IR-ROM were detected in the stretch group as early as 4 weeks (*p* < 0.001) with further adaptations noted at 8-weeks. HAd-ROM improved at a slower rate demonstrating significant changes only by 8 weeks.

**Conclusions:** Overhead, varsity-level athletes with an IR-ROM deficit ≥ 15°; benefit from performing an 8-week posterior shoulder stretch exercise. Benefits were noted through increased IR- and HAd-ROM as well as an increase in subjects’ self-reported shoulder functional ability.

# Abstract: A043 – INDOOR AND OUTDOOR WALKING PERFORMANCE AFTER TOTAL KNEE REPLACEMENT

**Published:** 2012-05

**Authors:** AST Storey, DM Brinkman, RA Bauck, AM Myrah, SN Friess, SC Webber

**Affiliations:** University of Saskatchewan, School of Physical Therapy, Saskatoon, Sask

**Purpose/Objectives and Rationale:** To compare indoor and outdoor walking performance in healthy older adults and in individuals after total knee replacement.

**Relevance to Physiotherapy Practice:** Walking impairments are common early after knee replacement, but few studies have investigated performance after healing is complete. In addition, walking is almost exclusively evaluated indoors and the relationship between indoor and outdoor performance is unknown.

**Materials and Methods:** Thirty-eight participants (22 controls, 16 with unilateral total knee replacements, 67.2 ± 5.6 years of age) completed indoor tests (6-Minute Walk, 10 m walk, Timed-Up-and-Go, stairs) and a 600m outdoor route (concrete, curbs, grass, ramp, stairs). We used accelerometers to measure step counts and global positioning system watches to measure distances and speeds outdoors.

**Analysis:** We used *t*-tests and Mann-Whitney Rank Sum tests to examine group differences and Pearson correlations to examine associations among walking tests.

**Results:** Participants with total knee replacements (22.9 ± 9.7 months post-surgery) demonstrated significantly reduced knee flexion (*p* < 0.001), lower Knee Outcomes Survey Activities of Daily Living scores (*p* = 0.03), greater perceived walking challenge (*p* = 0.04), and a greater number of self-reported health conditions (*p* = 0.03). However, there were no differences in walking performance between groups. Outdoor walking time was moderately correlated with the 6-Minute Walk, stair climb and descend, 10m walk and Timed-Up- and-Go (*r* values ranged from 0.65–0.79, *p* < 0.001).

**Conclusions:** Indoor and outdoor walking performance is not limited two years after total knee replacement, despite reduced knee flexion and greater self-reported functional limitations. In older adults simple indoor tests (10m walk, Timed-Up-and-Go) are moderately correlated with walking on different surfaces outdoors.

**Keywords:** mobility, older adults, arthroplasty, accelerometry, global positioning system

# Abstract: A044 – FACTORS THAT INFLUENCE PHYSICAL ACTIVITY IN INDIVIDUALS WITH RHEUMATOID ARTHRITIS – RESULTS OF A MAIL-OUT SURVEY

**Published:** 2012-05

**Authors:** A Taphorn

**Affiliations:** University of Saskatchewan Angela Busch, University of Saskatchewan, Saskatoon, Sask

**Purpose/Objectives and Rationale:** To investigate factors that influence levels of physical activity among individuals with rheumatoid arthritis, and if these factors differ with biologics as a pharmaceutical intervention.

**Relevance to Physiotherapy Practice:** Physical therapy programs that include interventions of aerobic exercise, strength training, and range of motion have been part of successful management in controlling rheumatoid arthritis symptoms.

**Materials and Methods:** A mail-out questionnaire was used to obtain self-reported measures of demographics, medication use, level of disability, pain, and barriers to physical activity. Patients were informed of the study by their rheumatologist and mailed the survey if interested.

**Analysis:** Chi-square tests, independent sample *t*-tests, and Pearson product moment correlations were used.

**Results:** Mean overall pain and mean pain upon walking, measured by the visual analog scale, were significantly lower for those not on biologics. Mean affect score, measured by the Arthritis Impact Measurement Scales 2 – Short Form, was significantly higher in those on biologics. A significant positive correlation between the inflamed joint score and overall pain and pain upon walking was found. There was a negative correlation between discouragement, poor health, and fatigue and physical activity measured using the International Physical Activity Questionnaire – Long Form.

**Conclusions:** There were no difference in amount of physical activity between individuals on biologics and those not on biologics. Barriers and characteristics that predict levels of physical activity were identified to be pain, lack of facility, low self-reported health status, level of joint damage, self-reported fatigue, poor health, and discouragement. These factors should be considered in when physical activity is incorporated into an individual’s management strategy.

**Keywords:** rheumatoid arthritis, physical activity

# Abstract: A048 – STRENGTH TRAINING THE NON-FRACTURED LIMB IMPROVES STRENGTH AND RANGE OF MOTION IN THE FRACTURED LIMB AFTER WRIST FRACTURES

**Published:** 2012-05

**Authors:** CRA Magnus, CM Arnold, G Johnston, Haas V Dal-Bello, J Basran, JR Krentz, JP Farthing

**Affiliations:** College of Kinesiology, University of Saskatchewan; School of Physical Therapy, University of Saskatchewan; Department of Surgery, College of Medicine, University of Saskatchewan; School of Rehabilitation Science, McMaster University; Division of Geriatric Medicine, College of Medicine, University of Saskatchewan

**Purpose/Objectives and Rationale:** The purpose was to apply cross-education (i.e., cross-training) during recovery from wrist fractures in women (> 50 years) and evaluate effects on strength and range of motion (ROM).

**Relevance to Physiotherapy Practice:** Cross-education helps maintain strength in an opposite immobilized limb in healthy individuals. Whether this effect can be applied during recovery from unilateral injuries is unknown.

**Materials and Methods:** Eighteen women were randomized to normal rehabilitation (NORM), or normal rehabilitation plus strength- training (NORM+TRAIN). Normal rehabilitation consisted of forearm casting for 40.8 (SD 6.2) days, and hand exercises. Non- fractured hand strength-training for NORM+TRAIN was completed at home using a handgrip trainer. Strength-training began immediately after the first visit (week 1). Force via a handgrip dynamometer and ROM (flexion/extension; supination/pronation) via a goniometer were measured at weeks 1 (non-fractured hand), 9 and 12 (both hands).

**Analysis:** Strength of the non-fractured limb was analyzed using Repeated Measures ANOVA. One-way ANOVA was used to analyze percent change in strength of the fractured limb, and ROM.

**Results:** The non-fractured hand of NORM+TRAIN increased strength from week 1 [25.5 (SD 6.8)] to weeks 9 [27.7(SD 7.8)], and 12 [28.3 (SD 8.0)]; *p* < 0.05. NORM+TRAIN had a less deficit in strength [-40.8% (SD 21.4)] at 12-weeks compared to NORM [-65.1% (SD 21.7)]; *p* < 0.05. For ROM at 12-weeks post fracture, NORM+TRAIN improved wrist flexion [45° (SD9.8)], and extension [52° (SD 12.3)] compared to NORM [33.5° (SD 11.5)] [34° (SD 19.9)], respectively; *p* < 0.05.

**Conclusions:** Strength training the non-fractured limb improved strength and ROM in the fractured limb at 12-weeks post-fracture. This may have implications for rehabilitation following wrist fractures.

**Keywords:** cross-education, rehabilitation, distal radius

# Abstract: A050 – IMPACT OF INITIAL SYMPTOM BURDEN ON TIME TO RETURN TO ACTIVITY AFTER A CONCUSSION IN CHILDREN AND ADOLESCENTS

**Published:** 2012-05

**Authors:** D Berardi, M Brizard, H Brière, M Charron, I Gagnon

**Affiliations:** School of Physical and Occupational Therapy, McGill University, Montreal, Que

**Purpose/Objectives and Rationale:** Document the time to return to activity and post-concussion symptoms in a group of children with concussion who presented with specific symptoms at the time of injury and compare those to children who presented without those initial symptoms.

**Relevance to Physiotherapy Practice:** There remains a lack of consensus on appropriate treatment and return to play protocols following concussion in children. It would be helpful to determine whether specific symptoms at the time of injury could be indicators of longer recovery.

**Materials and Methods:** 42 children, aged between 6 and 17 years old, who sustained a concussion were recruited. The presence and severity of post- concussion symptoms were assessed at 2 and 12 weeks post-injury using the Post-Concussion Symptom Scale. Initial symptoms were recorded at the time of the Emergency Department visit.

**Analysis:** Independent sample *t*-tests were performed to compare the time to return to play, as well as post-concussion symptoms at 2 weeks and 3 months post-injury in both groups.

**Results:** Children presenting with an initial headache took longer to return to play (*p* = 0.001). Children who did not vomit at the time of injury took longer to return to play than those who did (*p* = 0.001). Presence of headache at the time of injury resulted in higher cognitive post-concussion symptoms scores at visit 2 weeks post-injury (*p* = 0.04).

**Conclusions:** The presence of headache and the absence of vomiting as initial symptoms may help physiotherapists and other health care professionals in providing additional supervision and in setting realistic expectations for a safe return to play in children and adolescents.

**Keywords:** concussion, mild traumatic brain injury, pediatrics, physical activity, post-concussion symptoms

# Abstract: A052 – ACUPUNCTURE AND SYMPTOM MANAGEMENT IN CANCER PATIENTS

**Published:** 2012-05

**Authors:** T Tran, G Kasymjanova, M Grossman, T Xenopoulos, T Jagoe, J Agulnik, D Small

**Affiliations:** Peter Brojde Lung Cancer Center, Jewish General Hospital, Montreal, Que

**Purpose/Objectives and Rationale:** Cancer patients experience treatment and disease-related symptoms which contribute to high levels of psychological distress and poor quality-of-life. We explored the potential effect of acupuncture on symptoms and well-being in cancer patients.

**Relevance to Physiotherapy Practice:** There is evidence that acupuncture is a safe and effective therapy that may reduce pain, nausea, depression, anxiety and neuropathy.

**Materials and Methods:** Prospectively collected quality of life data from 21 symptomatic cancer patients treated with acupuncture was analyzed. Edmonton Symptom Assessment Scale (ESAS) was used to measure 13 different symptoms and 1 measure of well- being. Acupuncture (laser and/or dry needles) treatment was given 1-2 sessions/wk for a minimum of 6 weeks. ESAS was completed at baseline and end of treatment.

**Analysis:** 21 pts (11M and 10F), mean age 60 (range 36–85), received an average 11 sessions/pt (range 4–26). Ten patients had ECOG PS of 1; 11 patients had PS ≥ 2. Ten patients were on chemotherapy. *T*-test & Pearson correlation statistics were utilized for analysis.

**Results:** After acupuncture, pain significantly improved from 5.2 to 2.9 (*p* < 0.001), nervousness from 4.0 to 2.3 (*p* = 0.012), depression from 3.1 to 2.0 (*p* = 0.040), and perceived well-being from 4.6 to 2.5 (*p* = 0.003). Half the advanced stage pts were not on narcotics at end of treatment.

**Conclusions:** This is the first review looking at the effect of acupuncture primarily in advanced lung cancer patients. Acupuncture seems to be a feasible modality in reducing symptoms related to treatment or cancer itself and promote well-being. No complications were reported during the acupuncture. Further exploration of these findings in randomized controlled trial is recommended.

**Keywords:** acupuncture, cancer, well-being

# Abstract: A053 – THE BARRIERS AND FACILITATORS OF HIP PROTECTOR USE: PERSPECTIVES OF FAMILY MEMBERS, NURSES AND COMPANIONS

**Published:** 2012-05

**Authors:** C Kinlin, T Marlow, LA Donald, S Tiessen, N Cooper, S Pryse-Phillips, K Yoshida

**Affiliations:** University of Toronto; Department of Physiotherapy, Sunnybrook Health Sciences Centre, Toronto, Ont

**Purpose/Objectives and Rationale:** To identify the facilitators and barriers of hip protector use in the elderly population from the perspective of family members, nurses, and companions.

**Relevance to Physiotherapy Practice:** Hip fractures are a serious problem for elderly residents living in long term care facilities. Hip protectors are used to prevent hip fractures; however, compliance is a limiting factor. It is unclear what factors prevent or promote hip protector use.

**Materials and Methods:** Data were collected from focus groups and interviews occurring from February to April 2011. Participants were recruited using convenience sampling of family members, nurses and companions at Sunnybrook Health Sciences Centre’s Veterans Centre in Toronto, Canada. Five focus groups were conducted, two for family members (*n* = 10, *n* = 9), two for nurses (*n* = 8, *n* = 7), and one combined for a companion and nursing students (*n* = 3). One interview was also conducted with a companion.

**Analysis:** Themes were developed from field notes and verbatim transcripts using descriptive qualitative analysis (1).

**Results:** Three main themes affecting compliance were identified: 1) transfer of knowledge and the role of beneficence, 2) physical characteristics and 3) access to product. These themes were evident to varying degrees in all three populations.

**Conclusions:** We conclude that there is need for changes in design, additional education regarding efficacy and access, and an increase in the number of hip protectors provided to each resident. (1) Krueger R. Focus groups: a practical guide for applied research. 3rd ed. ed. Thousand Oaks, Calif.: Sage Publications; 2000.

**Keywords:** hip protectors, hip fractures, compliance, long term care facilities, Veterans Affairs Canada

# Abstract: A055 – CHILDREN’S AND PARENTS’ BELIEFS REGARDING THE VALUE OF WALKING: IMPLICATIONS FOR REHABILITATION WITH CHILDREN WITH CEREBRAL PALSY

**Published:** 2012-05

**Authors:** G Teachman, V Wright, D Fehlings, N Young, P McKeever

**Affiliations:** * Holland Bloorview Kids Rehabilitation Hospital, Toronto; † Laurentian University, Sudbury, Ont

**Purpose/Objectives and Rationale:** The aims of this pilot study were:

1. to describe beliefs about the value of walking held by children with CP and their parents, and,
2. to examine how beliefs about the value of walking inform therapy choices and perceptions of ’success.’

**Relevance to Physiotherapy Practice:** There is much debate in physiotherapy regarding the extent to which time and energy should be spent on promoting ambulation for children with cerebral palsy (CP) versus alternative modes of mobility. These debates are inherently value laden and centre on ethical questions on what is “best” for children.

**Materials and Methods:** A conceptually-grounded qualitative design was employed. Six parents and six children with CP each participated in a one-to-one qualitative interview.

**Analysis:** Analysis examined the relationship between dominant social beliefs regarding walking and disability, and participants’

contextualized responses.

**Results:** Four themes were developed:

1. Parents and children engage in multiple strategies to minimize negative bodily capital (“stigma”).
2. Walking was seen as an end in itself whether or not it was functional.
3. ’Good’ parents were perceived as those who ’try everything’.
4. Children internalize dominant discourses of disability and judge themselves and others accordingly.

**Conclusions:** These pilot results begin to reveal how dominant beliefs about disability shape parents’ and kids’ choices and contributing to feelings of angst, guilt and doubt for parents and negative self-image with children. We suggest that ’family centred care’ needs to extend beyond soliciting family preferences to engaging families in ongoing discussion of their evolving values and balancing treatment priorities with other developmental aims.

**Keywords:** ambulation, cerebral palsy, children, parents, qualitative research, values

# Abstract: A056 – A PILOT SENSITIVITY TO CHANGE STUDY OF REHABILITATIVE ULTRASOUND IMAGING OF THE TRANSVERSUS ABDOMINIS AND LUMBAR MULTIFIDUS IN PATIENTS WITH LOW BACK PAIN

**Published:** 2012-05

**Authors:** EC Parent, R Breitkreitz, J Ladd, K McIntosh, D Pauls, S Urhbach, S Koppenhaver

**Affiliations:** * Dept. Phys. Ther. University of Alberta, Edmonton, Alta.; † US Army Baylor University Doctoral Physical Therapy Program, San Antonio, Texas, USA

**Purpose/Objectives and Rationale:** The reliability and validity of muscle thickness measurements using rehabilitative ultrasound imaging (RUSI) is good. However, little research reports on its sensitivity-to-change. The goal of this study was to quantify the sensitivity-to-change in thickness measurements of the transversus abdominis (TrA) and lumbar multifidus (LM) using RUSI in patients with low back pain (LBP).

**Relevance to Physiotherapy Practice:** Sensitivity to change evidence is needed to support using RUSI to detect changes in core muscles in research and justify RUSI use clinically.

**Materials and Methods:** Six subjects with LBP predicted to not fail a stabilization program completed a six-week specific motor control exercise program. RUSI images were taken of bilateral TrA and LM at rest and during submaximal contractions in random orders at baseline, 3 and 6 weeks. Muscle thicknesses were measured with evaluators blind to the IDs or evaluation time.

**Analysis:** Standardized response means (SRM) were calculated for three time intervals for the change in the measurement of each muscle at rest, during contraction and for the difference between the contracted and rest thickness. Reliability was assessed using Intraclass Correlation Coefficients (ICC).

**Results:** Standardized response means ranged between -0.76 and 0.61 indicating RUSI can be moderately sensitive to both positive and negative changes. Although differences between measurements were not statistically significant, point estimates suggested RUSI had the greatest sensitivity to detect changes in muscle activation. Inter-rater reliability was good with ICC’s ranging between 0.84 and 1.00.

**Conclusions:** In this pilot study, RUSI appeared adequately sensitive to positive and negative changes over short and long periods of stabilization training.

**Keywords:** stabilization, diagnostic ultrasound, psychometric properties, core muscles, reliability

# Abstract: A057 – REPEATED MOVEMENT TESTING IN PATIENTS WITH LOW BACK PAIN (LBP) USING THE MECHANICAL DIAGNOSIS AND THERAPY (MDT) APPROACH: HOW MUCH IS ENOUGH?

**Published:** 2012-05

**Authors:** EC Parent, C Alderdice, J Evans, S Feldman, S Robinson, L White, A Long, M McDougall, F Bonnet, C Brososky

**Affiliations:** * Dept. Phys. Ther. University of Alberta, Edmonton; † Bonavista Physical Therapy, Calgary; ‡ West End Physiotherapy, Edmonton, Alta

**Purpose/Objectives and Rationale:** The variability in the reported prevalence and reliability estimates of MDT findings may be due to testing more or less repeated movements. Our goal was to determine the number of repeated movements needed to maximize the reliability and the detection of directional preference (DP) in patients with low back pain (LBP).

**Relevance to Physiotherapy Practice:** The MDT approach is widely used by physiotherapists and effective for LBP.

**Materials and Methods:** Up to 40 repeated movements were videotaped for 31 patients with LBP. Patients reported symptoms after completing 5, 10, 20, 40 repetitions in six directions. Four raters recorded the symptoms response, and directional preference (DP) using edited videos of each set.

**Analysis:** We assessed interrater reliability with AC1 coefficients and compared the prevalence of ratings between sets using chi-square.

**Results:** The interrater reliability of symptoms ratings reached our target (> 0.61) for all sets except for one movement but that of DP only achieved our target after completing 40 reps. No statistically significant difference in reliability were observed between sets. There were no statistically significant differences between any sets for the prevalence of DP and for all except one movement for symptom ratings. Clinically important differences (> 33%) were detected between all subsequent sets for the prevalence of DP and for all movements up to 10 reps and for some movements until 40 reps for prevalence of symptoms ratings.

**Conclusions:** The optimal number of repetitions may be between 10 and 40. Further research will help formulate a recommendation to maximize reliability and obtain stable prevalence estimates. Testing only 5 repetitions is insufficient.

**Keywords:** low back pain, directional preference, repeated movement testing, reliability, assessments, exercises

# Abstract: A058 – “MY BODY’S A 50-YEAR-OLD BUT MY BRAIN IS DEFINITELY AN 85-YEAR-OLD": EXPLORING THE EXPERIENCES OF MEN AGING WITH HIV-ASSOCIATED NEUROCOGNITIVE CHALLENGES

**Published:** 2012-05

**Authors:** L Hopcroft, L Bester, D Clement, A Quigley, M Sachdeva, S Rourke, S Nixon

**Affiliations:** * Department of Physical Therapy; ‡ International Centre for Disability and Rehabilitation, University of Toronto; † St. Michael’s Hospital, Toronto, Ont

**Purpose/Objectives and Rationale:** To explore the experiences of men aging with HIV-associated neurocognitive challenges using the Episodic Disability Framework.

**Relevance to Physiotherapy Practice:** HIV-associated neurocognitive challenges are identified as a pressing concern for people living with HIV in Canada. This disorder has been studied from a neuropsychometric perspective, but little attention has been paid to people’s perceptions of how these challenges impact their lives.

**Materials and Methods:** This interpretive, qualitative study involved in-depth, semi-structured, one-on-one interviews with twelve men 50 years and older who self-identified as having HIV-associated neurocognitive challenges. Participants were recruited through a Neurobehavioural Research Unit. The interview guide was developed based on the Episodic Disability Framework.

**Analysis:** Data were analyzed thematically following an open coding technique.

**Results:** Participants’ experiences reflected all concepts within the Episodic Disability Framework. Memory and attention were reported as the most taxing neurocognitive impairments. Difficulties with daily activities were variable and specific to individual participants, but similar living strategies were employed. Most men reported being unclear about the link between their neurocognitive challenges and aging. Others discussed aging as an asset that helped with coping.

**Conclusions:** This is the first qualitative study to use a disability framework to examine the experiences of men aging with HIV- associated neurocognitive challenges. It reframes the episodic disability experienced by these individuals as predictably linked to certain triggers. As such, support for managing neurocognitive challenges could focus on triggers that exacerbate the condition as opposed to the impairments themselves.

**Keywords:** aging, chronic disease, disability, HIV, neurocognitive challenges, qualitative methods

# Abstract: A059 – STATIN-INDUCED RHABDOMYOLYSIS: A REVIEW OF 104 CASE REPORTS

**Published:** 2012-05

**Authors:** P Mendes, P Robles, S Mathur

**Affiliations:** Dept. of Physical Therapy, University of Toronto, Toronto, Ont

**Purpose/Objectives and Rationale:** Rhabdomyolysis is a potentially lethal clinical syndrome that results from acute muscle damage. There is a link between rhabdomyolysis and the use of statin medications. The purpose of this study was to identify common pre-disposing factors associated with statin-induced rhabdomyolysis.

**Relevance to Physiotherapy Practice:** As the use of statin therapy for dyslipidemia increases in the population, there is a greater risk for statin-induced rhabdomyolysis. Physiotherapists need to be aware of the risk for rhabdomyolysis in their clients, as they may be the first to identify symptoms of this condition.

**Materials and Methods:** We conducted a systematic review (1990–2011) to identify case reports of statin-induced rhabdomyolysis referenced in MEDLINE¯, CINAHL, Scopus and PEDro. Keywords included: rhabdomyolysis, statins, myopathy, myalgia, muscle damage and muscle injury.

**Analysis:** Data were extracted from the case studies and described using means, standard deviation (SD) and frequencies.

**Results:** A total of 104 cases were included: 71 males, 33 females, age: 62.3 ± 14.6 years. Mortality was 13.4% within these patients. Simvastatin was the most commonly reported statin (*n* = 49) followed by Atorvastatin (*n* = 19). In 96% cases, Simvastatin was being administered in conjunction with another medication, such as fibrates (*n* = 11). Muscle pain (*n* = 59) and muscle weakness (*n* = 60) were common symptoms of rhabdomyolysis, however referral to rehabilitation was only indicated in 20 cases and no description of rehabilitation was provided.

**Conclusions:** Statin-induced rhabdomyolysis was more commonly reported when statins were used in conjunction with other drugs. Future studies should address the role of physiotherapy in restoring muscle function following rhabdomyolysis.

**Keywords:** rhabdomyolysis, muscle damage, myopathy

# Abstract: A013 – ADVANCED CLINICIAN PRACTITIONER IN ARTHRITIS CARE (ACPAC) STAKEHOLDER REVIEW: GRADUATE AND COLLEAGUE PERCEPTIONS OF CURRENT ROLE UTILIZATION AND SUGGESTIONS FOR FUTURE ACTION

**Published:** 2012-05

**Authors:** K Warmington, C Kennedy, K Lundon, L Rozmovits, S Lineker, R Shupak, R Schneider

**Affiliations:** * St. Michael’s Hospital; † Linda Rozmovits Qualitative Research; ‡ The Arthritis Society; § The Hospital for Sick Children, Toronto, Ont

**Purpose/Objectives and Rationale:** The purpose of this qualitative study is to describe current role utilization and role satisfaction among Advanced Clinician Practitioner in Arthritis Care (ACPAC) Program graduates, referred to as Extended Role Practitioners (ERPs), and their colleagues, and to describe future possibilities for ERPs by detailing participants’ suggestions for improving deployment and facing system-level challenges.

**Relevance to Physiotherapy Practice:** Stakeholder perspectives on ERP roles provide many valuable insights including the relevant challenges, far-reaching effects and most feasible opportunities for change and growth.

**Materials and Methods:** Three focus groups (*n* = 20 graduates) and 18 individual interviews (administrative and clinical colleagues) were conducted, digitally audio-recorded and transcribed.

**Analysis:** Transcripts were coded for anticipated and emergent themes using the method of constant comparison with searched for disconfirming evidence.

**Results:** Four common topic areas emerged among ERPs and colleagues: i) values and motivations were expressed as reasons for pursing or participating in the ACPAC program; ii) skills and expertise were addressed through perceptions of improved clinical competence; iii) relationships were examined through ERPs’ impact on patients and; iv) current role utilization highlighted role variations and barriers to achieving full utilization of extended competencies. Colleagues of ERPs raised a fifth topic: v) future action, including optimizing deployment and confronting system-level challenges.

**Conclusions:** There are many perceived benefits of the ACPAC program training and the resulting extended practice roles. Current role utilization is highly varied and role satisfaction, though generally high, is heavily impacted by unit, institution and system-level barriers and facilitators. Suggestions to improve deployment must be mobilized for the benefit of patients with arthritis.

**Keywords:** extended scope practice, arthritis, continuing education, role satisfaction, qualitative

# Abstract: A014 – PATIENT SATISFACTION WITH ACPAC PROGRAM TRAINED EXTENDED ROLE PRACTITIONERS: A MULTI- CENTRE STUDY

**Published:** 2012-05

**Authors:** CA Kennedy, K Warmington,, S Lineker, L Soever, L Passalent, Lundon K Katie, R Shupak, R Schneider

**Affiliations:** St. Michael’s Hospital, Martin Family Centre for Arthritis Care & Research and the Mobility Clinical Research Unit; The Arthritis Society, Ontario Division; Mount Sinai Hospital; University Health Network, Toronto Western Hospital; Hospital for Sick Children, Toronto, Ont

**Purpose/Objectives and Rationale:** Advanced Clinician Practitioner in Arthritis Care (ACPAC) program graduates are generating and integrating new models of arthritis care into the Ontario health care system. This study evaluates patients’ satisfaction with the care received from program graduates, referred to as Extended Role Practitioners (ERPs).

**Relevance to Physiotherapy Practice:** It is essential to capture patients’ perceptions of the care received from this new human health resource.

**Materials and Methods:** Cross-sectional, self-report study of 325 patients from 27 ERPs recruited from 15 health care institutions across Ontario. The Patient-Doctor Interaction Scale, reflecting patient-therapist interaction (PTIS) was the primary outcome measure [subscales: Providing Information (PI), Rapport (R), Meeting Patient Needs (PN)]. Secondary outcomes included satisfaction with: services received, wait times, and ERP-based care compared to that received from other health care providers.

**Analysis:** Analyses included descriptive statistics and qualitative feedback.

**Results:** Respondent demographics: mean age 54 years (3–75), female (72%), adult (82%), living in urban area (79%). Scores reported as mean (1-5 = very satisfied) or percent response. PTIS scores were high; PI: 4.5, R: 4.6, PN: 4.6. Satisfaction with services received: items ranged from 4.1–4.6. Agreement that wait time was acceptable: from referral (88%), in clinic (87%). Most felt ERP-based arthritis care was comparable to (37%) or better than (61%) that provided by other health care professionals.

**Conclusions:** Given patients’ consistent satisfaction across outcomes, the growth and development of ACPAC ERP-based models of care and the surrounding policy implications should be explored.

**Keywords:** extended role practice, patient satisfaction, arthritis care

# Abstract: A026 – REHABILITATION PROFESSIONALS’ PERSPECTIVES ON AND PARTICIPATION IN PRIMARY HEALTH CARE IN RURAL BRITISH COLUMBIA

**Published:** 2012-05

**Authors:** RK Roots, L Bainbridge, H Brown, LC Li

**Affiliations:** * Dep’t of Physical Therapy; † College of Health Disciplines; ‡ School of Nursing, University of British Columbia; § Arthritis Research Centre of Canada, Vancouver, B.C.

**Purpose/Objectives and Rationale:** To examine physiotherapists’ (PTs) and occupational therapists’ (OTs) understanding of primary health care (PHC) and their perceived role in PHC in rural British Columbia (BC).

**Relevance to Physiotherapy Practice:** Understanding how PTs and OTs are currently engaged in PHC and the barriers to participation will inform efforts to integrate rehabilitation services into rural PHC.

**Materials and Methods:** A purposive sample of PTs and OTs was recruited by mail to rural workplaces, and selected according to work experiences, roles and practice settings. Through interviews, participants were asked about their understanding of and involvement in PHC, the challenges they faced, and mitigating strategies.

**Analysis:** Guided by Interpretive Description, transcripts were analysed inductively categorizing experiences and perceptions, and interpreting findings for education, practice, and policy implications.

**Results:** From interviews with 13 PTs and 6 OTs, participants described rural practice as consistent with a PHC approach (addressing determinants of health and health promotion) however many could not define PHC and only one participant was formally engaged in a PHC model. While interprofessional (IP) care was a strategy to overcome resource shortages, a number of barriers were encountered at the provider level: poor understanding of PT/OT roles, professional cultures, and insufficient resources; and at the systems level: financial models, referral policies, and communication channels.

**Conclusions:** Despite rural rehabilitation practice aligning with the tenets of PHC, barriers limit the engagement of PTs and OTs in PHC. Our findings lend support for IP education to address provider issues and reviewing funding models and policies at the systems level to facilitate integrating rehabilitation services into rural PHC.

**Keywords:** interprofessional education, primary health care, rural health, rural practice

# Abstract: A027 – IMPROVING RURAL ACCESS TO CARE: PATIENT PERSPECTIVES ON AN ADVANCE PRACTICE PHYSIOTHERAPY MODEL

**Published:** 2012-05

**Authors:** K Gillis, A Augruso, T Coe, A O’Neill, L Radford, BE Gibson, L Soever, L O’Callaghan

**Affiliations:** University of Toronto Department of Physical Therapy; Mt. Sinai Hospital, Toronto, Ont

**Purpose/Objectives and Rationale:** The purpose of this study was to explore perspectives of individuals with hip/knee arthritis regarding an advanced practice physiotherapy (APP) model of care in a rural setting.

**Relevance to Physiotherapy Practice:** APP models of care have been shown to reduce wait times and increase patient satisfaction. However, there are no Canadian studies that have investigated patient perspectives regarding the use of an APP model of care in rural settings.

**Materials and Methods:** A qualitative study design with semi-structured interviews was utilized to ascertain the perspectives of patients who had experienced an APP model of care. Each interview was audiotaped, transcribed verbatim, and uploaded into a qualitative software program.

**Analysis:** Content analysis was conducted on the transcripts by coding line by line to identify emergent themes.

**Results:** Three main themes emerged from the data; “timely access to care made a difference”; “distance was an important factor in seeking care”; and “the APP model of care was positively perceived”. Participants chose the APP model of care because it provided shorter wait times for care (including total joint replacement for hip and knee arthritis) compared to traditional models of care. Participants enjoyed having the majority of their care in their community and they valued the amount of time the APP was able to spend with them.

**Conclusions:** Participants perceived that the APP model of care provided a viable alternative for care of their hip/knee arthritis. In future, similar programs have potential to reduce wait times and increase access to care for rural communities.

**Keywords:** wait times, patient perspectives, rural, arthritis, advance practice physiotherapy

# Abstract: A033 – SATISFACTION OF PARTICIPANTS AND REFERRING HEALTH CARE PROVIDERS WITH A SPINAL TRIAGE ASSESSMENT SERVICE DELIVERED BY PHYSIOTHERAPISTS IN COLLABORATION WITH ORTHOPAEDIC SURGEONS

**Published:** 2012-05

**Authors:** B Bath, B Janzen

**Affiliations:** * School of Physical Therapy; † Community Health and Epidemiology, College of Medicine, University of Saskatchewan, Saskatoon, Sask

**Purpose/Objectives and Rationale:** To evaluate participant and referring care provider satisfaction with a spinal triage assessment service delivered by physiotherapists.

**Relevance to Physiotherapy Practice:** Evaluating the satisfaction of patients and referring care providers is an important outcome as the perceptions of both groups are crucial to the acceptance and adoption of this new and emerging role for physiotherapists.

**Materials and Methods:** Participants (*n* = 115) were recruited from those referred to the spinal triage assessment program. Measures of patient and referring provider satisfaction were completed at 4 weeks after the assessment.

**Analysis:** Descriptive statistics and qualitative thematic analysis of comments were used.

**Results:** Ninety-four percent of the participants completed the satisfaction survey: 66% were “very satisfied with the service and 55% were “very satisfied” with the recommendations. Only 18% of referring care providers completed the satisfaction survey and 90.5% of those were “very satisfied” with the recommendations. Sixty-one participants and 14 care providers provided comments with a diverse range of themes which were coded into positive, negative and neutral related to the triage service and an “other” category unrelated to the service.

**Conclusions:** There were high levels of participant satisfaction with the service and slightly less satisfaction with the recommendations. Satisfaction of referring care providers with the recommendations and report was also high, but given the low response rate, these results should be interpreted with caution. Qualitative analysis of participant and provider comments revealed a diverse range of themes. The “other” issues identified may be important contextual factors that have the potential to impact patient relevant outcomes.

**Keywords:** interprofessional practice, quality assurance, back pain, orthopaedics

# Abstract: A034 – DIAGNOSTIC AND MANAGEMENT RECOMMENDATION CONCORDANCE BETWEEN PHYSIOTHERAPISTS AND AN ORTHOPAEDIC SURGEON FOR LOW BACK COMPLAINTS

**Published:** 2012-05

**Authors:** B Bath, Grona S Lovo, R Bourassa, J Reilly, B Janzen

**Affiliations:** * School of Physical Therapy; † Department of Surgery; ‡ Community Health and Epidemiology, College of Medicine, University of Saskatchewan, Saskatoon, Sask

**Purpose/Objectives and Rationale:** To determine the diagnostic and management recommendation concordance between physiotherapists (PTs) practising in a collaborative reasoning model, a solo PT and an orthopaedic surgeon for people with low back complaints presenting to a spinal triage assessment program delivered by PTs.

**Relevance to Physiotherapy Practice:** With shifting professional boundaries, where a PT is the first point of contact for orthopaedic surgical referrals, ensuring a high level of clinical reasoning is paramount.

**Materials and Methods:** Assessments were performed by a PT with advanced orthopaedic training. Diagnosis and management recommendations were determined jointly between the assessing PT and a consultant PT. Forty-five de-identified assessment reports (with diagnosis and management recommendations removed) were reviewed by an orthopaedic surgeon and another PT not involved in the assessment who each completed a clinical and management classification tool.

**Analysis:** Differences were evaluated with chi-square and Fisher’s exact tests.

**Results:** There were no significant differences in diagnosis or referral for advanced imaging (i.e., CT or MRI) between provider groups (*p* > 0.05). Significant differences in management recommendations were: “PT + PT consultant” more likely than other groups to recommend “urgent referral to surgeon” (*p* = 0.014) and “PT only” more likely to refer for PT treatment (*p* < 0.001) and recommend X-rays (*p* = 0.024).

**Conclusions:** PTs and an orthopaedic surgeon appear to make similar diagnoses for people with low back-related problems. PTs working in a collaborative reasoning model (i.e., PT + PT consultant) appear to make more similar management recommendations to surgeons than a PT not working in a collaborative model.

**Keywords:** orthopedic, collaborative reasoning, spinal triage, clinical reasoning, surgical referral

# Abstract: A042 – EVENING PHYSIOTHERAPY SERVICE: WHAT DIFFERENCE DOES IT MAKE?

**Published:** 2012-05

**Authors:** M Prendergast, M Derbyshire, CM Anderson

**Affiliations:** Physiotherapy Service, London Health Sciences Centre, London, Ont

**Purpose/Objectives and Rationale:** To evaluate an innovative evening physiotherapy service (EPS) at an Acute Care Teaching Hospital in Ontario.

**Relevance to Physiotherapy Practice:** An EPS was developed by an interprofessional team to provide timely access to physiotherapy services outside regular hours. Program evaluation is essential to ensure that we effectively manage scarce health care resources whilst optimizing patient care.

**Materials and Methods:** Workload measurement data was reviewed to identify utilization and productivity for the service. Evening service records were examined for the frequency of each priority category for all units from June 2010 to May 2011. A survey was developed, piloted and modified which examined experiences of physiotherapy staff and key stakeholders with the highest EPS utilization.

**Analysis:** Descriptive statistics were calculated for numeric data where applicable, and counts were made for categorical data.

**Results:** Over 750 patients received EPS which was offered Monday to Friday until 2300. The top three clinical units utilizing the service were: Intensive Care (34%), Thoracic Surgery (20%) and Respirology (12%). The demand for the service had increased by 20% over the past three years. Referrals were primarily for: significant cardiorespiratory compromise, late referrals, multiple treatments required and complex discharge planning. Positive experiences with the service were reported by Physiotherapists providing EPS, and those requesting it for their patients.

**Conclusions:** This innovative service realigned resources from ’regular hours’ to provide timely access to care for those patients most in need of the expertise of a Physiotherapist. The EPS is strongly supported by the interdisciplinary teams and is cost effective.

**Keywords:** evening physiotherapy service, program evaluation

# Abstract: A049 – CONCUSSION EDUCATION IN PEDIATRIC SPORTS: USING THE MONTREAL CHILDREN’S HOSPITAL CONCUSSION KIT AS A TOOL FOR KNOWLEDGE TRANSLATION

**Published:** 2012-05

**Authors:** D Friedman, I Gagnon

**Affiliations:** Montreal Children’s Hospital Trauma Programs, Montreal, Que

**Purpose/Objectives and Rationale:** The diagnosis and management of concussions, also known as mild traumatic brain injuries, in children and adolescents poses a challenge for clinicians, parents, coaches, and teachers. The Montreal Children’s Hospital (MCH) recently released the 2nd Edition of the Concussion KiT to increase awareness with respect to preventing, recognizing and managing concussions in pediatric sports. The purpose of this paper is to review the development and implementation of this tool and to explore how physiotherapists can use such tools for community outreach and education.

**Relevance to Physiotherapy Practice:** Physiotherapists are often involved with pediatric elite sports participants where concussions are common occurrences. However, little supervision is offered to children in recreational-level sports and physiotherapists have a responsibility to educate coaches, parents and children themselves to ensure proper recognition, management and safe return to physical activities.

**Materials and Methods:** Following an exhaustive literature review as well as a consultation with key stakeholders, the MCH Trauma Program Interprofessional Team, led by physiotherapists, developed a comprehensive bilingual multi-component knowledge translation package, the Concussion KiT. Coaches receiving the Concussion KiT also receive a questionnaire to explore how successful the KiT is in changing their knowledge, attitudes, and practices related to the recognition and management of concussions.

**Analysis:** Qualitative analysis was performed on data from the questionnaires.

**Results:** Coaches report high satisfaction rates with the KiT and referrals to the MCH Concussion Clinic from teams exposed to the content of the KiT are timely and appropriate.

**Conclusions:** The KiT provides physiotherapists with an opportunity to combine knowledge in trauma and injury prevention and apply it to community knowledge translation in a proactive and innovative manner.

**Keywords:** concussion, mild traumatic brain injury, pediatric, knowledge translation, sport

# Abstract: P001 – ASSESSMENT OF THE PERSON WITH PERSISTENT POST-TRAUMATIC NECK PAIN: IS DELAYED RECOVERY ONLY DUE TO PSYCHO-SOCIAL PROBLEMS?

**Published:** 2012-05

**Authors:** G Gilbert, D Lazowski

**Learning Objectives and Session Content:** This inter-active workshop will present an assessment protocol appropriate for use with people with persistent post-traumatic neck pain. Participants will be provided with outcome measures, detailed history and assessment forms that include some questions and checklists to document 1) pre-accident status 2) events surrounding the trauma, and 2) post trauma management and documentation (i.e. immediate, ambulance, emergency department) Upon completion of the session, participants will:

1. Be able to distinguish the factors and / or characteristics of clients with persistent post-traumatic neck pain and emotional or psychological sequelae of the trauma vs. those who have indications of the possibility of another physical co-morbidity.
2. Be able to document the medical history, the chronology of events and ongoing symptoms to establish whether, in addition to the spinal and musculo-skeletal trauma, a head injury may have occurred (often referred to as a mild Traumatic Brain Injury (mTBI) or diffuse axonal injury).
3. Be provided with resources including clinic-ready outcome measures and practical assessment tools and have some experience using them.

**Relevance to Physiotherapy Profession:** Although most people recover well with an appropriate physiotherapy program, at some point in their practice, all physiotherapists encounter the small percentage of people with post-trauma neck injuries who continue to have persistent pain limiting their ability to return to all their pre-accident activities. Many of these individuals are otherwise healthy, happy, have a supportive family and work environment, are good problem solvers and have good adaptive coping skills. This presentation will give physiotherapists an alternative approach to assist those who fall outside the scope of routine clinical practice.

**Target Population:** Private practice, brain injury programs, vestibular therapy, neurology, complex care, mental health services, specialized geriatric services, long-term care and home care.

**Description of Supporting Evidence:** ’Pain Management’ has become an area of discussion and publication by many different health professionals and providers. However, it is usually discussed in a generic language-suggesting that ’one approach will work for everyone’. Too often, the client is presented with a battery of ’treatment options or tools’ with little or no understanding of how to implement their program. The International Association for the Study of Pain (IASP) defines pain as “An unpleasant sensory and emotional experience associated with actual or potential tissue damage or described in terms of such damage". Pain is usually deemed ’chronic’ or ’persistent’ when ’ persisting beyond the expected healing time of an injury or illness, usually considered beyond 6 months (though 12 weeks is often used as a red flag when treating people post trauma. The reality for the clinician, and the client, is that treating persistent post-traumatic neck pain is often very challenging. A large body of evidence supports the association of persistent pain and a lack of success with rehabilitation (including achievement of functional work and personal goals) with psychological and psycho-social factors. However, once these issues have been addressed, physiotherapists have few resources and strategies to continue helping people with persistent pain problems to resume socio-occupational activities. It is even more difficult for therapists to obtain third-party funding for continued physiotherapy when past strategies have not met with success. Each therapist must assist each client to develop the program that will work best for them. In Ontario, Toronto Rehab has held three annual conferences (www.torontorehab.com) on mild Traumatic Brain Injury, signifying its implications and challenges are relevant across health care disciplines. There is a growing body of literature on the effects of a mild concussion or mTBI. The sports world is leading this research because of the high number of athletes, hockey and football players in particular, who sustain repeated concussions. There is recent evidence of long-term sequelae. These sequelae are very similar to those found in people with persistent post-traumatic neck pain. In research on non-athletic injuries, there is a gap in evidence linking persistent pain with effects of concussion. Few studies of post trauma neck injury have recorded any signs and symptoms other than those related to pain, depression or anxiety. There is no consistency in terminology or methods for reporting these issues, or indeed, what issues should be reported. There are several reasons people with post-traumatic neck pain were never diagnosed with a head injury, including , but not limited to: 1) no report of loss of consciousness 2) no direct head trauma, 3) lack of recognition of unusual behaviour, extent of injury, signs of concussion, 4) no standard objective assessment by initial response personnel (no Glascow Coma Scale score or other outcome measures), 5) inadequate documentation at the time of the trauma and initial post-trauma management, 6) no standard follow-up of the trauma victim, 7) lack of findings of imaging (x-ray or CT scan), or 8) focus on the more critical acute injuries which may have masked the

symptoms of a possible brain injury and no follow-up once stabilized and discharged. We postulate that at the time of the initial neck trauma, two separate events occurred: one related to a physical, structural soft tissue/joint injury to the neck and the other to a shearing force injuring delicate neural structures in the brain/mid-brain. It is important that both issues be recognized, diagnosed, assessed and treated appropriately to ensure a successful rehabilitation outcome.

**Description:** This one hour interactive session will use a case-study based approach, with small group discussion and scripted role- playing exercises.

1. Introduction (15 minutes): We will introduce the topic, review the literature and clinical challenges of working with this population. Includes: update on our own understanding of the aetiology of whiplash-associated disorders, findings that suggest the possibility of ’other’ concomitant physical trauma, and the approach and conclusions often taken by psychologists or mental health professionals when treating people with persistent pain.
2. Practical Session (15 minutes): Participants will divide into small groups for scripted role-playing, using the outcome measures, setting rehab goals and discussion.
3. Summary and Goal Setting (20 minutes): Summary of small group sessions, question and answer period, prioritizing and developing realistic treatment goals based on these outcomes.
4. Conclusions and Wrap-Up (10 minutes): Wrap-up and organization of continuing practical support for therapists who want to use this approach in their clinical setting.

**Conclusion:** Therapists will be presented with clinic-ready assessment tools and questionnaires complete with scoring instructions and interpretative material. They will be able to apply this knowledge and approach immediately on their return to the clinical setting. This will allow clinicians a more comprehensive approach to helping people with complex, multifactorial persistent pain and functional problems. The presenters will also offer physiotherapists the opportunity to participate in a post-workshop electronically- linked interest group. The framework used to manage this population in the therapists own clinical setting will ensure that physiotherapists are more confident about solving practical problems and are able to address other clinical issues that may impede recovery.

**Keywords:** persistent post traumatic neck pain, mild traumatic brain injury

# Abstract: P002 – DEVELOPING EFFECTIVE REHABILITATION PROGRAMS: THE STORY OF THE HIP SURVEILLANCE CLINIC FOR CHILDREN WITH CEREBRAL PALSY AT ALBERTA CHILDREN’S HOSPITAL

**Published:** 2012-05

**Authors:** K Hurtubise

**Affiliations:** Alberta Children’s Hospital, Neurosciences Program, Calgary, Alta

**Learning Objectives and Session Content:** This session is designed to introduce learners to the main components and key elements of program development and design in the rehabilitation context. Upon completion of the session, the learner will:

1. Identify factors within the rehabilitation planning contexts that can influence decisions to revise or develop new programs.
2. Describe data collection methods and analysis process that assist in highlighting the needs of targeted population
3. Discuss the value of using a systematic approach to formulate plans to assess program processes and identify program outcomes.

**Relevance to Physiotherapy Profession:** Physiotherapy managers and clinical leaders often find themselves responsible for developing, planning, guiding, implementing and evaluating programming. Regardless of the complexity of the program, reliance on a program development model to guide the process of applying appropriate theories, assessing the needs of population and the environment in which program planning will be delivered and evaluated will highly increase the likelihood of achieving stakeholder support, securing funding for implementation, and meeting the desired outcomes for the targeted population.

**Target Population:** This session is designed for physiotherapists working in the public or private sector interested in acquiring knowledge and skills to develop more effective rehabilitation programs.

**Description of Supporting Evidence:** As health care agencies and funders strive to make the best decisions about how to use scant resources, health providers are being increasingly asked not only to document what they have done but also what they would like to do, and why (Stanton, 1999) Bravemand and Kielhofer (2004) highlighted that program development models are used to guide decisions about where, how and when to intervene given a chosen problem or target population. Kettler et al (1999) described a step-by-step process for effectivess-based program planning. The process advocates identifying a problem before assessing the need for a program, then using that information to inform the development of program goals, objectives, program design, program budget and finally evaluation plans. These steps are designed to produce a clear understanding of about clients and the changes needed to be achieved in their lives while recording data useful for understanding not only the clients progress and the relevance of the intervention but also information necessary to support good program management (Kettler et al, 1999).

**Description:** The session will be present the keys concepts of program development, reinforcing them with a case example.

**Conclusion:** The healthcare environment is plagued with a plethora of challenges which include lengthy wait-times, unmet patient and client needs and rising cost. Health professional innovation is key to meeting these challenges. Developing knowledge and skills in program development and design is vital in positioning physiotherapists as health system innovators and crafting new models of practice which better meet the needs of their patients and clients.

**Keywords:** program development, program design, cerebral palsy

# Abstract: P003 – EARLY MOBILITY IN CRITICAL CARE

**Published:** 2012-05

**Authors:** N Cote

**Affiliations:** Physiotherapy Department, Toronto General Hospital, University Health Network, Toronto, Ont

**Learning Objectives and Session Content:** The proposed topic for the special education session will discuss the information required to progress our skills into the advancement of early intervention in the critical care setting. The presentation will examine the current literature and practice of mobilization in the intensive care unit. We will explore the methods in which to progress a patient from bed rest to ambulating on a ventilator and Novalung. The discussion of parameters and principles of assessment in the critically ill will occur in addition, there will be a review of relevant lab values and other values such as ventilator settings which are important in the implementation of a mobility program in critical care. The equipment needs and principles to creating an early intervention program in you facility will also be addressed. This session will discuss these algorithms and give concrete guidelines that will assist in progressing the patients’ rehabilitation in the critical care setting.

At the end of the session, the attendees will be able to:

1. Discuss the essential components required to progress a mobility program with a ventilated patient.
2. Be able to evaluate the patients in the intensive care unit and be able to determine who would be a good candidate in the progression of mobility.
3. Be able to discuss possible issues such as labs, respiratory and hemodynamic values which could raise flags or contra- indications to ambulate a ventilated patient.

**Relevance to Physiotherapy Profession:** The move towards early mobility has advanced in the last 10 years, unfortunately many therapist are uncomfortable and unfamiliar with the techniques required to progress the mobility. The path to best practice in the critical care setting is at times unclear; there are few courses that introduce this specialty and offer any insight in the possibilities of treatment options in the intensive care units. This education session will allow group discussion as well as key steps to guide physical therapist in the continuation of rehabilitation with the critically ill patient.

**Target Population:** This session is targeted to physiotherapist, professional leaders and educators working in acute and/or critical care setting as well as therapist working in casual weekend positions in the intensive care units.

**Description of Supporting Evidence:** Mobilization of the critically ill has been in the forefront of the literature in the recent past. It is shown in prospective observational study that long periods of inactivity can cause severe weakness, delirium, muscle atrophy and neuropathy. (Herridge 2011, Korupolu 2009) Keeping patients awake and starting their rehabilitation process in the ICU is now the focus of many centers across North America. Randomised control trials show that early intervention of full body rehabilitation is safe and results in better functional outcomes at hospital discharge. (Schweickert, 2009) It also demonstrates shorter length of stay in the intensive care units and hospital stay. By shortening the length of stay in both the ICU and hospital, intervention has a cost saving element. Algorithms’ have been created to assist clinicians in their taught process when initiating a mobility program. (Stiller, 2007) There are various papers exploring the role of lab values such as haemoglobin, INR and platelet counts to guide the clinicians in their practice.

**Description:** The session will be a lecture format and will allow group discussion of personal experiences, barriers and successes. A video of a ventilated patient will be shown to assist in the visualization of the technique. It will give the opportunity for the attendees to ask questions and receive information that may not be found in the literature.

**Conclusion:** Having the opportunity to discuss with therapist across Canada, we have found that there is a lack in the training opportunities for therapist working in the cardio-respiratory critical care setting. This will give the opportunity to not only learn new skills; it will allow discussion for therapist in the cardio-respiratory field. Understanding the implications of the multiple factors in the intensive care unit is essential for the implementation of best practice for early mobility. Best practice starts at the bed side, with the ventilated patients in the intensive care unit. The awareness of the possibilities is the critical care setting has the potential to advance practice and possibly lead to new research.

**Keywords:** early mobility, critical care, rehabilitation

# Abstract: P004 – THE ACPAC (ADVANCED CLINICIAN PRACTITIONER IN ARTHRITIS CARE) PROGRAM TRAINED EXTENDED ROLE PRACTITIONER (ERP): CAN THEY FILL THE GAP IN POST LICENSURE EDUCATION AND PRACTICE IN ARTHRITIS CARE?

**Published:** 2012-05

**Authors:** K Lundon, C Kennedy, L Soever, S Lineker, L Passalent, R Schneider, R Shupak, K Warmington

**Affiliations:** * University of Toronto; † Martin Family Centre for Arthritis Care and Research; ‡ Mobility Program Clinical Research Unit of the Li Ka Shing Knowledge Institute, St. Michael’s Hospital; § Mount Sinai Hospital; ¶ The Arthritis Society; ** The Hospital for Sick Children Toronto, Ont

**Learning Objectives and Session Content:** Attendees will:

1. Understand the rationale for and the processes involved in the inception of the ACPAC program.
2. Have a sense of the results of the program’s competency-based evaluation.
3. Appreciate extended practice roles through the practice and role utilization changes presented by one program graduate.
4. Understand the impact of practising ACPAC ERPs, as illustrated by the results of a multi-study system-level evaluation.

This presentation offers an overview of the inception, development, and implementation of the ACPAC program as well as data relating to the advanced knowledge, skills and competencies gained through training (20 minutes). The second component reviews aspects of practice and role change achieved by an ACPAC ERP in the 4 years following graduation (20 minutes). The third component reports on the results of the system-level evaluation assessing the impact of practising ACPAC ERPs in Ontario (20 minutes).

**Relevance to Physiotherapy Profession:** Provincial changes in scope of practice, and regulatory changes related to authorized acts (e.g., ordering of diagnostic imaging and certain laboratory tests) are underway. In order to practise these authorized acts physiotherapists are expected to engage in post-licensure programs that address theoretical and practical elements related to advanced practice. The ACPAC program is one such educational initiative, and the program and its graduates have been rigorously evaluated.

**Target Population:** This session will be of interest to educators, physiotherapists, ACPAC graduates, hospital administrators, regulatory bodies and healthcare policy makers.

**Description of Supporting Evidence:** The ACPAC program is an innovative academic and clinical post-licensure training program in arthritis care offered to experienced physical and occupational therapists. It was developed in Toronto, Canada in 2005 with 37 program graduates currently practising as ERPs in diverse clinical settings across Ontario.

Graduates of the ACPAC Program have demonstrated knowledge and skills for practising as ERPs enhancing the available human health resource pool for patients with arthritis. A system-level evaluation of the impact of these ERPs as a new human health resource on patient care and interprofessional relationships in various clinical practice settings has been conducted.

**Description:** Symposium: 60 minutes; three speakers.

Part I. Development and Early Evaluation of an Interprofessional Post-licensure Education Program for ERPs in Arthritis Care Speaker: Katie Lundon BScPT, MSc, PhD Purpose: The ACPAC Program was developed to train experienced physical and occupational therapists as ERPs. The aim was to facilitate optimal, timely, and appropriate delivery of health care to patients with arthritis. This presentation will focus on (1) the development and leadership of the ACPAC Program and (2) trainee performance over time as well as early quantitative and qualitative changes in clinical practice roles for the first three cohorts of graduates (*n* = 19). Methods: Measurement of change in skills and knowledge involved standardized baseline and end-of-program examinations and self-evaluation in areas of clinical competence. Practice-focused surveys issued at three time points during the program and at 6 and 12 months after graduation, evaluated the ERPs’ integration of advanced knowledge and skills. Results: Participants significantly increased their scores on examinations of clinical knowledge (*p* < 0.001) and skills (*p* < 0.001) from baseline through program completion. There was an increase in frequency of performance of clinical tasks and assumption of responsibilities related to their extended practice roles from the beginning to the end of the program. The five areas that changed included increased clinical responsibilities, efficiencies in practice settings, roles as educational leaders and mentors in the field of arthritis care, interprofessional collaboration, and improved access to care for patients with arthritis, particularly in remote areas.

Part 2: Narrative Account by an ACPAC ERP: Role and Responsibilities, Practice Changes, Clinical and Administrative Perspectives Speaker: Leslie Soever, BScPT, MSc, ACPAC. Part 2 is a narrative account, by an ACPAC ERP, of her working role. This ERP will detail her daily responsibilities working in both rheumatology and orthopaedics. Responsibilities include triaging patients for early detection of inflammatory arthritis and for total hip and/or total knee replacement; provision of education regarding conservative management strategies for arthritis; and conducting follow-up visits to assess the status of arthritis-related conditions, joint replacements and medication tolerance. Additional required competencies include ordering/interpreting radiographs; ordering and interpreting laboratory results; providing musculoskeletal differential diagnoses; and identification of ’red flags’. These competencies, attained as part of the ACPAC Program curriculum, will be described. Clinical examples will be utilized to illustrate role impact from the patient; ACPAC ERP; physician; and administrator levels.

Part 3: System-Level Outcomes: Snapshots Speaker: Carol Kennedy BScPT, MSc Part 3 will highlight the findings of the multi- study, two-year system-level evaluation of the ACPAC ERPs.

A. Clinical Utilization and Outcomes Quadrant: This quadrant evaluated clinical performance with respect to patient care provided in various settings examined at a broad system-level describing indicators related to volumes, referral sources and diagnoses of patients seen by ACPAC ERPs. Outcomes were captured via electronic survey administered to graduates quarterly over a 2-year period. Community practice was studied using a retrospective chart review of clients treated by The Arthritis Society’s ACPAC and non-ACPAC program trained therapists. Results include rural versus urban practice patterns, volumes, diagnostic categories, and roles in adult and paediatric arthritis populations.

B. System Integration and Change Quadrant: This quadrant measured the extent to which ACPAC ERPs are delivering integrated and timely healthcare for patients with arthritis. Included in this quadrant were indicators describing ERP roles; integration with community resources and other professional healthcare services, as well as access to ERP care. Methods included an electronic survey administered quarterly over a 2-year period and a cross-sectional self-report survey that investigated interprofessional collaboration within teams that included an ACPAC ERP. Results explore increased efficiency through use of medical directives; roles related to indirect patient care activities such as education, research and leadership; and factors related to interprofessional collaboration.

C. Client and Stakeholder Perspectives Quadrant: This quadrant examined the perspectives of clients who had received care from ACPAC ERPs. Patient perspectives were captured in a cross-sectional self-report survey administered to ERP clients from fifteen institutions. In addition, qualitative focus groups with ACPAC ERPs and interviews with their administrators and clinical colleagues were conducted to determine their satisfaction with the ERPs. Results related to client, worker, and stakeholder satisfaction will be presented.

D. Financial Perspectives Quadrant: This quadrant explores the potential financial impact of ACPAC ERPs on the healthcare system. Focusing at the system and patient-levels, this assessment uses qualitative impressions, system-utilization outcomes and preliminary cost per patient data in the development of financial indicators. Suggested indicators will be reported.

**Conclusion:** Graduates of the ACPAC program have demonstrated knowledge and skills for practising as ERPs that enhance the available human health resource pool for patients with arthritis. The ACPAC program represents a significant innovation in post- licensure education in advanced arthritis care for physical therapists.

The ACPAC ERP may improve wait times for care and provides an interprofessional approach to managing patients with arthritis and other musculoskeletal disorders. This new human health resource may be an effective strategy to address the progressive decline in the number of arthritis care specialists.

**Keywords:** extended role practitioner (ERP), advanced practice, post-licensure education, system level evaluation

# Abstract: P005 – NATIONAL HIP FRACTURE TOOLKIT: REHABILITATION TO RETURN PATIENTS HOME

**Published:** 2012-05

**Authors:** McGlasson, L Beaupre

**Affiliations:** Holland Orthopaedic and Arthritic Centre, Sunnybrook Health Sciences Centre, Toronto, Ont; Departments of Physical Therapy and Surgery (Division of Orthopaedic Surgery), University of Alberta, Edmonton, Alta

**Learning Objectives and Session Content:** This symposium will be presented by Rhona McGlasson (Executive Director, Bone and Joint Canada) and Dr. Lauren Beaupre (Faculty of Rehabilitation Medicine, University of Alberta). These individuals were part of a multi-disciplinary group of clinician, researchers and policy-makers who developed the National Hip Fracture Toolkit during 2011 through Bone and Joint Canada. This symposium will provide information on resources available to physical therapists to apply in their own setting to improve patient outcomes following hip fracture.

Objectives:

The session will be designed with the following objectives;

- To learn about the best practice in rehabilitation for hip fracture patients including the management of system and clinical issues from time of admission to hospital through to post-acute recovery in the non-hospital settings
- To learn about opportunities for physiotherapists to increase the success of returning patients home through system improvements including surgery for weight bearing and access to and delivery of rehabilitation in various settings
- To learn about the development process of the National Hip Fracture Toolkit and how physiotherapists can access and implement the information in their own setting

Content:

Over the last year Bone and Joint Canada worked with experts including surgeons, health professionals, administration and government representatives from across the country to develop a National Hip Fracture Toolkit which was completed in June 2011. This Toolkit provides a comprehensive Model of Care designed to address the complex needs of the hip fracture patient population, including information about system design as well as clinical information for surgery and medical management through the pre- operative, surgical and post-operative including rehabilitation stages of care. The Toolkit has been designed to reflect a holistic view of care provided to the patient through their complete experience and therefore includes best practices in the form of prevention of complications, medical management using geriatric principles, patient and family education and the promotion of active and intensive functional recovery to increase patient’s functional tolerances through a coordinated multidisciplinary approach. The Toolkit provides specific clinical recommendations on the management of this frail patient population including the need for patients to receive early surgical intervention, to promote surgical treatment which ensures weight bearing immediately post surgery, the management of the 3 D’s of delirium, dementia and depression, prevention of post-fracture complications, appropriate access to rehabilitation, and the need for structured discharge planning that includes secondary prevention. Sections are also included that outline the systems approach to care including addressing barriers to accessing surgery and rehabilitation by promoting best practices in organizational efficiency and transition between units or health service providers. With the focus on providing effective care to return patients home there is also the need for a coordinated approach to medical follow up and rehabilitation in the community to enhance patient’s ongoing recovery and functional improvements up to one year post injury. Through the development of the National Hip Fracture Toolkit an evaluation framework has also been created to facilitate performance measurement at a system level as well as clinical measures of care.

As well as the development of the Toolkit the BJC team of experts received resource documentation that can be used to assist health care providers in the implementation of best practices such as clinical care maps, pre-printed orders, educational materials for patients and health care professionals and guidelines, etc. These documents are available to publicly funded health care providers to support the implementation of best practice. The presentation will provide the information on these tools and how they can be accessed and used.

Work is currently underway in all the provinces to measure their current performance against the recommendations in the Toolkit and identify opportunities for improvement which in many regions includes investment in rehabilitation. Further work is being undertaken to improve surgical performance and develop a performance measurement system that can measure the effectiveness of individuals programs as well as the systems and which can be used nationally to directly compare programs. Information about these changes and their effect on the rehabilitation continuum for patients will be shared with the audience.

**Relevance to Physiotherapy Profession:** Almost 30,000 patients a year experience a hip fracture in Canada and for many it is a life changing experience resulting in placement in an institution such as a Long Term Care facility. Patients tend to be older, with an average age of 86 years, and are often frail, presenting with chronic conditions and complex co-morbidities. The National Hip Fracture Toolkit provides a multi-disciplinary framework for the successful management of hip fracture patients with a focus of addressing system and clinical issues so patients are able to return home successfully. The facilitation of functional recovery through enhanced rehabilitation is fundamental to National Hip Fracture Toolkit. Physiotherapists need to know about the best practices, to take a leadership role and work in a multidisciplinary setting with the other health professionals to address clinical barriers and challenge system barriers to care for these frail patients. Knowledge gaps have also been identified to promote a research agenda to address the identified issues.

**Target Population:** Physiotherapists, including senior leaders and management, who work with hip fracture patients across the continuum of care. Prior knowledge of how to manage patients with hip fracture is not necessary for participants to gain an understanding of appropriate hip fracture care.

**Description of Supporting Evidence:** The National Hip Fracture Toolkit was developed using available evidence (reference: References Master List Toolkit, available at www.boneandjointcanada.com Oct 9, 2011). Where there was no evidence available, a consensus approach was used including input from multidisciplinary experts across the country and through numerous teleconferences to allow clinicians and administrators to provide input into the final model of care.

**Description:** The session format will be a presentation by the two authors as well as a guided tour through the website resources. Participants will also have the opportunity to discuss barriers and facilitators to hip fracture care across the country. This will provide attendees the ability to speak to provincial and local strategies to improve patient care to promote knowledge translation.

**Conclusion:** Through the dissemination and implementation of a National Hip Fracture Toolkit will receive improved care at reduced cost to the health care system by:

- Improved access to timely surgery
- Increased acute care capacity through reduced lengths of stay
- Early transfer to active rehabilitation for a reduced length of stay
- Improved access to osteoporosis management for the prevention of future fracture
- Increased patient discharges home to reduce patients being streamed into long term care
- Improved outcomes for seniors – specifically for those with Alzheimer’s and related dementias returning home

Physiotherapists are fundamental to the success of the patients’ recovery following hip fracture providing care in the acute care setting as well as in multiple post-acute settings and in the community. This comprehensive approach to rehabilitation is needed to maximize recovery following a hip fracture which may allow more individuals to return to their pre-fracture level of function and independence.

**Keywords:** national toolkit, hip fracture

# Abstract: P006 – PREVENTION AND MANAGEMENT OF OSTEOARTHRITIS: AN EVIDENCE-BASED PERSPECTIVE ACROSS THE SPECTRUM OF DISEASE

**Published:** 2012-05

**Authors:** C Emery, L Woodhouse, A Jones, L Beaupre

**Affiliations:** * Faculty of Medicine and Kinesiology, University of Calgary; † Faculty of Rehabilitation Medicine, University of Alberta, Edmonton, Alta

**Learning Objectives and Session Content:** This evidence-based symposium will be delivered by four speakers who are clinician scientists and Principal Investigators in “Alberta Innovates Health Solutions Team Osteoarthritis: Creating Bone and Joint Health from the Bedside to the Bench and Back Again Reducing the Burden of Osteoarthritis - from Mechanisms to Prevention”. This symposium will provide practical, evidence-based perspectives from a panel of physiotherapists who are involved in clinical research programs that focus on prevention, detection and management (conservative and post-operative) of osteoarthritis.

Learning objectives:

1. Primary prevention – To understand the implications of/and practical tips for injury prevention in reducing the risk of osteoarthritis from an evidence-based perspective.
2. Secondary prevention – Be exposed to evidence examining early detection of knee osteoarthritis and approaches to prevention of disease progression.
3. Tertiary prevention – To be exposed to an evidence-based perspective related to rehabilitation including conservative management and post-operative management following total knee joint arthroplasty.
4. Be motivated to incorporate evidence-based primary, secondary and tertiary prevention approaches into clinical practice.

**Relevance to Physiotherapy Profession:** Osteoarthritis is the most common type of arthritis. The public health burden of osteoarthritis is significant; affecting 1 in 7 Canadians. While osteoarthritis increases with aging, for many other reasons (i.e. injuries, obesity, and others) its incidence and prevalence are escalating. As there is no definitive “cure” for osteoarthritis, management consists of conservative therapies to mitigate symptoms of pain and reduced function or surgical replacement of the joint. At present, physiotherapists typically become involved in the management of individuals with osteoarthritis only after they become symptomatic and a diagnosis, or referral for joint arthroplasty, has been made. However, the profession has much more to offer across the spectrum of care – particularly in the area of prevention, early detection and prehabilitation of a population at risk for, the development of osteoarthritis. This symposium will provide an evidence-based perspective that will challenge the profession to be more proactive in the management of osteoarthritis.

**Target Population:** This session will not require prior exposure to evidence related to the prevention, early detection and rehabilitation of individuals at risk of developing or who have been diagnosed with osteoarthritis, or those who have undergone total joint arthroplasty.

**Description of Supporting Evidence:**

Primary Prevention

Sport and recreation injuries are a significant health problem in Canada and the leading cause of injury Canadian adolescents. (1-3) It is estimated that sport injuries account for 50% of all injuries in youth ages 11-18 (Incidence Proportion = 35 injuries/100 adolescents). (4-7) Lower extremity injuries account for the majority of these injuries (60%) with ankle and knee joint injuries consistently the most common type accounting for 35-40% of all sport injuries in youth. (5,6) These are associated with an increased risk of osteoarthritis (7-15). Approaches to identifying risk factors and prevention strategies to reduce the risk of joint injury will be highlighted based on systematic reviews, cohort studies and randomized controlled trial (RCT) evidence.

Secondary Prevention

Early detection, diagnosis and prevention of progression

Osteoarthritis is a progressive disorder of unknown cause that results in joint pain and reduced function. Diagnosis of primary osteoarthritis is made on the basis of symptomatology (joint pain, stiffness and tenderness), risk factors (e.g. age, obesity), clinical (restricted ROM, local inflammation, crepitus), functional (self-report and performance based) examination, and confirmed by radiographic changes (osteophytes, joint space narrowing or cartilage loss, subchondral sclerosis or cyst formation, subluxation). Early detection and management of the osteoarthritis can slow its progression and improve health and quality of life (16-18) through management of modified risk factors (e.g. obesity, activity/inactivity). When conservative management is fails, total joint arthroplasty becomes the treatment of choice.(19) At present, there are no standardized universal guidelines that provide indications for when it is the right time to perform total joint arthroplasty.(20) Surveys of orthopaedic surgeons suggest that severe pain and disability are required indications for surgery.(21,22) Some surgeons feel that radiographic changes are also required,(21) others believe this to be of minor importance.(22) As diagnosis of osteoarthritis and the decision to triage to conservative vs. surgical management appears to be based on assessment of pain and physical function, physiotherapists should be playing a more central role in this process. Studies of diagnostic accuracy and the influence of the clinical, functional and radiographic components when examining an individual with knee osteoarthritis will be discussed.

Tertiary Prevention

Conservative management following diagnosis

Standards of care based on the current best evidence have been established for diagnosis and treatment of osteoarthritis.(23-25) Conservative management includes pain relief, maximizing function, preventing disability and patient education; however, a number of risk factors that predisposes a person for functional decline need to be considered.(26) Risk factors such as lifestyle/inactivity, obesity and muscle weakness can be modified and requires changing health behaviour and learning skills to manage osteoarthritis. Comorbidities are associated with older age and can affect the prognosis and outcomes of osteoarthritis.(27) Joint related impairments such as pain, limited joint range, joint laxity and decreased position sense are predictors of functional limitations associated with osteoarthritis of the knee.(28,29) Associations between the level of exercise and functional limitation are reported with osteoarthritis, with aerobic and vigorous exercise having a protective effect.(28,29) Understanding the contribution of osteoarthritis, comorbidities and modifiable risk factors in relation to functional limitation will be addressed. Approaches to managing patients based on current evidence will also be discussed.

Post-operative management

Total knee arthroplasty (TKA) is an accepted standard of treatment for end-stage osteoarthritis and it’s incidence in Canada is rapidly increasing. There were 47,249 knee replacements performed in 2008–2009, representing a 139% increase over 10 years. Further, there are trends towards younger patients undergoing this procedure; the largest increases in TKA occurred in the 45–54 year old age group for both males (271%) and females (337%). (30) Patients who undergo TKA typically report improvement in pain and function within the first 6 months following surgery. (31, 32) Some patients, however, experience little or no improvement in physical function and pain status post-operatively.(33-35) Approaches to managing patients following TKA will be addressed based on current evidence available in systematic reviews, cohort studies and RCTs. We will also address characteristics associated with lower recovery following TKA.

Reference list (1–35) available on request.

**Description:** This interactive symposium will include a combination of lecture and dialogue between four clinician researchers who will present for 10 minutes each followed by 20 minutes of discussion/questions and answers.

**Conclusion:** Traditionally, physiotherapists have played a significant role in the management of individuals with moderate to severe osteoarthritis using non-pharmacologic and non-surgical strategies to minimize pain and maximize function. While these strategies have met with some success in reducing symptoms and delaying the need for surgery, the profession has played a more limited role in primary prevention strategies aimed at reducing the risk of developing osteoarthritis. This evidence-based perspective across the lifespan will challenge physiotherapists to consider their roles in the prevention, early detection and management of osteoarthritis.

**Keywords:** osteoarthritis, prevention, evidence-based

# Abstract: P007 – COLLABORATIVE IDENTITY: CANADIAN OCCUPATIONAL THERAPIST ASSISTANT AND PHYSIOTHERAPIST ASSISTANT EDUCATORS

**Published:** 2012-05

**Authors:** D Dickinson, G Torrance

**Affiliations:** Canadian Occupational Therapist Assistant and Physiotherapist Assistant Educators Council; Humber Institute of Technology & Advanced Learning, Toronto, Ont.; Medicine Hat College, Medicine Hat, Alta

**Learning Objectives and Session Content:** At the end of this session, participants will be able to:

1. Discuss the development, organization and role of the Canadian Occupational Therapist Assistant and Physiotherapist Assistant Educators Council within Canada;
2. Discuss how the growth and development of educators in this field creates professional identity and facilitates collaboration within the broader context of Occupational Therapy, Physical Therapy, and Health Care in Canada.
3. Recognize the challenges and barriers that exist when addressing Occupational Therapist Assistant and Physical Therapist Assistant education and practice in Canada.

**Relevance to Physiotherapy Profession:** Physiotherapist Assistants and Occupational Therapist Assistants are working in increasing numbers and expanding roles in health care across Canada and around the world. The role and use of Therapist Assistants has been facilitated through the collaborative organizational efforts of Canadian Occupational Therapist Assistant and Physiotherapist Assistant Educators over the past twenty years. It is important for physiotherapists to understand the historical development, growth, and organization of Canadian Occupational Therapist Assistant and Physiotherapist Assistant Educators, recognizing that future practice within Physiotherapy will continue to be impacted by the use of Therapist Assistants. It is vitally important that the profession as a whole comes to understand the education of Therapist Assistants and to recognize those Occupational Therapists and Physiotherapists who are practising in this field. It is also important to recognize that Occupational Therapists and Physiotherapists have the opportunity to choose this professional practice area as their career path. Finally, it is important to recognize that this group of educators have embodied the concept of collaboration within itself and through collaboration with professional associations, professional regulators, employers, colleagues and the public at large.

**Target Population:** This session will be of interest to a broad range of professionals including regulators, clinicians, managers, professional leaders, educators, and researchers interested in education, best practices, and innovation in both education and practice.

**Description of Supporting Evidence:** The Canadian Occupational Therapist Assistant and Physiotherapist Assistant Educator Council is a unique group within the professions. There have been previous groups using a collaborative approach in education; however, the development and professionalization within this kind of educator within Canada is relatively recent. There has been little to no research to validate their experience. Through discussions at the recent WCPT Congress, it was evident that the Canadian approach is quite unique.

**Description:** This session will be a lecture format, with opportunity for participant discussion. A facilitated question period will precede the lecture to set the stage for the information provided.

**Conclusion:** The continued growth and collaboration by the Canadian Occupational Therapist Assistant and Physiotherapist Assistant Educators Council will help to meet the ongoing needs of the physiotherapy profession as it expands both its use of and understanding of Physiotherapist Assistant and Occupational Therapist Assistants. Understanding Physiotherapist Assistant and Occupational Therapist Assistant education will help guide current and future practice in physiotherapy, occupational therapy and in health care in general; in the same way, expanding practice models will help to guide education. Physiotherapist practice must recognize this area of professional practice for both the Physiotherapist and the Physiotherapist Assistant. There is also a need to research to guide best practices in both education and clinical practice.

**Keywords:** occupational therapist assistant, physical therapist assistant

# Abstract: P008 – SAFETY IN PHYSIOTHERAPY PRACTICE: BREAKING DOWN THE SILOS

**Published:** 2012-05

**Authors:** J King, CM Anderson

**Affiliations:** * University of Ottawa, Ottawa; † London Health Sciences Centre, London Ont

**Learning Objectives and Session Content:** Provide an overview of what information will be included in the presentation and list up to three objectives describing the purpose of the presentation in terms of what attendees will learn.

1. Participants will be able to apply/understand a more inclusive approach to safety in health care including both provider and patient safety.
2. Participants will be able to identify safety issues in a variety of clinical settings including home care, outpatient clinics, hospital and rehabilitation centre’s perspective.
3. Participants will be able to identify the safety interventions/controls that can be applied at the source, along the path or at the patient/ provider level (hierarchy of control principles).

This interactive session will introduce participants to key concepts in both patient and provider safety and how they relate to physiotherapy practice including hierarchy of control, occupational health and safety and patient safety. Using these concepts, participants will participate in identifying patient and physiotherapy safety issues seen in clinical practice.

**Relevance to Physiotherapy Profession:** Data from Health Canada has indicated that healthcare workers are at greater risk of workplace injuries than any other occupational group. There is also an increasing realization that a culture of provider/staff safety is a pre-requisite to ensuring a culture of patient safety. Historically, physiotherapists have been integral to occupational health and safety interventions (transfers, prevention of back injuries). They therefore, are well suited to be leaders in the combination of provider and patient safety initiatives.

As recognized in the CPA’s position statement for patient safety, regardless of role or setting, patient safety is a concern for all physiotherapists. Physiotherapists and physiotherapy support workers, employed in clinical settings, need to be able to provide safe effective patient care without a risk of injury to the patients or themselves.

Physiotherapists working in administrative or policy roles in health care facilities need to be knowledgeable about both provider and patient safety in planning and evaluating programs. Physiotherapists working in educational roles need to include both provider safety and patient safety in their curricula. Physiotherapists working with regulatory organizations need to be aware of the current safety issues and concerns, and have knowledge of safety strategies for practitioners in order to better protect the public.

**Target Population:** Physiotherapists working in clinical, administrative, educational roles and regulatory organizations.

**Description of Supporting Evidence:** As patient safety has become a key health care concern throughout the world, the link with provider safety is slowly emerging as imperative. These two different safety silos need to be thought of as interrelated. If fact, there is a growing body of research which indicates that healthcare worker health and safety conditions are not just an important but an essential factor associated with the quality of patient care These two aspects need to be examined from a system perspective to prevent harm to all parties. For example, the Campbell Commission concluded that the SARS outbreak was made worse due to the breakdown at many levels of the health care system resulting in negative outcomes for both patients and workers. In fact, the lack of preparation against infectious diseases not only affected patients` lives but failed to protect health care providers who also became sick.

A list of key provider safety and patient safety references will be provided to the participants.

**Description:** This 1-hour interactive session will include an introduction to provider and patient safety concepts for the participants. The session will include an interactive group analysis of clinical situations and identification of patient safety issues in a variety of clinical settings.

**Conclusion:** It is hoped that by breaking down these two silos in safety, participants will have a better understanding of safety as a whole resulting in optimized safe care for patients, while keeping themselves safe. This will have a direct positive impact on patient safety outcomes and reduction in health care cost.

**Keywords:** patient safety, provider safety, occupational health and safety

# Abstract: P009 – CAN YOU ANSWER THIS QUESTION: HOW MANY PHYSICAL THERAPISTS DO WE NEED BY 2030?

**Published:** 2012-05

**Authors:** MD Landry

**Affiliations:** Physical Therapy Division, Department of Community and Family Medicine, Duke University, Durham, N.C., USA

**Learning Objectives and Session Content:** Health human resources (HHR) remain a top priority within health policy and planning debates across Canada and in the global context. In Canada and other so-called ’high-income’ countries, the gap between demand for physical therapy and the supply of physical therapists, is often discussed in terms of level of access to the right provider, at the right time, in the right place, and of course for the right price. Other countries, such as those considered ’middle-income’ or ’low- income,’ often position this debate in terms of no access at all due to workforce shortages. Nevertheless, a common feature across the income gradient of most countries is the pervasive concern that there is not a stable and adequate physical therapy human resource base to meet current demand, let alone how to address an expected sharp rise in future demand. There are three objectives for participants in this session: first, to learn about current knowledge and trends related to supply and demand for physical therapists across Canada and industrialized nations; second, to review, discuss and critique methodologies used to measure supply and demand, along side ’stock-and-flow’ modeling techniques used to forecasting future demand for human resources; and third, to explore and develop a series of data points and assumption that can be used to answer the question: ’how many physical therapists do we need by 2030?”

**Relevance to Physiotherapy Profession:** The issue of balance and/or imbalance between the supply and demand curve spans across all practice settings in which physical therapists are involved. There continues to be a debate about how many physical therapists are currently needed, and often times it is ambiguously stated that “more is better,” but one must ask the question “’how many physical therapists are actually needed, and how should they be distributed?” Moreover, establishing an appropriate future balance between supply and demand could be one of the most important issues that face this profession, especially when considering factors such as an aging population, changes to the scope of practice, perceived substitutions of other regulated and unregulated providers for what physical therapists have historically perceived to be their domain, and an ongoing migration of physical therapists towards community based private for-profit settings. Overall, this session is relevant to physiotherapy profession because it seeks to discuss current directions, but more importantly, because it begins to simulate a future state in terms of physical therapy workforce issue and integrates various scenarios (know as ’what if’ statement) that could worsen or improve workforce imbalances.

**Target Population:** This session may be of interest to all attendees at Congress; there is tremendous scope in this session for all to learn from the ’didactic’ component, but also for all to contribute their opinions and perspectives during the ’interactive’ component of the session. However, exploring the nuances of future supply and demand for physical therapists is likely to have specific implication for the following groups (not listed in order of priority): (1) PT and PTA educators who are planning the structure, content, and cohort sizes of future academic programs, (2) physical therapy regulatory authorities will establish future regulatory framework for an evolving profession, (3) regional, provincial, and national policy makers who are charged with the task of re-tooling health systems to adapt to an increasing and changing demand for physical therapy, (4) local managers and decision-makers who will develop and implement strategies regarding the physical therapy staffing mix within institutions or clinics, and (5) PT and PTA clinicians and students who seek to understand their future place and evolving role in an ever-changing health care landscape.

**Description of Supporting Evidence:** Neoclassical economics defines the notion of ’demand’ in terms of the quantity of a particular service that would be purchased at a given price, and ’supply’ as the availability of finances, practice settings, or human resources. Many scholars have argued that such market forces do not apply to health services, and as such, predicting surpluses or shortages in health professional workforce requires understanding both current and future supply and demand factors. Many health professions, including nurses and physicians, have been engaged in workforce research and planning for many years through robust modeling of the workforce. Physical therapy research has been less aggressive in this area, and as such a significant gap in the literature exists. A recent study based in the United States suggested an important deficit in the number of physical therapists in almost every state by 2030. Although there some methodological critique of the methods used by Zinbelman et al., it represents a move forward. As of yet, there has been no Canadian research into this area.

**Description:** This session will be divided into three distinct components. In the first part, a 20-minute didactic approach will be used to present the ’state of the nation and world’ as it relates to the demand for, and the supply of, physical therapists. In this part, published literature regarding supply, demand and forecasted workforce surpluses and deficits will presented and discussed specific to physical therapists, but also for nurses and physicians. This first section will create a foundation, and will be linked to the second and third part of this session. In the second part of this session, a forecasting model that has been created by the author to predict future physical therapy workforces will be presented. Also in this second part, the participants will be asked to form small groups of 2-4 people in order to critique the forecasting model, provide ideas around the data used in the model, and finally to create a series of ’what if’ statements that will be used to predict future supply demand curves. An example of a ’what if’ statement might be ’what if we increase the number student places in academic programs?’ or maybe ’what is 10% fewer physical therapists aged 50 to 70 yrs retired’? In the third part, we will in real time input such ’what if’ statements into the model to show the predicted effect over time. Overall, the format of the session will include an initial didactic session, which will be follow-up by a much more interactive and interesting real-time modeling of future physical therapy workforce.

**Conclusion:** This session is intended to raise the issues that surround supply and demand for physical therapists, and to what extent projected surpluses or deficits exist in the future physical therapy workforce. The session is positions as a rather ’fun’ and interesting way to explore the future state of human resources. It challenges the participants to think not only about the now, but about the future. A striking feature of this area of health services and policy research is the limited extent to which data exists and which can inform a forecasting model – hence this session will identify this dearth of information and suggest ways in which to advance the field. There is no correct or right answer to our original question of how many physical therapists are needed in the future, rather it is a process that begin with understanding current supply and demand and how such variable are expected to change into the near future.

**Keywords:** supply, demand

# Abstract: P010 – INTEGRATED REGIONAL FALLS PROGRAM

**Published:** 2012-05

**Learning Objectives and Session Content:** Participants that chose to attend this session will leave able to:

1. Start conversations with community partners in context of falls prevention, e.g. Seniors Maintaining Active Roles Together (SMART) exercise program.
2. Speak a “common language” and use standardized outcome measures with falls prevention partners.
3. Identify and remove some of the barriers and obstacles surrounding communication and access to services and generate ideas to improve this communication.

Our description of the program includes a brief review of the components of the integrated network, including: locally based screening clinics throughout the region; family health teams and community health centers, regional and local agencies and organizations. The program also involves Registered Nurses in the ED to provide follow up to seniors who visit the hospital with a fall, and Specialized assessment clinics for more complicated falls which need review by a geriatrician. Linkages with Community Care Access Centre, SMART program as well as seating and mobility clinics to provide complementary care to the senior.

**Relevance to Physiotherapy Profession:** Fear of falling has a strong impact on quality of life for older adults. 1 in 3 community- dwelling seniors fall every year, costing Canadian taxpayers billions of dollars annually. The North Simcoe-Muskoka LIHN has the highest incidence of falls, hospital admissions and hip fractures in all of Ontario, costing healthcare millions of dollars. While healthcare providers and most community partners are aware of the impact falls have, they continue to work in silos, each with a different approach to falls prevention. The Integrated Regional Falls Program (IRFP) exists to promote a standardized approach to falls screening, assessment and management through collaboration and integration with community partners, thus reducing the risk of falls and falls-related injuries in community-dwelling seniors.

Although one of the dilemmas in assessing falls risk is that falls tend to be multifactorial in nature, quantitative research confirms what physiotherapists have known for years: exercise which specifically targets balance is the single most effective intervention to prevent falls in seniors. As physiotherapists have advanced skills in balance and gait assessment, as well as therapeutic exercise prescription, physiotherapists are absolutely essential to a falls prevention team.

**Target Population:** The target population of the presentation is physiotherapists and healthcare administrators who are responsible for monitoring, measuring and reducing the number of falls in the geriatric population.

**Description of Supporting Evidence:** Unintentional falls are the leading cause of injury and accidental death among Canadians over the age of 65 (National Trauma Registry, 2004). In 2004/05 in Ontario (Ontario Injury Prevention Resource Centre, 2007) a senior age 65+ visited the Emergency Department (ED) every 10 minutes and was hospitalized every 30 minutes as a result of a fall. Of seniors admitted as a result of a fall, 31% were discharged to their place of residence, 26% were transferred to another inpatient facility, 26% were discharged to a long term care facility, and 7% died. In 2004/05 North Simcoe Muskoka had the third highest rate of seniors visiting area emergency departments as a result of a fall and the highest rate of both seniors hospitalized with a fall AND with a fractured hip when compared to other LHINs.

These statistics represent only a small fraction of the problem in regards to the impact of falls. The physical, emotional, psychological, and financial impact of falls on seniors and their caregivers in the community, who have not visited the ED or been hospitalized has further implication.

The goal of this program is to provide seniors and their caregivers with an integrated continuum of community-based services to enable them to stay healthy and live more independently in their own homes. It addresses falls throughout the continuum from Prevention to Specialized Services with the possibility of early system entry.

**Description:** We would like to open up the session with a describing the program and identifying our community partners, as well as share the data we have gathered and feedback we’ve received since the program was introduced, and then break out into discussion groups, with the intention of seeking ideas for program improvement.

**Conclusion:** Preventing falls in the senior population is a vital for decreasing disability, mortality, hospital stays, taxpayer dollars and most importantly, improving quality of life.

Physiotherapists are absolutely crucial to the achievement of this goal. However, we recognize the importance of collaboration in healthcare. This falls prevention program is one example of how building partnerships within the broader health care community can improve the health and well being of seniors and other at risk populations. Sharing this information is important. We truly hope that this session will help generate conversation and promote systematic sharing of information. We have demonstrated that doing so will improve health outcome as well as cost reduction.

# Abstract: P011 – PLACEMENTS WITH PIZAZZ: USING THEORY TO MAKE CLINICAL PLACEMENTS MEMORABLE

**Published:** 2012-05

**Authors:** SM Murphy

**Affiliations:** Department of Physical Therapy, University of British Columbia, Vancouver, B.C.

**Learning Objectives and Session Content:** By the end of the session, participants will be able to:

1. Describe at least 3 educational theories with relevance to PT clinical education
2. Apply 2 or more educational theories in the planning of a learning experience for PT students in a specific clinical setting
3. Provide examples of how to improve current clinical education practice by using educational theory.

**Relevance to Physiotherapy Profession:** Physical Therapists chosen to supervise students are often experienced practitioners with excellent clinical skills. However, these same clinicians may have limited knowledge of educational theory, which makes planning a student learning experience challenging. By using a theory-based approach, clinicians can plan and deliver a clinical education experience which provides the learner with the optimal chances of success, and provides the tools for lifelong learning.

**Target Population:** This session will be of interest to novice and experienced clinicians who currently provide learning experiences for students in the clinical setting, or to those who are considering doing so in the future.

**Description of Supporting Evidence:** For many clinical supervisors, planning and implementing successful clinical learning experiences for students is largely serendipitous. Planning for clinical learning experiences are frequently based on the supervisors own learning experiences, and may be largely intuitive. This type of clinical learning environment may provide insufficient opportunity for optimal student success and inadequate support for students who experience difficulty. The work of educational theorists provides concepts which are integral to the provision of education in the clinical setting. Ranging from Malcolm Knowles principles for teaching adults, to Schon’s work on reflection, to Lave and Wenger’s theory of legitimate peripheral participation, the work of many theorists has direct relevance to teaching and learning in the clinical setting.

**Description:** This session will consist of interactive lecture and small group discussion. Participants will be encouraged to share their experiences with student supervision and education, and to think conceptually about planning a learning experience for their specific clinical environment which is based on educational theory.

**Conclusion:** In order to promote optimal learning in the clinical setting, clinicians need to plan educational experiences based on more than “gut feel” or their own experience. The work of educational theorists can provide theoretical building blocks which can be used to develop an educationally sound clinical learning experience for a wide variety of clinical settings.

**Keywords:** physical therapy, practice education, educational theory.

# Abstract: P012 – DEVELOPMENTAL COORDINATION DISORDER (DCD): USING KNOWLEDGE TRANSLATION AS A PHYSIOTHERAPY MANAGEMENT STRATEGY

**Published:** 2012-05

**Authors:** LM Rivard, D Levac

**Affiliations:** School of Rehabilitation Science and CanChild Centre for Childhood Disability Research, McMaster University, Hamilton, Ont

**Learning Objectives and Session Content:** Developmental coordination disorder (DCD) is a common neuro-developmental condition that impacts on a child’s ability to learn and perform everyday motor tasks. Poorly developed fine and/or gross motor coordination substantially impact children’s motor skill performance with far-reaching consequences for their daily life activities and scholastic achievement. It has been well documented that children with DCD develop a range of negative secondary consequences related to their primary motor impairment, including reduced physical activity. These children are at risk for developing poor physical fitness, overweight/obesity, poor cardiovascular health, and a sedentary lifestyle. Although it was once believed that these difficulties would diminish with time and maturation, there is now compelling evidence that DCD is a life-long condition, making this disorder one that warrants significant attention.

Given the likelihood of developing secondary consequences, current management of children with DCD using traditional models whereby the therapist works ’one on one’ to treat the underlying motor deficits may not be sufficient to manage all aspects of this chronic condition. Use of an innovative service delivery model that shifts the emphasis away from remediating impairment towards a focus on management and health promotion would be beneficial, particularly in preventing the development of secondary health problems. Using this model, a primary role for PTs includes knowledge translation to enhance the capacity of individuals in the child’s environment (parents, educators) to manage motor difficulties and encourage physical activity participation.

This session will focus on describing DCD and outlining the need for long-term management of the condition. We will discuss an innovative service delivery model that incorporates knowledge translation to promote physical health and enhance the quality of life for children and youth with DCD and empower those who care for them.

This session will enable participants to:

1. Define and describe Developmental Coordination Disorder (DCD), and outline the primary impairments, secondary physical health consequences, and impact on physical activity participation and quality of life.
2. Outline the lack of research evidence for traditional physiotherapy (PT) management of DCD and appreciate the rationale for incorporating a knowledge translation (KT) approach.
3. Apply practical KT implementation strategies relevant to a variety of service delivery models for children with DCD.

**Relevance to Physiotherapy Profession:** Children with DCD are managed by PTs in a variety of clinical settings. PTs may intervene with these children during the preschool years when they are exhibiting delays in motor skill development. The motor difficulties are often “mild” and non-specific and PTs may struggle with how to manage the disorder. In addition, many PTs provide services to children with DCD in school health settings. These settings are often isolating with few opportunities for continuing education related to management of this challenging population. Health promotion is an important aspect of the PT role when working with children and youth with DCD and their families. The idea of knowledge translation as a major component of the PT intervention strategy is novel. Through knowledge translation to children with DCD, their families, teachers, and others in the community, PTs can help children with DCD become more physically active, increase their participation levels and decrease the risk of a sedentary lifestyle. PTs should be encouraged to translate knowledge to the adults in the child’s home, school, and community environments as an essential element of their practice. However, teaching of the skills required for this knowledge translation model is not routinely included in PT curricula.

**Target Population:** This session will be of interest to physiotherapists, physiotherapy students, and physiotherapy assistants working with children and youth with DCD in clinical, administrative or research roles.

**Description of Supporting Evidence:** There is little evidence available to guide effective PT management of this condition. Traditionally, PTs have focused on the remediation of underlying motor components to improve task performance; this approach has not been proven successful, particularly over the long-term. Given the chronic nature of DCD, and findings from research with adolescents and adults with DCD indicating what was helpful to them, it is evident that PT service delivery models need to incorporate management strategies which focus on the prevention of inactivity and decreased fitness, which is so common in children presenting with this disorder.

An emerging rehabilitation service delivery model focusing on the role of the occupational therapist with this population suggests a shift away from direct, hands-on service provision. In this model, therapists are encouraged to educate parents and teachers of children with DCD about the condition and provide information about how to make tasks easier, adapt the environment, ensure that activities are matched to children’s capabilities, and support the participation of children with DCD in meaningful activities. The underlying features of this approach are not discipline-specific, and are also relevant to PT; however, this model has yet to be promoted as a PT intervention approach.

A key element of this KT approach for PT practice is the promotion of physical activity participation in children and adolescents with DCD. PTs can engage and consult with families and educators to increase their understanding of the reasons for children’s motor challenges. Additionally, PTs can enhance the capacity of parents and educators to use strategies in the school environment, and engage in leisure activities that will most likely be successful for children with DCD and in so doing, prevent many of the secondary health problems that have been documented in the literature.

**Description:** This session will be divided into two parts. During the first half hour, the presenters will provide an interactive didactic session to provide general information on DCD, discuss research evidence related to intervention approaches commonly used with this population, and introduce a KT focus. Experiential exercises and varied media will be used including video clips of children and youth with DCD. In the second half hour, participants will divide into small groups to apply their learning. Session presenters will introduce practical KT implementation strategies, facilitate interactive discussion of participants’ clinical experiences, and review clinical scenarios relevant to PT practice in different clinical settings.

**Conclusion:** DCD is a chronic medical condition affecting 5–6% of school-aged children. Health promotion and prevention of secondary physical health consequences must be an essential component of PT management of this population. An emerging rehabilitation service delivery model that places an emphasis on knowledge translation and enhancing long-term health in children with DCD is relevant to PTs practising in a variety of clinical settings. This model of care will be described and related to PT practice. Facilitated group discussion, review of clinical scenarios, and application of practical knowledge translation strategies will provide opportunities for participants attending this session to apply a knowledge translation approach to their clinical setting.

**Keywords:** developmental coordination disorder, DCD, intervention, knowledge translation, service delivery model, health promotion, prevention, physical activity participation

# Abstract: P013 – EARLY IDENTIFICATION OF ANKYLOSING SPONDYLTIS AND SPONDYLOARTHRITIS CONDITIONS

**Published:** 2012-05

**Authors:** M Aisen

**Affiliations:** Arthritis Rehabilitation and Education Program (AREP), The Arthritis Society

**Learning Objectives and Session Content:** At the end of this session, participants will be able to:

1. Identify systemic and familial risk factors and pain patterns that may differentiate mechanical versus inflammatory spinal pain;
2. Perform a standardized physical examination for Spondyloarthritis and administer standardized outcome measures; and
3. Link patients to relevant local community resources and support groups.

**Relevance to Physiotherapy Profession:** Early diagnosis of Ankylosing Spondylitis and related forms of Spondyloarthritis is key to best possible patient outcomes. Delayed diagnosis often leads to potentially preventable physical, functional, emotional and vocational impairments. Physiotherapists can have a significant role in early identification of these patients by facilitating referrals to rheumatologists. We, as physiotherapists, have an urgent responsibility to increase our ability to assess these patients. Once patients receive a confirmed diagnosis, they can be fully informed of treatment options. They may agree to start appropriate medications. Then physiotherapists can coach patients to succeed with their rehabilitation through suitable education and therapeutic exercise modalities — the cornerstone of recommended best practice care.

**Target Population:** This session will be of interest to physiotherapists treating patients with musculoskeletal conditions, such as: acute and chronic spinal conditions; tendonitis; plantar fasciitis; and other enthesopathies, for whom the traditional orthopedic rehabilitation strategies are not ultimately effective. Increased ability to assess and identify Spondyloarthritis can facilitate best possible care.

**Description of Supporting Evidence:** The prevalence of Ankylosing Spondylitis and related Spondyloarthritis conditions, depending on regional differences, is between 0.5% and 1%. The Center for Disease Control and Prevention for the National Arthritis Data Workgroup estimates that Ankylosing Spondylitis and its related diseases affect as many as 2.4 million people in the United States. Once thought to be a disease affecting males to females at a 10:1 ratio, in the United States it is reported to be a 2:1 ratio. Large numbers of patients in the past have reported delayed diagnosis of up to 12 to 18 years. Delays of 6 to 9 years are still not uncommon, despite increased access to Magnetic Resonance Imaging. A significant barrier to early diagnosis is the decreased knowledge of primary care physicians and delayed referral to rheumatologists. They often will refer patients to other allied health professionals. Patients often self refer to allied health practitioners as well. Physiotherapists and chiropractors are often a first point of contact for people with spine and other musculoskeletal symptoms. Many men with mainly axial presentation have little pain and may seek help only when significant calcifications have already impacted their function and posture. Many women may first present with neck pain and stiffness and recurring peripheral symptoms. Women and men who are overweight often have symptoms which are erroneously attributed to lack of fitness and obesity. X-ray findings are generally not useful in the early stages of the disease except in specialized arthritis centers, where radiologists have an increased awareness. Some patients may have a combination of Degenerative Disc Disease and Spondyloarthritis. The Canadian Spondylitis Association reports that Spondyloarthritis is the most overlooked cause of persistent back pain in young adults. There are significant economic costs in the areas of personal care and productivity loss. Early diagnosis and treatment can have a positive impact on reducing these costs to society as a whole. A good history and examination can provide adequate information to suspect a diagnosis in these patients. X-ray changes such as syndesmophytes and calcification of spinal ligaments take years to be evident on X-ray. New biologic medications have helped significantly to reduce or eliminate pain. Then patients can exercise more aggressively without pain and thereby minimizing physical, functional and vocational impairments.

**Description:** This session will combine a brief introductory lecture followed by a practical ’see one -do one’ experiential learning opportunity. All participants will receive a sample assessment form, written guidelines for assessment and a sample exercise handout for Spondyloarthritis patients.

**Conclusion:** Physiotherapists aware of best practice assessment skills for patients with Ankylosing Spondylitis and related Spondyloarthritis conditions can have a significant impact on early diagnosis and minimizing physical, functional and emotional impairments for these patients.

**Keywords:** standardized assessment, inflammatory back pain, spondyloarthritis, ankylosing spondylitis

# Abstract: P014 – MODELING COLLABORATIVE TEAMS: MOVING PRACTICE SUPPORT TO AN INTERPROFESSIONAL LENS

**Published:** 2012-05

**Authors:** TL Dutton

**Affiliations:** Capital District Health Authority, Halifax, N.S.

**Learning Objectives and Session Content:** Within a new professional practice structure created within a large health district, 8 different professional practice coordinators were brought together under one manager, from the disciplines of physiotherapy, occupational therapy, social work, spiritual and religious care, orthotics and prosthetics, psychology and recreation therapy. The team was launched through team building activities which acknowledged and celebrated their individual strength and diversity, and by creating a unified purpose and an accountability framework through the joint development of commitments and goals.

Attendees will have the opportunity to learn about the development of this new interprofessional model of professional practice, as well as have the opportunity to discuss challenges and opportunities related to professional practice structures. Attendees will have the opportunity to be exposed to tools that might help them in their collaborative efforts.

Two objectives:

1. To become familiar with one possible interprofessional model of professional practice support and the challenges and opportunities of developing that model.
2. To be exposed to tools in bringing diverse groups together to enhance collaborative efforts for practice support.

**Relevance to Physiotherapy Profession:** Professional practice models for the support and enhancement of physiotherapy practice are common across different clinical sites in Canada. Within health care teams, there is a new focus on the development of collaborative, interprofessional models which put the client at the centre. Professional practice leadership structures need to embrace and model collaborative practice in order to be role models for staff at the front line.

**Target Population:** Leaders in physiotherapy, including professional practice coordinators, leaders, chiefs, managers.

**Description of Supporting Evidence:** The processes around interprofessional collaborative practice is well supported in the literature and in clinical environments to assist in client-centred practice. The Canadian Interprofessional Health Collaborative reports that collaborative practice can positively impact current health issues such as: wait times, healthy workplaces, health human resources, patient safety, chronic disease management and population health and wellness. It is also identified in the literature that organizational structure can be a barrier to interprofessional collaboration and that leadership commitment is required. Breaking down barriers to collaboration such as lack of trust and respect, turf and power is critical.

**Description:** A lecture presentation will be followed by interactive small and large group discussions. Participants will be able to discuss their own practice contexts relevant to the materials presented.

**Conclusion:** Positioning physiotherapy within an interprofessional context may enable physiotherapists to work to their full scope of practice and provide more client-centred care, as the client is receiving the best integrated care from the most appropriate health care provider. As clients and the health care environment become more and more complex, there will be a continued requirement for enhanced interprofessional partnerships to meet these care needs in a successful, efficient and sustainable manner, both for the client and the health care team. Professional practice structures may be the key enabler to facilitating this collaborative practice.

**Keywords:** collaboration, practice, leadership

# Abstract: P015 – USING ON-LINE RESOURCES TO ENHANCE BEST PRACTICES IN STROKE REHABILITATION

**Published:** 2012-05

**Authors:** EH French

**Affiliations:** Northwestern Ontario Regional Stroke Network, Thunder Bay Regional Health Sciences Centre, Thunder Bay, Ont

**Learning Objectives and Session Content:** This session will provide participants with the opportunity to familiarize themselves with evidence-based, on-line resources which support the implementation of best practice in stroke rehabilitation. A clinical scenario will be used to demonstrate the application of on-line resources to support decision making surrounding best practice in assessment and treatment approaches, as well as patient and family education.

At the end of this session, participants will be able to:

1. Navigate, and identify content contained within, the following on-line resources: 2010 Canadian Best Practice Recommendations for Stroke Care, Evidence Based Review of Stroke Rehabilitation and StrokEngine.
2. Use on-line resources to aid evidence-based, clinical decision making in stroke rehabilitation.
3. Identify knowledge translation strategies to increase awareness of on-line resources in their local setting.

**Relevance to Physiotherapy Profession:** Best practice is integral to the physiotherapy profession as outlined in the following statement:

“Evidence-informed practice means providing the best available care to specific patient populations in a specific clinical setting. The best available care is derived from the interaction between evidence-informed knowledge, clinical experience and patient needs. Physiotherapists are responsible for keeping current with research findings that support physiotherapy treatment interventions and for integrating this information with clinical expertise, patient values and available resources to positively impact the health of Canadians.”

(CPA Position Statement, November 2009 – *Evidence-Informed Practice in Physiotherapy*)

With the growing accumulation of rehabilitation evidence, physiotherapists require tools to help them implement the most up to date, efficient and effective care for their clients. The use of on-line resources is a readily accessible strategy that physiotherapists can use to help them become evidence-informed practitioners.

**Target Population:** This session will be of interest to a broad range of professionals including clinicians, managers, professional leaders and educators interested in the implementation of best practices in stroke care.

**Description of Supporting Evidence:** Knowledge translation literature indicates that the transfer of research into practice can be a lengthy and complex process. Research has shown that high-quality evidence is not consistently applied in practice which can result in patients not receiving the best available care and the inefficient use of health care resources. Graham et al (2006) have proposed a model which describes the components of the “knowledge to action” process. One component of the knowledge to action process is “knowledge creation”. Knowledge creation includes knowledge inquiry, knowledge synthesis and the development of knowledge tools and products. All stages of knowledge creation allow knowledge producers to customize the method of dissemination to best reach the target users of the information. (Ian Graham et al 2006, *Lost in Knowledge Translation: Time for a Map*?)

The Canadian Stroke Strategy encourages stroke leaders to use a variety of approaches to disseminate best practice recommendations and knowledge translation tools to end users which can include healthcare professionals, health system planners, decision-makers, and funders. Presentations at health care conferences across the continuum of stroke care and alerting users about the guidelines and how to access them are specifically identified as part of the Canadian Stroke System’s dissemination strategy. (www.strokebestpractices.ca).

This session will provide examples of knowledge tools and products specific to stroke care that physiotherapists and other health care providers can utilize and apply within a rehabilitation setting.

**Description:** This interactive presentation will make use of a clinical scenario to demonstrate the use of on-line resources that support clinical decision making and the application of evidence-based practice in a stroke rehabilitation setting.

On-line resources will be accessed live on the internet to demonstrate the content available as well as tips for navigating through the individual websites.

The on-line resources that will be highlighted include: Canadian Best Practice Recommendations for Stroke Care 2010 www.strokebestpractices.ca Evidence-Based Review of Stroke Rehabilitation www.ebrsr.com StrokEngine (including StrokEngine Assess & Family) www.strokengine.ca

Small group work will be incorporated by designing activities that will allow opportunities for participants to: 1) discuss how on-line resources supporting best practice could be implemented in their setting. 2) develop a knowledge translation plan to share resources upon return to their practice setting.

**Conclusion:** A number of on-line resources exist that provide current, evidence-based information and guidelines regarding best practice in stroke care. The use of on-line resources are one strategy that physiotherapists can use to quickly and easily access information supporting the implementation of best practice in their clinical setting.

**Keywords:** stroke, best practice, rehabilitation, knowledge translation

# Abstract: P016 – TRANSFORMATIVE LEARNING AND RESPONSE SHIFT: SEPARATE BUT COMPLEMENTARY CONCEPTS TO HELP PHYSIOTHERAPISTS UNDERSTAND THE CHANGE PROCESS FOR PEOPLE LIVING WITH CHRONIC ILLNESSES

**Published:** 2012-05

**Authors:** R Barclay-Goddard, J King, CJ Dubouloz, CE Schwartz

**Affiliations:** * University of Manitoba, Winnipeg, Man; † University of Ottawa, Ottawa, Ont; ‡ Tufts University School of Medicine, Boston, Mass., USA

**Learning Objectives and Session Content:** Being diagnosed with a chronic illness results in major life changes. An individual’s perspective about their circumstances or health related quality of life often changes over time. In the adult education literature, this process of change has been described as “transformative learning” while in health-related quality of life research, “response shift” is considered a change in the meaning of a person’s self evaluation. To help people during their rehabilitation, physiotherapists need to understand these concepts of change.

This interactive presentation will include:

1. A brief discussion of transformative learning and response shift with a focus on rehabilitation and chronic illness-related literature from both fields.
2. The presentation of a case study in which participants will have the opportunity to identify and discuss aspects of change that the client in the case undergoes during rehabilitation. A discussion of how transformative learning and response shift theory can be utilized in treatment will also occur.
3. The early results of focus groups with physiotherapists and occupational therapists regarding their opinions and experiences with changes in perspectives will be shared.

Objectives:

1. Participants will be able to discuss two concepts of change, response shift and transformative learning, as they relate to chronic disease management and rehabilitation.
2. Using these concepts, participants will be able to identify how these concepts of change could be used in rehabilitation and chronic disease management to facilitate clients in experiencing improved health related quality of life.

**Relevance to Physiotherapy Profession:** A major goal of treatment for individuals undergoing rehabilitation and for those living with chronic illness or disability is often to learn new approaches, techniques and strategies for self-management, and to improve health related quality of life. Both transformative learning and response shift have much to offer physiotherapists in understanding the change process for the person in rehabilitation or living with chronic illness or disability to optimize their health-related quality of life.

**Target Population:** Physiotherapists working in clinical, administrative, and educational roles.

**Description of Supporting Evidence:** The results of a recent narrative review of response shift (by RBG and co-authors) and a meta-synthesis of transformative learning literature with people living with chronic illnesses (by Dubouloz, JK and coauthors) were reviewed and theoretical frameworks of the two concepts were compared and contrasted. The two concepts appeared to be related but have been developed in parallel by two different fields of study, adult education and educational learning/organization change. A soon to be published paper discussing the two concepts (by RBG, JK and coauthors) suggested future research which investigates how rehabilitation clinicians can use both concepts clinically. A list of key references will be provided to the participants.

**Description:** This 1-hour interactive session will include an introduction to transformative learning and response shift for the participants. The session will conclude by an interactive group analysis of clinical situations and identification of concepts from both theories for day-to-day practice.

**Conclusion:** At the end of this interactive session the participants will have a greater understanding of transformative learning and response shift. It is hoped that their understanding of these key concepts will help them grow as professionals by providing them with knowledge and skills that could be incorporated in future health care interactions for the benefit of patients.

**Keywords:** chronic illness, process of change, rehabilitation, quality of life, response shift, transformative learning

# Abstract: P017 – ARE PHYSIOTHERAPISTS CONTRIBUTING TO FEAR AVOIDANCE BELIEFS AND CHRONIC LOW BACK PAIN? THE EVIDENCE, THE PROBLEM AND POTENTIAL SOLUTIONS

**Published:** 2012-05

**Authors:** B Jam

**Affiliations:** Advanced Physical Therapy Education Institute, Thornhill, Ont

**Learning Objectives and Session Content:** At the end of this session, participants will have:

1. A greater understanding of the mounting evidence supporting fear avoidance beliefs (FAB) as a significant contributing factor to chronic disability in individuals with low back pain
2. Clinically applicable methods of evaluating the potential presence of FAB in patients with low back pain
3. Immediately applicable and effective clinical skills to reduce FAB and help accelerate return to work and physical activity

**Relevance to Physiotherapy Profession:** Many studies demonstrate that FAB about physical activity are strongly linked to chronic disability due to low back pain. Studies also confirm the importance of clinicians addressing FAB in individuals with low back pain. It has been suggested that the over-medicalization” of low back pain is a major contributing factor to increased patient fears and regrettably physiotherapists may sometimes be inadvertently contributing to this. There is also support that physiotherapists’ ratings of perceived patient fear-avoidance are not associated with fear-avoidance measures.”

**Target Population:** This session will be of interest to physiotherapists in private practice or hospital setting who are involved in direct care of patients with acute, sub-acute or persistent lumbo-pelvic pain.

**Description of Supporting Evidence:** Cross-sectional studies have shown that high levels of FAB occur early in patients with low back pain. To identify this, studies support the use of the FAB Questionnaire as an outcome measure for patients with acute low back pain. The value of addressing FAB has been demonstrated in multiple studies ranging from elderly individuals with chronic low back pain (Basler 2008), those awaiting spinal fusion (Brox 2008) to injured workers (George 2003).

**Description:** This session will be a lecture format, with specific clinical case studies in order to assist immediate clinical implementation.

**Conclusion:** Physiotherapists should not rely solely on their own judgments to identify FAB in patients with low back pain. It is essential for clinicians to have reliable and validated methods of evaluating FAB in order to identify and consequently effectively manage this significant potential contributing factor to chronic disability. By understanding the evidence, clinicians should place greater emphasis on addressing FAB early on, in place of passive pain relieving modalities or specific manual therapies that may be further contributing to passive coping skills and potentially further feeding into patient FAB.

**Keywords:** fear avoidance, catastrophization, low back pain

# Abstract: P018 – INTERVENING TO REDUCE SEDENTARY BEHAVIOUR: A CHANGE IN FOCUS FOR PHYSICAL THERAPISTS AND PEOPLE WITH MOBILITY DISABILITY?

**Published:** 2012-05

**Authors:** PJ Manns

**Affiliations:** Department of Physical Therapy, University of Alberta, Edmonton, Alta

**Learning Objectives and Session Content:** The aim of this session is to increase participant knowledge in the area of sedentary behaviour and health. Participants will learn about methods to assess sedentary behaviour and will be provided with descriptive information about the magnitude of sedentary behaviour (including pattern of sedentary behaviour) in people with and without mobility disability. Information from prospective and cross-sectional studies about the associations between sedentary behaviour and health outcomes will be presented. Finally, examples of sedentary behaviour interventions will be presented and applications to physical therapy and populations with mobility disability will be discussed.

**Relevance to Physiotherapy Profession:** Physiotherapists working in the community often see patients who are generally sedentary, many of whom have mobility disability. Changing activity behavior to meet activity guidelines is, for many people, very challenging and difficult to sustain. With inactive populations a more successful and sustainable approach to behaviour change may be to reduce and break up sedentary time. Physical therapists may be ideal health professionals to educate their patients about sedentary behaviour and health, and work with patients to devise strategies to reduce sitting behavior throughout the day. This proposal addresses the Best Practice Stream and provides new considerations for best practices regarding activity programming for community-dwelling people with mobility disability.

**Target Population:** Physiotherapists who work with inactive populations, particularly those with mobility disability.

**Description of Supporting Evidence:**

1. Measurement of Sedentary Behaviour. Sedentary behaviour has been measured using both self-report and device-based measures. Self-report measures have been found to have variable reliability and validity, similar to self-report measures of physical activity. Device-based measures (most frequently accelerometers) have increasingly been used to measure sedentary behaviour. Devices with inclinometers are the most accurate for recording postural information (i.e. sitting/lying vs. standing). Accelerometers that provide a measure of counts (arbitrary units) have been used most commonly to classify activity behaviour into categories of sedentary, light, moderate or vigorous activity, and there are published cut-offs for each category for older adults.
2. Magnitude of Sedentary Behaviour in People With and Without Mobility Disability. Limited studies have measured sedentary behaviour in individuals with mobility disability. People with stroke are sedentary (sitting or lying) 85-90% of their non-sleep time, which compares to 65-70% in older adults without disability. Fewer breaks from sedentary time have been reported in individuals with Parkinson’s disease and stroke, as compared to controls. More research is required to better describe the magnitude of sedentary time and pattern of sedentary time accumulation in individuals with different levels of mobility impairment.
3. Sedentary Behaviour and Health Outcomes. In the past 10 years, research has more clearly shown that the health impacts of being sedentary (i.e. sitting too much) are not the same as not getting enough exercise. Longitudinal studies show that sitting too much is associated with increased mortality, independent of physical activity status. Increased sitting time, and fewer breaks in sedentary time are detrimentally associated with cardio-metabolic health risk factors, independent of level of physical activity. There is currently no information regarding associations between sedentary behaviour and cardio-metabolic health outcomes in people with mobility disability.
4. Interventions. Interventions for adults, designed to reduce sedentary time, are a relatively new area of study as most activity interventions to date have focused on achievement of moderate physical activity targets. Reducing television time and time in front of a computer screen have been targets of interventions to reduce sitting time. Standing stations in workplaces are becoming more common and studies show that this environmental change leads to less sitting time. A recent study with older adults used an intervention based on social cognitive theory and focused exclusively on reducing sitting time in older adults. They reported a 3.2% reduction in sitting time with the intervention, which amounted to a 30-minute reduction daily. The primary message of that intervention was “move more, sit less” and may be particularly relevant for people with mobility disability who tend to sit more than those without disability. It is possible that for individuals with mobility disability, who are more sedentary than their age matched peers, an intervention to decrease sitting time may have a greater effect than for individuals without disability. To date, there are no interventions with people with mobility disability that have focused on reducing sitting time.

**Description:**

1. Lecture. Two thirds of the presentation time will be information delivered in a primarily didactic format. Participants will be provided with information related to the supporting evidence outlined above.
2. Small group activity. Participants will be taken through parts of the “Stand Up For Your Health” workbook, which has been utilized in an Australian study designed to reduce sitting time.
3. Conclusion/Wrap Up Activity

**Conclusion:** Sedentary behaviour, or prolonged sitting time, has been identified as a unique health risk, independent of leisure time physical activity levels. Recent position stands and advice from expert bodies identify the potential importance of reducing sedentary time. For adults with mobility disability, who tend to be inactive, it may be more feasible and ultimately more successful to start to change activity behaviour with reductions in sedentary time and increases in light intensity activity. Reducing and breaking up sedentary time may be an important approach for physical therapists to incorporate with their patients who are generally inactive.

**Keywords:** sedentary behaviour, mobility disability, health outcomes, measurement

# Abstract: P019 – AEROBIC EXERCISE RECOMMENDATIONS TO OPTIMIZE BEST PRACTICES IN CARE AFTER STROKE (AEROBICS)

**Published:** 2012-05

**Authors:** M MacKay-Lyons, A McDonald

**Affiliations:** * School of Physiotherapy, Dalhousie University; † Nova Scotia Rehabilitation Centre, QEII Health Sciences Centre, Halifax, N.S.

**Learning Objectives and Session Content:** By the end of this session, participants will be able to:

1. Explain the benefits of aerobic exercise post stroke,
2. Describe the development of recommendations regarding screening for, and prescription of, aerobic training for the stroke population,
3. Understand the recommendations and supporting evidence for aerobic training in the stroke population,
4. Discuss the practical application of the recommendations across the continuum of care from stroke onset to community integration.

**Relevance to Physiotherapy Profession:** Knowledge translation proposals have proven to be effective in advancing clinical practice. This session will highlight the clinical relevance of the Aerobic Exercise Recommendations to Optimize Best Practices In Care After Stroke (AEROBICS) to improve rehabilitation outcomes of persons after stroke.

**Target Population:** This session will be of interest to physiotherapists motivated to advance their knowledge and use of aerobic exercise for people post-stroke.

**Description of Supporting Evidence:** A literature synthesis was prepared prior to the development of the AEROBICS that contained findings from clinical trials, systematic reviews, meta-analyses, and existing guidelines that addressed the role of aerobic exercise in stroke management. Topics of interest in the synthesis included the physiological and functional benefits, indications and contraindications of aerobic exercise stress testing and interventions after stroke, as well as considerations regarding implementation across severity of stroke and the continuum of post-stroke care. Information from this synthesis will be presented during this session to support each of the AEROBICS recommendations.

**Description:** The session will include PowerPoint slides, beginning with an overview of the evidence concerning the benefits of aerobic exercise post-stroke. The development of the AEROBIC recommendations will then be described in brief. Next, each recommendation will be presented, along with supporting evidence. Case histories and videos will be used to augment the content. The two speakers will develop the presentation material jointly, based on their relevant expertise. Participants will be encouraged to ask questions and engage in interactive discussion with the presenters.

**Conclusion:** This session is designed to promote uptake of the AEROBICS recommendations by Canadian neuro-physiotherapists in their daily clinical practice, with the ultimate goal of improving outcomes for people after stroke.

**Keywords:** stroke, rehabilitation, aerobic training, best practice recommendations, screening, prescription

# Abstract: P020 – THE SCIENCE OF EXERCISE AND NEUROPLASTICITY: IMPLICATIONS FOR REHABILITATION AND PROMOTION OF BRAIN HEALTH

**Published:** 2012-05

**Authors:** M Ploughman

**Affiliations:** Rehabilitation Research, Eastern Health Authority and Memorial University, St. John’s Nfld

**Learning Objectives and Session Content:** Exercise advice to enhance and maintain brain health in healthy people and people with chronic conditions is an emerging role for physiotherapists. The effects of exercise on brain plasticity especially in the area of recovery and rehabilitation is one of the fields in which biomedical science is advancing faster than clinical research. This session synthesizes findings in biomedical and clinical research in the field of neuroplasticity in both the injured and non-injured brain. Research findings will be applied to the challenges in maintaining brain health in the aging brain and in the rehabilitation management of brain injury and chronic movement impairments using three clinically relevant case examples (acute brain injury, secondary progressive MS and chronic pain).

Following the session, attendees will be able to:

1. Understand seminal research in animal models and imaging studies that explains plastic changes in response to normal and abnormal sensorimotor input including exercise,
2. Cite relevant research that supports physiotherapy approaches and 3. Appreciate the gaps in knowledge where more research is required.

**Relevance to Physiotherapy Profession:** As experts in both exercise and neurological recovery, physiotherapists must be able to understand how basic biomedical science supports our practice and also guideneuroscientists to research the questions that are most important for our patients. We realize that exercise influences cardiovascular, musculoskeletal and neurological health and that people with chronic movement impairment also have brain changes in response to abnormal movement. Understanding of the science of neuroplasticity will help us to articulate how our interventions influence brain reorganization and to provide informed exercise advice to promote brain health.

**Target Population:** The session will be particularly relevant for physiotherapists in the field of neuroscience however, since all brains are plastic, the discussion will also be relevant for physiotherapists who are interested in maintaining their own brain health along with those of their patients.

**Description of Supporting Evidence:** Exercise and environmental enrichment increase molecules and proteins linked with synaptic trafficking and dendritic branching in the normal (Neeper 1995), injured (Ohlsson 1995) and aged (Greenough 1999) brain. In people older than 35 years, lifelong physical activity preserves neurocognitive function (Singh-Manoux 2005) and protects against dementia (Podewils 2005). Aerobic fitness is associated with greater gray matter volume in older people (Kramer 2006) and people with multiple sclerosis (Prakesh 2010). Specific intensities, durations and modes of exercise training seem to be required to induce a brain plasticity effect (Smith 2010).

Both skilled limb training (Nudo 1996) and limb disuse (Liepert 1995) cause rapid changes in cortical map representations of the body. After brain injury or stroke, due to temporary increases levels of growth promoting proteins, the brain returns to a more malleable developmental state (Murphy & Corbett 2010) in which timing of rehabilitation intervention becomes critical (Biernaskie 2006). Techniques that may sustain neuroplasticity beyond the ’window of recovery’ such as endurance exercise (Ploughman 2005) and transcranial magnetic stimulation (Yoon 2011) may become more important in the future rehabilitation management of brain injury.

**Description:** The content will be delivered primarily through a lecture format; however relationship to clinical practice will be emphasized. The content is aimed at translating extensive biomedical research in neuroscience to practical approaches to rehabilitation. Three cases (older person with multiple sclerosis, young person with acute brain injury and person with chronic pain) interspersed throughout the talk will stimulate discussion and problem-solving amongst attendees.

**Conclusion:** Physiotherapists have the skills and knowledge to sculpt neuroplasticity in the injured, aged and uninjured brain. Rehabilitation intervention, strategically employed, can influence the neuroplastic changes that lead to functional recovery. This session, by translating biomedical and clinical research in neuroplasticity to clinical practice, will help physiotherapists identify new opportunities to promote brain health in their patients.

**Keywords:** neural plasticity neuroplasticity

# Abstract: P021 – BRIDGING GENERATIONS: TOGETHER WE ARE BETTER!

**Published:** 2012-05

**Authors:** C Evans, B Mori

**Affiliations:** Department of Physical Therapy, University of Toronto, Toronto, Ont

**Learning Objectives and Session Content:** Upon completion of this session, attendees will be able to:

1. Describe unique characteristics attributed to Millennial, Gen X and Baby Boomer generational groups
2. Discuss common values of current student group, the Millenials, and the impact on their behaviours
3. Discuss ways in which generation impacts teaching and learning preferences in the educational and clinical settings
4. Discuss strategies to provide relevant and useful guidance, support and mentorship in the educational and clinical settings

**Relevance to Physiotherapy Profession:** Knowledge of the differences and similarities in values and behaviours among the various generations in the clinical and educational settings will allow managers, leaders, clinical supervisors and mentors to make decisions about how best to support and guide students and younger clinicians to enhance their learning and performance.

**Target Population:** This workshop would be applicable to a broad range of attendees, those working in the public and private sector, individuals in management and leadership positions or individuals who work and interact with others taking active roles in the education, mentorship or supervision of students and young clinicians.

Our small group breakout sessions will provide a range of case scenarios related to learning and professional issues from the perspective of multigenerational colleagues, clients and students.

**Description of Supporting Evidence:** Different generational groups have unique characteristics including their own values and assumptions about learning and workplace behaviours. Generations are shaped by moments and critical events in their formative years, cultural innovations and demographics of their time as well as those of previous generations. Understanding the differences and similarities between generations will allow clinicians and educators to capitalize on the positive traits and adopt strategies to manage differences. A common way to categorize a population is to classify individuals based on the time period in which they were born. Although there are slight variations, in general researchers (Tapscott, 1998; Brown, 2000) agree that the categories include Boomers (1946–1964), Generation X (1965–1982) and Millennials or Net Generation (1982–present).

In most instances, faculty in university and managers in work settings are Boomers, while the student populations and new graduates coming into the work force are Millennials. There is evidence (Tapscott, 1998; Howe, 2003) that points to the different ethical values, workplace and learning preferences of the Millennial generation. Themes related to this generation have reported to be a sense of immediacy, fascination with new technology and innovation, racially and ethnically diverse, free expression, strong views and assumed open access to knowledge and information. These differences are strongly associated with Millennials’ use of technology for communication, networking, seeking out new information, learning and entertainment. Strategies designed to harness these unique characteristics and attributes have been proposed.

According to Brown 2000 and Frand 2000, this group flourishes in experiential learning in which they use trial and error based on immediate feedback to create new solutions to problems. In nursing (Skiba, 2006), strategies have been proposed to accommodate and support young students and young clinicians.

Social connectivity is valued by these individuals who are accustomed to talking and texting to connect with their peers regardless of time or geography. The wired generation is reported to excel in small groups with high level of interaction, collaboration and global engagement where their opinions are an important component of the learning process (Crittenden, 2002).

The new generation of students and young clinicians challenges traditional models of education and supports a shift to a new paradigm for learning and practice that encompasses active learning, collaboration, engagement, social connectivity (Skiba, 2002; Windham, 2005)

**Description:** We anticipate the format of the 2.0 hour interactive workshop would be as follows:

Introduction (5 minutes) Background on Millennials, Gen-Xs, Baby Boomers (Didactic presentation with slides – 15 minutes) Group exercise with three cases (Large group Exercise – 15 minutes) Summary of issues from group exercise (Facilitated group discussion – 10 minutes) Presentation of strategies for supporting younger student clinicians (Didactic presentation slides – 20 minutes) Break Case demonstration and discussion (Case – 10 minutes) Small group breakout session (Cases A-C in small groups – 15 minutes) Debrief each case (Large group discussion – 15 minutes) Independent reflections & Evaluation (Independent work – 15 minutes)

**Conclusion:** By providing this workshop, we hope to enable attendees to gain knowledge of generational characteristics and facilitate the exchange of ideas for practice that support the learning and development of students and young clinicians in educational and workplace settings.

**Keywords:** learning, values, generations

# Abstract: P022 – REVOLUTIONIZING REHABILITATION AND HEALTHY AGING: BRINGING EXERCISE ’TABOOS’ INTO THE MAINSTREAM OF CARE

**Published:** 2012-05

**Authors:** SJ Butcher, SC Webber

**Affiliations:** School of Physical Therapy, University of Saskatchewan, Saskatoon, Sask

**Learning Objectives and Session Content:** Traditionally, in physical therapy practice and rehabilitation methodology, the phrase “start low, and go slow” has represented the basis for initiating exercise training. High-intensity exercise and fast velocity resistance training have usually been reserved for the enhancement of sport performance and have been considered to be “too much” for the average client. Recent evidence, however, has demonstrated the clinical usefulness of these training methods in enhancing functional performance in older adults and chronic disease populations. This presentation will describe these methods, provide current evidence regarding their use in these populations, and give an overview of their clinical application.

1. Compare and contrast traditional resistance training with power (high velocity concentric) training and traditional aerobic training with high-intensity interval training for attaining physiological and functional gains.
2. Provide examples of how power and high-intensity interval training methods may be practically included in exercise programs for older adults and for chronic disease rehabilitation.

**Relevance to Physiotherapy Profession:** This topic will provide an update on the current evidence related to the advanced exercise training modes of muscle power and high intensity interval training. Both methods have shown to be highly useful in achieving gains in high functional performance; which will be of high relevance to therapists.

**Target Population:** Clinicians, researchers, and educators interested in healthy aging and chronic disease rehabilitation.

**Description of Supporting Evidence:** With aging, the ability to generate explosive force decreases at a younger age and at a faster rate than maximal strength generation capabilities. This is important because rapid generation of force is critical (and may be more relevant than maximal strength) for general mobility tasks and when balance is challenged. Cross-sectional studies have demonstrated that power generation (the product of force and velocity) is more strongly associated with functional outcomes than is maximal strength. For these reasons, and because of training specificity, clinicians and researchers should consider including power training (high velocity concentric training) in resistance programs for older adults (with and without chronic disease). The evidence from systematic reviews suggests that power training is feasible for older adults and may be more effective than traditional resistance training at inducing specific neuromuscular adaptations important for functional mobility (e.g., gait speed, chair rise, stair climbing) and balance.

Many chronic conditions (including sedentaryism in aging) cause exercise intolerance which can often significantly impair activities of daily living. Traditional exercise programs effectively incorporate low intensity, aerobic exercise as a mainstay of achieving improvements in health and fitness. Low intensity exercise, however, provides limited gains in functional performance and in physiological cardiovascular adaptation. In addition, low intensity aerobic exercise can only be effective if exercise volume (duration and frequency) is high. High-intensity interval training, on the other hand, is a very time-efficient method of achieving physiological and functional adaptations that are similar to, and sometimes greater, than those achieved by low-intensity exercise. Most importantly, this type of training also tends to be more tolerable than continuous aerobic exercise.

**Description:** The format will be lecture and discussion-based as follows: Introduction and background information – 5 minutes (lecture) Power training – 20 minutes (lecture) High-intensity interval exercise – 20 minutes (lecture) Clinical implications and case discussion – 15 minutes (discussion and questions)

**Conclusion:** The outcome of this presentation will be to enhance the ability of therapists/educators/researchers to apply these lesser utilized, but very important, methods of exercise training. Learners will be able to identify clients who will benefit from these methodologies, and to effectively prescribe simple exercise programs using the same.

**Keywords:** exercise physiology, aging, chronic disease, sedentaryism, power training, high-intensity interval training

# Abstract: P023 – PATIENT CHARACTERISTICS, PRESENTATIONS AND OUTCOME MEASURES FOR PATIENTS WITH PATELLAR INSTABILITY

**Published:** 2012-05

**Authors:** S Kerslake, LA Hiemstra, M Lafave

**Affiliations:** * Banff Sport Medicine, Banff; † Mount Royal University, Calgary, Alta

**Learning Objectives and Session Content:**

1. Provide a review of etiologies and incidence of patella instability
2. Discuss accuracy and reliability of clinical assessment techniques
3. Consider the evidence of current treatment approaches
4. Review outcome measures applied to patella instability

**Relevance to Physiotherapy Profession:** Patients with patellar instability present with a wide range of etiologies and symptoms as well as varied anatomic and neuromuscular characteristics. Similar to shoulder instability many of these patellar instability variables exist on a continuum, but patients tend to cluster toward one end or the other, creating two main subsets of patients.

To appropriately treat and manage these patients requires the ability to accurately differentiate between the two subsets that present with patellar instability. The ability to accurately differentiate these populations will help to guide rehabilitation, return to sport recommendation and determine the need for surgery.

Systematic review of clinical assessment and treatment strategies has identified areas for improvement in patella assessment, including exclusion of frequently used measures due to poor accuracy and reliability.

The ability to practice evidence-based medicine is dependent on the use of disease-specific outcome measures. Unfortunately, there is no validated algorithm to assist with the non-operative and/or operative management of patella instability patients. In addition there are currently no published, patient-reported, outcome measures that are disease-specific for the treatment spectrum of this population.

**Target Population:** This session will be of value to therapists regularly assessing and treating knee injuries.

**Description of Supporting Evidence:** Patellar instability is a common problem with an incidence of 7/100,000 that increases to 30/100,000 in patients under 19 years (Fithian 2004). Patellar dislocation is significant since 40–60% of patients will have ongoing knee symptoms including pain and recurrent instability (Cofield 1977, MacNab 1952, Hawkins 1986). In patients with a history of recurrent patellar instability, Fithian (2004) reported a 6.6 times increased likelihood of additional patella instability episodes over time.

In the last five years, interest in the management of these patients has increased substantially, as evidenced by the significant increase in publications on this topic.

Our ability to practice evidence based medicine is dependent on the availability of valid and reliable assessment techniques and outcome measures that are specific to an injury type or patient population. Although many outcome measures, both general and disease specific, exist for knee conditions, there are currently no published, patient-reported, outcome measures that are disease- specific for the treatment spectrum of patella instability. Smith et al (2008) performed a systematic review of clinical tests and outcome measures for patellar instability and determined “…the sensitivity/ specificity–reliability/ validity of such diagnostic tests and outcome tools remains unclear. On the basis of this finding, the authors suggest that, although the physical examination of the patellofemoral joint is regarded as the cornerstone of the diagnosis of patellar instability, the evidence for the utility of these tests is limited and methodologically flawed. It is therefore reasonable to conclude that there is currently insufficient evidence to support the importance of these tests in clinical practice. Further study is recommended to assess the appropriateness of the identified tests and outcomes so that clinicians can be aware of each tests’ strengths and weaknesses when evaluating this condition.”

**Description:** Presentation including interactive case discussions with audience participation.

**Conclusion:** This presentation plans to review current research and also identify gaps in our knowledge of patellar instability. On completion of this case-based presentation, participants will have an understanding of the various clinical presentations of patella instability, the accuracy of clinical assessments, effectiveness of treatment strategies and applicability of outcome measures.

**Keywords:** patella instability, assessment techniques, outcome measures

# Abstract: P024 – SLEEP – WHAT PHYSICAL THERAPISTS NEED TO KNOW

**Published:** 2012-05

**Authors:** MJ O’Donovan, T Parsons

**Affiliations:** Queen’s University, Kingston, Ont

**Learning Objectives and Session Content:**

Following this session participants will be able to:

1. Incorporate evidence regarding the impact of sleep disruption on their patients’ conditions into physical therapy assessment and treatment plans.
2. Differentiate whether patients may have sleep disturbances (Obstructive Sleep Apnea & Restless Legs Syndrome) that require further specialized assessment.
3. Identify possible Restless Legs Syndrome in patients, educate patients and offer physical therapeutic treatment options.

Session content will consist of the following:

1. Overview of the importance of healthy sleep on general health emphasizing areas relevant to Physical Therapy practice including:
  - Cognition/Memory/Emotion
  - Obesity/Diabetes/ Cardiovascular disease
  - Physical activity & recovery
  - Pain perception & tolerance
2. Definition and discussion of Obstructive Sleep Apnea (OSA) including;
  - Its presentation
  - Adverse health effects focusing on those relevant to Physical Therapy practice
  - How to recognize whether patients may have OSA and who to refer them on to.
3. Definition and discussion of Restless Legs Syndrome (RLS) including:
  - Its presentation
  - Adverse health effects
  - Physiotherapeutic assessment and intervention, including how to differentiate from other common conditions with similar presentation e.g. radiculopathy and neuropathy
4. Practice assessment using validated questionnaires.
5. Summary & Conclusion

**Relevance to Physiotherapy Profession:**

Current research has demonstrated a multitude of important impacts of sleep on general health. As Physical Therapists, we are well aware of the negative impact on sleep of the painful conditions that we treat. Do we know enough, however, about other syndromes that disturb sleep? Both Obstructive Sleep Apnea and Restless Legs Syndrome can cause significant sleep disruptions with major impacts that should be taken into account when assessing and treating these patients. These conditions, if present in our patients, can complicate diagnosis and will impact the outcomes of our treatment plans. Increasing our knowledge base in conditions that disrupt sleep will improve our ability as Physical Therapists to appropriately assess and provide successful treatment to our patient population.

**Target Population:**

This session will attract clinicians, educators and researchers interested in the impact of patients’ sleep cycle disruptions on their physical dysfunction and how to incorporate this knowledge base into best practices.

**Description of Supporting Evidence:**

OVERVIEW

Currently many people in our society are sleeping less than six hours a night. Research shows many important health effects related to sleep and sleep loss. Sleep is necessary for memory consolidation. Studies show that individuals do not recall new information as well if their sleep is disturbed either before or after learning tasks. Chronic sleep deprivation may cause weight gain as it alters the way our bodies process and store carbohydrates, and impacts levels of hormones that affect our appetite. Sleep debt contributes to a greater tendency to fall asleep during the day, which may cause mistakes such as medical errors, air traffic mishaps, and road accidents. Sleep loss results in irritability, impatience, inability to concentrate, and moodiness (Harvard Women’s Health Watch, January 2006). Serious sleep disorders - including Obstructive Sleep Apnea and Restless Legs Syndrome - have been linked to hypertension, increased stress hormone levels, and irregular heartbeat (Manconi et al, 2011).

It well known and documented that individuals with painful conditions often suffer from sleep disturbance and even sleep deprivation if the condition is chronic (Lavigne et al, 2011). As assessing and treating health professionals we must be aware of how this sleep disturbance can impact our patients’ ability to recover or manage their conditions, and how co-existing sleep disturbances of other origin in our patients may affect the success of our treatment plans and the patients’ progress and recovery.

OBSTRUCTIVE SLEEP APNEA

Sleep apnea is as common in our society as diabetes and hypertension. It affects at least 1 in 20 adults. It may cause a variety of symptoms including repeated nighttime waking, daytime sleepiness and non-refreshing sleep. OSA is more common in overweight individuals but can also occur in people of normal body weight. Adverse consequences include an increased risk of cardiovascular and cerebrovascular disease, and an increased incidence of motor vehicle accidents due to excessive sleepiness (Canadian Thoracic Society, 2011).

As primary healthcare professionals we cannot remain unaware of a condition of this significance, which has such broad health effects, and likely impacts many of our patient population. We must become more educated about this condition, be able to identify those individuals who may be yet undiagnosed and refer back to physicians for appropriate follow-up. Additionally, in understanding the impact of Obstructive Sleep Apnea in our patients who are diagnosed and under treatment, we can understand the role it may play in their symptom presentation.

RESTLESS LEGS SYNDROME

Restless Legs Syndrome is a sensory motor condition of unknown etiology that causes unpleasant and/or painful sensations in the legs (occasionally the arms). These sensations are worse in the evenings/night and with rest but can be eased with movement. RLS can seriously impact sleep and quality of life (Ekbom & Ulfberg, 2009). It occurs in approximately 10% of the general population (Michaud et al, 2002), 25-30% of patients with Chronic Obstructive Pulmonary Disease and up to 80% of individuals on Hemodialysis (Unruh, et al, 2004).

Restless Legs Syndrome is known to be under-diagnosed and under-treated (Allen et al, 2011). It is not unlikely that a significant number of physical therapy patients may be experiencing this condition. Common conditions, including neuropathy and radiculopathy, must be differentiated from Restless Legs Syndrome as these often present with similar symptoms (Ondo, 2007).

Exercise has been shown to be beneficial in ameliorating the symptoms of Restless Legs Syndrome in several populations (Sakkas et al, 2008, Aukerman et al, 2006, Esteves et al, 2008, Lettieri & Eliasson, 2008). One study has also shown Infrared Therapy to be of value (Mitchell et al, 2010). Once aware of this condition, Physical Therapists can have an important role in diagnosing Restless Legs Syndrome and providing appropriate education and treatment.

**Description of Session Format:**

This session will be presented in a lecture format, including representative case studies, with opportunity for discussion, questions, and practical assessment practice using validated screening questionnaires as an aid to learning.

**Conclusions and Implications:**

The importance of sleep and how it specifically relates to our clinical practice has not been the subject of a concerted focus for Physical Therapists to date. This educational session will provide physical therapists with an overview of potentially new knowledge of sleep. This knowledge will improve their ability to offer best, evidence-informed assessment and treatment approaches for conditions they currently treat and also conditions of which many may have previously been unaware.

**Keywords:** sleep, best practice, obstructive sleep apnea, restless legs syndrome, physical therapy

# Abstract: D001 – BECOMING INVOLVED IN PHYSIOTHERAPY RESEARCH IN AN INTERNATIONAL CONTEXT: REFLECTIONS FROM RESEARCHERS

**Published:** 2012-05

**Authors:** J Zhan, S Cleaver, M Stevens, D Levac

**Affiliations:** * School of Rehabilitation Science, McMaster University; † Graduate Department of Rehabilitation Science, University of Toronto

**Background:** In a recent guest editorial for the journal Physiotherapy in 2007, Landry and colleagues strongly advocated for the involvement of the physiotherapy profession on an international level to address the effects of disability on poverty and development. This effort would be structured around 3 pillars of activity: “advocacy, research and action.” (p. 234). This diversity of potential roles for physiotherapists (PTs) was empirically supported by a 2007 Physiotherapy Canada study conducted by Alappat and colleagues, wherein PT participants identified their roles in global health as program coordinators, researchers, and direct service providers. Among these, however, the role of the PT researcher is perhaps one of the least understood. Many PT researchers find the prospect of conducting research abroad to be a daunting one. Potential challenges manifest at all stages of the research process and can include coping with funding and resource shortfalls, adapting to unfamiliar practices from partner organizations, surmounting regulatory roadblocks created by local governance, overcoming cultural shock, and more. They may also have an ethical character, such as obtaining informed consent, applying clinical equipoise with respect to different health systems and standards, and appropriately conveying the sometimes- tenuous distinction between clinical treatment and research intervention. However, there are numerous benefits to conducting research abroad as well. Research in the global south presents unique opportunities for cross-cultural learning and community engagement that can enhance the individual PT’s understanding of social determinants of health, improve institutional ties across borders, and enrich host communities. Evidence-based findings may also have significant implications for practice or policy in Canada, and can be used to tackle some of the most pressing domestic health challenges (e.g., improving rural and remote health indicators). Consequently it is important for the profession to demystify the role of the PT researcher through the dissemination of practical information about how to overcome existing roadblocks.

Given the shortage of peer-reviewed literature concerning PT research abroad, our most valuable learning experiences continue to be supplied by experienced colleagues in the field. The purpose of this information session is to expose participants to the lived experiences of PT researchers as they reflect on why they chose to undertake international research, the challenges and benefits of this undertaking, and what the implications of their research will be for practice and policy.

**Relevance to Physiotherapy Practice:** PTs who are interested in conducting research or working abroad may find the first-hand narratives from current and future leading PT researchers insightful and relevant to their professional development goals. Challenges at all levels of the research process, from obtaining ethics approval to enrolling participants, will be highlighted in an accessible manner. In addition, the ensuing discussion of ethical quandaries will provide a forum for participants with prior global health experience to share their own values and insights on ethical practice and translating research into policy. Together it is hoped that a dialogue will emerge that moves past current challenges and addresses the prospects and future directions for Canadian PTs in global health research. Participants will have an opportunity to network with presenters and pose questions during the session.

**Target Audience:** The content of this presentation will appeal to educators, policymakers, physiotherapy students, physiotherapists, and physiotherapy support workers at all stages of their careers interested in physiotherapy research being undertaken in an international context. No prior experience in global health is required; basic knowledge of research terminology is beneficial.

**Summary of Supporting Evidence:** International guidelines for global health research ethics are limited, and sometimes contradictory. Since the publication of the Declaration of Helsinki by the World Medical Association, numerous research codes of ethics have emerged, including those from the European Union and the World Health Organization. However, many report recommendations were incongruent and some were not applicable to the global south, as noted by the Nuffield Council on Bioethics in their report entitled “The ethics of research related to healthcare in developing countries” and the subsequent discussion paper composed with international partner feedback. The Nuffield Council documents included non-binding recommendations that espoused respect for the national health priorities set by the host country, respect for genuine consent and non-participation, and the availability of accessible information sheets and consent forms, among other tenets. Universal guidelines such as these may be taken into consideration in conjunction with national policies and ethics board recommendations to make informed choices in the research process.

Practical guidelines on the subject of PTs working internationally in any capacity are scarce in the physiotherapy literature. However, a small and growing body of qualitative and philosophical studies examining the challenges associated with overseas health service delivery/volunteering have been published with respect to other health care providers (e.g., medicine, nursing, students of various health disciplines). Certain findings – including factors that facilitate and detract from the experience of working abroad, mechanisms of coping, and personal characteristics that are important to this work - may be generalizable to the case of the PT researcher.

**Description of Session Format:** After a short introduction, 2–3 presenters will discuss their experiences in varying stages of the research process, in different parts of the world. Sessions will be moderated and the audience is encouraged to pose questions and provide their own insights.

**Session Objectives:** Upon completion of this presentation, participants will:

1. Be familiar with the role of Canadian physiotherapists in undertaking research in international settings, ethical regulations and process
2. Understand the benefits, challenges, and ethical dilemmas involved in international research
3. Be exposed to the insights and experiences of physiotherapy researchers undertaking research projects abroad

**Keywords:** physiotherapy research, international health, global health, research ethics, professional identity

# Abstract: D002 – STEM CELL THERAPY IN CEREBRAL PALSY: HOW TO EXPLAIN IT TO FAMILIES

**Published:** 2012-05

**Background:** This knowledge exchange workshop emerged from requests by division members to provide them with evidence- based information on new technologies that are emerging for clinical management of children with cerebral palsy. A specific interest regards the current state of research evidence for stem cell therapy. Increasingly, paediatric physical therapists are being asked by families if this is an option for their child. Providing objective answers is often challenging, as this field of neuroscience is ’fast changing’ and evaluating the ’credibility’ of web-based information is difficult to ascertain, particularly from international clinics that are currently promoting and providing stem cell therapy for this paediatric clinical population. Cerebral palsy is an umbrella term that describes a heterogeneous group of non-progressive injuries to the brain1 resulting from a lack of blood and oxygen to the brain during pre-natal development of around the time of birth2. The white matter cells of the brain are most susceptible to the effects of this hypoxic-ischemic insult occurring early in brain development3, and disrupting the ability of the neurons in the brain to communicate effectively with each other4. Stem cell therapy aims to replace these cells4. While various international clinics currently provide stem cell therapy for children with cerebral palsy, there is currently no controlled evidence regarding their effectiveness, nor that they provide clinically meaningful benefits in humans, including children with cerebral palsy5.

*****Rosenbaum, P et al. Dev Med Child Neurol Suppl 2007; 109:8-14;
2. Bartley J, Carroll JE. Expert Opin Biol Ther 2003; 3:541-549.
3. Levison SW et al, Dev Neurosci 2001; 23:234-247;
4. Ruff C et al. http//www.childhooddisability.ca/cdc_en/newsletter.php (accessed November 17, 2011);
5. Titomanlio L, et al Ann Neurol 2011; DOI: 10.1002/ana.22518

**Relevance to Physiotherapy Practice:** A foundational role of paediatric physiotherapists is to act as ’knowledge brokers’ for families, providing objective, evidence-based information to guide their decisions regarding health care for their child. Many families are looking for answers to their questions on the ’reality’ of stem cell therapy for cerebral palsy1. For emerging fields of practice, as for example stem cell therapy, finding credible information for families is challenging, and interpretation of findings can be difficult to translate to family-centered language. This knowledge exchange workshop will provide participants with a credible landscape on the current state of knowledge in stem cell therapy, and discuss future directions in this rapidly expanding field of neuroscience as they relate to cerebral palsy. The workshop will also provide ’tips’ to guide participants in evaluating the quality of research evidence and to find credible sources of web-based information on emerging technologies and therapy approaches in clinical paediatrics and enhance information literacy skills necessary to be effective knowledge brokers.

1. Ruff C et al. http://www.childhooddisability.ca/cdc_en/newsletter.php (accessed November 17, 2011)

**Target Audience:** The topic of stem cell therapy in paediatrics is of interest to a wide audience, including all clinicians working with children and their families. Developing skill in being a knowledge broker is also of relevance to all clinicians, independent of their field of practice. The ability to identify, evaluate and share evidence-based information is one of the cornerstones of scholarly practice.

**Summary of Supporting Evidence:** The simple act of typing ’stem cell therapy for cerebral palsy’ in the Google search engine produces a number of clinics promoting the use of stem cells to ’cure’ cerebral palsy or to significantly ameliorate its outcomes. This has prompted Dr Fehlings, MD, PhD, Medical Director of the Krembil Neuroscience Program at the University Health Network, to adopt the term ’stem cell tourism’ and to recommend that families consider stem cell treatment within the context of a registered clinical trial. There is encouraging basic science support regarding the use of stem cells for the improving the white matter integrity in the developing brain having sustained a hypoxic-ischemic insult, and, ultimately, functional outcomes1,2. The challenges into translating these findings to the clinical setting are comprehensively outlined in a recent article by Titomanlio et al2. Currently, the majority of the evidence has been provided in animal models and the translation of these findings to paediatrics remains to be evaluated through controlled studies.

Despite these challenges, families are seeking information and there is growing interest in accessing stem cell therapy for their child with cerebral palsy. Paediatric physiotherapists play a critical role in this decision-making by providing families with the necessary research information and evidence to guide their decisions. However, research has demonstrated that therapists do not routinely integrate new research evidence within their own clinical decision-making3,4,5, make it less likely that they would be able to dedicate the time needed to identify, evaluate and synthesize information in a way that would be meaningful for families. Providing therapists with a grounded approach to access and evaluate information on novel therapies, in this case stem cell therapy, could support therapists to actively engaging as knowledge brokers within a family-centered approach.

1. Bartley J, Carroll JE. Expert Opin Biol Ther 2003; 3:541-549;
2. Titomanlio L, et al Ann Neurol 2011; DOI: 10.1002/ana.22518;
3. Jette DU et al. Phys Ther 2003; 83:786-805;
4. Schreiberr J. et al Pediatr Phys Ther 2003; 20:292-302;
5. Rivard LM et al Phys Ther 2010; 90:1580-1590.

**Description of Session Format:** This workshop will integrate lecture by content experts with active engagement of participants in dialogue regarding the resources that they would need to support them in becoming knowledge brokers to families. The development of an action plan between participants and the Paediatric Division will conclude the workshop, and will provide the Division with necessary information to develop educational materials that are relevant to clinicians working with this paediatric clinical population.

**Session Objectives:** Upon completion of the session, participants will be able to:

1. Discern objective and high-quality information on stem cell therapy for cerebral palsy, including credible websites, that they can integrate into their communication strategies with families;
2. Engage in meaningful discussion regarding the current opportunities for stem cell therapy in paediatrics, the scientific challenges that need to resolved, and the future ’reality’ for stem cell therapy in the management of cerebral palsy;
3. Appreciate the expanding role of paediatric physiotherapists as knowledge brokers, providing families with the objective, evidence-based information that they need to make the ’best’ decisions on the health care for their child.

**Keywords:** cerebral palsy, stem cell therapy, robotic technology, knowledge translation

# Abstract: D003 – FEAR-AVOIDANCE AND CHRONIC PAIN: MODELS AND TREATMENT IMPLICATIONS

**Published:** 2012-05

**Authors:** Gordon Asmundson

**Affiliations:** Faculty of Arts, University of Regina, Regina, Sask

**Background:** The fear-avoidance model of chronic musculoskeletal pain, as postulated by Vlaeyen and Linton (2000), has stimulated extensive research and the development of a variety of interventions focused on reducing pain-related fear, avoidance behaviour, and functional disability in patients with various types of chronic pain. Although there is considerable evidence supporting the Vlaeyen and Linton (2000) fear-avoidance model, oversight of potentially important factors and inconsistent findings regarding postulated pathways have led to proposed model revisions (e.g., Asmundson, Norton, & Vlaeyen, 2004; Hassenbring & Verbunt, 2010; Norton & Asmundson, 2003; Pincus et al., 2006). The purpose of this presentation is to provide an overview of the original Vlaeyen and Linton (2000) fear-avoidance model, to highlight key model revisions, and to summarize research that directly analyzes the pathways proposed in the original and revised models. The growing body of evidence emerging from analysis of proposed fear-avoidance pathways using structural equation modeling is generally supportive of the original model as well as various proposed revisions. Additional evaluation using structural equation modeling may lead to a formulation of the fear-avoidance model that balances parsimony with heuristic value. Clinical implications for physiotherapists who treat patients with various forms of acute and chronic pain will be discussed and areas of for future research highlighted

**Relevance to Physiotherapy Practice:** A primary role of physiotherapists in treating acute musculoskeletal pain and injury is to facilitate recovery and thereby prevent chronic pain or disability. Understanding the process and key predictors of chronic pain and disability is of paramount importance for assessment of the injured person and development of effective and efficient treatment plans. This session will provide physiotherapists a sound foundation in understanding this process from a cognitive/behavioural perspective and will arm them with tools to identify and treat important risk factors.

**Session Objectives:** Objectives:

1. To introduce and highlight key postulates of the Vlaeyen and Linton (2000) formulation of the fear-avoidance model of chronic pain
2. To review literature supporting the original fear-avoidance model as well as its various revisions
3. To highlight treatment implications and considerations for physiotherapists.

**Keywords:** pain, fear, cognition, behaviour, anxiety, post-traumatic stress

# Abstract: D004 – ELECTRONIC MEDICAL RECORDS (EMR): MEANINGFUL USE IN PHYSIOTHERAPY PRACTICE

**Published:** 2012-05

**Authors:** D Yardley

**Affiliations:** Private Practice Division, Canadian Physiotherapy Association

**Background:** Electronic Medical Records (EMR) is an enabling technology for clinicians’ practices to pursue quality improvement in potentially powerful ways. Quality improvement in a clinic depends heavily on the clinicians’ use of EMR—and not paper—for day-to- day practice (Rozenblum et al., 2011). The transition from a paper-based health record to an EMR must be addressed and managed on many different and complex levels: administratively, financially, culturally, legislatively, technologically, and environmentally (Boonstra, 2010). Despite the suggested positive effects of EMR usage in clinical practice, the adoption rate of EMR is still low and meets resistance from healthcare professionals given the associated complexities involved. This presentation will provide guidance and practical steps for managing the transition from paper to EMR. The “meaningful use” rule strikes a balance between acknowledging the importance of adopting EMR to improve clinical outcomes and recognizing the challenges that adoption will pose to healthcare providers (Jha, 2011). Providers may face a range of barriers when they approach EMR implementation. Potential barriers and suggested intervention strategies will be highlighted to act as a reference to facilitate the integration process.

Providers/organizations that do not have the knowledge or resources to properly evaluate EMR may be at risk of making uninformed purchase decisions favouring products promoting the capture of “meaningful use” rather than usability or legislative regulation. Many valuable lessons have emerged in recent years that are assisting healthcare providers and organizations ease the transition into a workable and practical electronic environment. The presentation will identify the true potential of EMR to improve the safety, quality, and efficiency of patient care. The operability of electronic records can position healthcare providers to deliver patients the best possible care by analyzing the value of treatment decisions and to allow us to build practice guidelines. Furthermore, the ability to share information improves communication between providers, which can result in enhanced coordination and continuity of patient care.

**Relevance to Physiotherapy Practice:** Physiotherapists adoption of EMR appears to lag behind that of physician practices reported in the literature. In order to keep pace with other professionals and enhance our role within the healthcare team across the continuum of care, physiotherapists need to have a better understanding of EMR (Paschal, 2011). With adequate leadership, consultation, staff training, software and hardware, policies and procedures, and funding EMR implementation can be simplified. A well-designed EMR that satisfies legislative standards for privacy and competencies can assist physiotherapists in improving operational efficiency and clinical decision-making/outcomes, avoiding preventable errors, contributing to research (data collection), and improving communication and reporting. Common complaints surrounding implementation centre around cost and initial lost productivity, but the favourable elements are in many cases immeasurable (Dunn, 2007). More important to consider is how the adoption of EMR can position physiotherapists as leaders within the rehabilitation network and augment our acceptance and expanding role within primary care. With the positive impact it has on patients’ care, and the potential to advance the physiotherapy profession, EMR offers profitable solutions from a business perspective. A carefully selected EMR can translate into a positive return on investment, enhanced patient satisfaction, and a practice/organization with growing employment opportunities.

**Target Audience:** This session will be of interest to a broad range of healthcare professionals including clinicians, public and private administrators, professional leaders, and researchers with an interest in EMR.

**Summary of Supporting Evidence:** With the effort to change health care delivery, research around the adoption of EMR has primarily been focused on physician practices. The evidence from systematic reviews suggests that the implementation of EMR into practice results in improved patient outcomes and clinical efficiencies. Despite the positive effects of EMR usage and financial incentives, physicians continue to remain slow and resistant to transition to electronic records due to funding barriers, concurrent regulatory compliance timelines, competing technical priorities, strained human resources, and the lack of industry education (AHIMA, 2010). A systematic literature review highlighting a multitude of barriers to physicians accepting EMRs have also suggested interventions that could be helpful for providers to overcome these challenges. However, it would be wrong to conclude that there is a “one way fits all” route to EMR integration. As such, the knowledge gained from the physician research should merely assist in the evaluation of appropriate EMR systems for physiotherapy practice. With the growing awareness towards a “paperless” practice, The American Physical Therapy Association, and its affiliated members, have produced some recent research on EMR implementation. Evidence suggests that physiotherapists presently lack the ability and knowledge to evaluate, consult and manage an EMR driven practice. To avoid extrapolating from physician practices/organizations, research is needed to determine the effects of EMR adoption and use on patient outcomes, patient satisfaction, clinical efficiencies and safety within physical therapy practices. Furthermore, the presentation will drive future research questions associated with data collection and practice guidelines, leadership and expanding roles within primary care, business development and return on investment.

**Description of Session Format:** This session will be a lecture format and will include a question and answer period to provide participants an opportunity to share their perspectives and experiences with EMR.

**Session Objectives:** At the end of this session, participants will be able to:

1. Discuss how the elements of EMR may be adopted into physiotherapy practice and understand the critical factors necessary to improve quality outcomes;
2. Identify, categorize, and analyze barriers associated with EMR in practice;
3. Understand the advantages of electronic charting (“going-paperless”) and how it is the fundamental concept to improve the quality and efficiency of patient care; and
4. Develop strategies to manage the transition from paper to EMR.

**Keywords:** electronic charting; electronic health records (EHR); electronic medical record (EMR); health information technology (HIT); paperless; physiotherapy practice; practice management software; return on investment; technology

# Abstract: D005 – HOW TO USE EDUCATION & COMMUNICATION TO ENHANCE THE PATIENT-PHYSIOTHERAPIST RELATIONSHIP AND GROW YOUR BUSINESS

**Published:** 2012-05

**Authors:** S Funk,

**Background:** Successful private physiotherapy practice owners know that the strongest clinicians do not always get the strongest results and that the physiotherapist-patient relationship appears to have a positive effect on treatment outcome. This therapeutic alliance is considered by some, to be central to the therapeutic process. Yet, further research is required to understand the impact of this therapeutic relationship in the private practice setting and how it is influenced when patient financial issues are involved.

This high energy, yet informative session will explore a few of the key factors responsible for enhancing the physiotherapist-patient relationship through effective patient education and clear communication to help grow a successful business and strong referral base.

Additionally, one key factor directly responsible for business growth is patient compliance. Patient compliance and subsequent program adherence is one of the most complicated and important issues facing physical therapists and clinics and is influenced by many factors ranging from practitioner-patient relationship to financial barriers. Conducting efficient and effective patient education is a vital component of client/patient centered care. Consequently, patient compliance and adherence are outcomes of effective patient teaching and can ultimately enhance the patient experience and clinical outcome, leading to a growing referral base and marketing strategy.

Although barriers to effective communication and education will exist for various reasons efforts can be made to bridge the gaps and enhance the patient experience. A recent reader survey by a reputable journal reported that clinicians ranked providing practical information to patients higher than any other new feature that the journal might add to the print and online. Technology advances and the internet have made significant advances in recent years to help clinicians communicate current research developments to their patients in a format that is not only practical, but also informative and effective.

**Relevance to Physiotherapy Practice:** Physiotherapists have been taught to integrate patient education into their treatment programs. However, the components of a strong patient education program and effective communication skills may not be understood and utilized by all physiotherapists when interacting with patients and referral sources. Furthermore, health literacy barriers, patient financial issues, time constraints, limited fee structures, and rapid developments in musculoskeletal rehabilitation may complicate the picture and impede implementation. In order to keep abreast with best practice developments and enhance patient outcome, grow the referral base, and ensure patient program adherence, physiotherapists must learn what patients expect and want from their health care providers. The presenter will provide practical tips and simple strategies that can be easily and economically be integrated into daily practice to improve patient satisfaction and outcomes. Not to be forgotten is the referral source, a critical partner, which we also have the opportunity to educate and communicate with during this process. Education and communication is often under-utilized by many private physiotherapy clinics and can be used as part of the marketing plan of the business. Attendees will be provided with some easily implemented strategies to utilize with each patient to enhance the patient- referral source relationship and grow the referral base further.

**Target Audience:** A prior level of exposure to this topic is not required to successfully follow this session. This session is targeted to those in the health field interested in furthering their knowledge in the area of effective communication and education of patients and referral sources. This includes physiotherapists in both public and private practice, who perform clinical care or administrative duties, employees and independent contractors.

**Summary of Supporting Evidence:** Patient compliance and overall treatment success rely on many factors. This presentation will highlight the growing evidence from systematic reviews, studies and discussion papers that indicate that the therapeutic alliance between the therapist and patient plays a vital role in ensuring our patient’s maximum health and wellbeing. Author Donna Falvo maintains that effective education is the key to building the therapist-patient relationship in order to increase patient compliance and adherence to treatment. The therapist’s ability to build trust and foster a partnership with the patient, and to identify and overcome barriers are as important as the therapist’s skill set and knowledge in ensuring a positive outcome for the patient. A number of studies are outlining the importance of communication to a successful relationship. Poor communication can have very negative effects on the therapist-client alliance and has been shown to be the root cause of malpractice claims. Author Malcolm Gladwell, author of Outliers: The Story of Success, acknowledges how important demonstrations of caring and compassion can be in the medical world. Citing research from Vincent, Young, and Phillips (1994), he points out that overwhelming numbers of people who suffer an injury due to negligence of a doctor never file a malpractice suit at all because of the bond they had with the doctor. Inversely, good communication with the patient will foster a positive relationship. For patient-centered care (PCC) to work, major behaviour change is required of both the therapist and the patient. As described in discussion papers by Stephen Lewis and by the Canadian Medical Association, there are barriers such as psychology, social norms, and price considerations, which come into play when adopting a patient-centered care (PCC) model. This presentation will show that the benefits to a PCC model of care far outweigh the costs and therapists should strive to incorporate this model in their practice.

**Description of Session Format:** This session will be in lecture format and will include a question and answer period at the end of the presentation that will provide participants the opportunity to share their perspectives and experiences and pose questions to the session presenter.

**Session Objectives:** Upon completion of this session, participants will be able to:

1. understand that a patient-physiotherapist relationship exists and how this impacts both positively and negatively on clinical outcomes;
2. apply practical strategies to strengthen their current communication and educational methods with both patients and referral sources; resulting in improved patient compliance, adherence, satisfaction, and subsequent business growth;
3. integrate patient centered approach to care and biopsychosocial model of care into the private practice setting.

**Keywords:** education, effective and clear communication, business growth, referral sources, clinical outcomes, profit, patient compliance, adherence, physiotherapist-patient relationship, client directed care, trust, likability factor, health literacy, referral network

# Abstract: D006 – BE IT RESOLVED THAT A ’GLOBAL HEALTH’ EXPERIENCE CAN BE FOUND IN CANADA: A STRUCTURED DEBATE

**Published:** 2012-05

**Authors:** D Levac, M Fricke, E Yeung

**Affiliations:** * School of Rehabilitation Sciences, McMaster University, Hamilton, Ont; † Department of Physical Therapy, University of Manitoba, Winnipeg, Man; ‡ Department of Physical Therapy, University of Toronto, Toronto, Ont

**Background:** Participation in international health initiatives is of interest to many Canadian physiotherapists. There are many areas of the world that have substantial healthcare and rehabilitation needs but have few local services or professionals to meet these needs. However, practical constraints prevent most physiotherapists from traveling abroad to volunteer their skills with marginalized populations in low and middle income countries (LMICs).

As global borders become increasingly porous, healthcare providers are caring for a growing number of people from cultures other than their own. Whether in a rural clinic in another country, or a major hospital in urban Canada, cultural differences can impact on the provision of healthcare. The 2006 Census revealed more than 200 different ethnic origins in the Canadian population, with 16.2% of the total population of Canada belonging to a visible minority. Canada has the highest per capita immigration rate in the world and by 2031, almost one-half of the Canadian population over the age of 15 will have been born abroad. The 2006 Census showed that 83.9% of the immigrants who arrived between 2001 and 2006 were born in regions other than Europe (ref).

Indeed, socioeconomic disparities faced by marginalized populations in Canada are often similar to those of LMICs. Recent immigrants may include those from regions of the world with poor or nonexistent health care and may be refugees who have experienced war, famine or other physical and mental health challenges. Importantly, Aboriginal peoples have well-documented health inequities arising from a history of colonization impacting social and economic determinants of health. Working with these clients, addressing these inequities and facing these potential challenges offers an opportunity for physiotherapists to become involved in ’closer-to-home’ health equity initiatives. Physiotherapists interested in these issues are supported by relevant CPA position statements outlining the role for physiotherapy in addressing population health issues within Canadian clinical practice.

Cultural competence is the ability to understand and to work effectively with patients whose beliefs, values, and histories (i.e. culture) are different from one’s own. The idea that cultural competence is an ongoing process suggests that it is a skill that one can continually refine, not an endpoint that one reaches and subsequently remains stagnant. Therefore, regardless of the length of time one has been practising, or the amount of international or cross-cultural experience one has, this is a skill that can and should be kept at the forefront of one’s mind.

The purpose of this session is:

1. to encourage awareness, discussion and debate about the relevance of global health issues to PTs practising in Canada and
2. support the creation of practice environments that encourage culturally competent care.

**Relevance to Physiotherapy Practice:** CPA position statements encourage physiotherapists to be advocates for the health, mobility, and independence of all Canadians and to consider political, ethical, and social issues impacting patient welfare within their practice. As rehabilitation professionals, we are well positioned to take a lead in providing culturally competent care. The attention that physiotherapists pay to client education, as well as the need to determine what is meaningful to each patient (for the development of exercises and activities that are relevant to the client, for example) require a degree of sensitivity to the client’s cultural background. Thus, effective physiotherapists have likely already been using a relatively culturally competent approach. Increasing our understanding of these issues and debating their relevance to practice in Canada will serve to enhance discussion and encourage therapists to become involved in global health issues both in Canada and abroad.

**Target Audience:** The content of this presentation will appeal to educators, policy-makers, physiotherapy students, physiotherapists, and physiotherapy support workers at all levels of their careers, who have an interest in cultural competence and the role of physiotherapy as it pertains to global health issues both in Canada and abroad.

**Summary of Supporting Evidence:** The skills of Canadian physiotherapists are in great demand in developing countries. Qualitative research has identified that Canadian physiotherapists have numerous strengths that are valuable for international health work, such as strong clinical, communication and teaching skills. Nonetheless, this research has also identified weaknesses such as unrealistic expectations from physiotherapists involved in international health.

The CPA advocates for the health, mobility and independence of all Canadians. The position statements on the determinants of health and population health suggest that the profession has a role in advocating for healthy public policies. Physiotherapists are able to address the determinants of health in their daily professional practice and are competent in identifying risk factors for disease and disability in individuals and their environment. Therefore, physiotherapists can, and should integrate a population health approach in their practice by collaborating to develop interventions that address population health needs.

The World Confederation for Physical Therapy (WCPT) includes a section on cultural competences for physiotherapists in their position statement for the standards of practice. These include “The physical therapist acquires skills to better understand people from differing cultures in order to achieve the best possible health outcomes” and “Physical therapy is planned and delivered in a way that respects cultural values, requirements and variations.” (World Confederation for Physical Therapy 2007)

**Description of Session Format:** This will be a structured debate session in which two experts will represent opposing sides of the issue. A moderator will facilitate the debate according to accepted debate process and encourage a discussion and question period with the audience.

The International Health Division aims to promote discussion about the role of physiotherapists in global health and human rights issues in Canada and abroad. Many PTs want to be involved and feel compelled to use their skills to address health issues in under-resourced areas, but are unable to travel. This session will explore how a ’global health’ experience might be had in Canada or whether it is unique to the experience of working or volunteering overseas. This session will increase physiotherapists’ awareness of typical ’global health’ issues within their practice in Canada. It will provide opportunities for participants to learn from the panelists and from each other by providing a forum for discussion and networking.

**Session Objectives:** Upon completion of this presentation, participants will:

1. Have an understanding of the needs in developing countries, the elements of international health, and how these relate to physiotherapy practice
2. Be informed as to the health disparities experienced by marginalized urban and rural populations in Canada, including Aboriginal people and recent immigrants
3. Be aware of some critical features of cultural competency as they relate to physiotherapy practice
4. Have strategies with which to become involved in international health initiatives in Canada or abroad

**Keywords:** global health, cultural competence, physiotherapy practice

# Abstract: D007 – WHAT EVERY PHYSIOTHERAPIST NEEDS TO BE A LEADER IN TODAY’S CHALLENGING HEALTH CARE ENVIRONMENT: LEADERSHIP DIVISION WORKSHOP

**Published:** 2012-05

**Authors:** L Sinclair, Lowery C Damp

**Affiliations:** * Physical Therapy Faculty of Medicine and; † Centre for Interprofessional Education University of Toronto, Toronto, Ont; ‡ Leadership Division, Canadian Physiotherapy Association

**Background:** Opportunities for physiotherapists to take on leadership roles are diminishing in publicly funded institutions. Private practice clinicians are increasingly required to act as leaders in their business of health care. As the pool of leaders in physiotherapy decreases, the Leadership Division is creating a Leadership Education series to help grow the leadership competencies of the profession.

The vision of the Leadership Division is to build curriculum of core competencies in professional self-awareness, modeling leadership behaviours and acknowledging qualities in others within the profession, and the health care system through networking, communication, education and research. Using a well-developed Logic Model for Leadership Education Series, the division will help to provide resources to physiotherapists to improve their leadership skills and help physiotherapists move into leadership positions.

The Leadership Division supported a team of final year physiotherapist Master students from the University of Toronto in the development and dissemination of a questionnaire on the self- identification of leadership characteristics from July 2010 through June 2011. This survey received over 1800 respondents from the Canadian Physiotherapy Association community and results are being used to inform the subsequent curriculum.

From Sept 2010 through June 2011, the Leadership Division Executive designed a project charter and in July 2011 hired a consultant with expertise in professional curriculum and the development of leadership skills. This innovative curricular program based on current evidence, literature and best practices in leadership will be shared at Congress 2012.

**Relevance to Physiotherapy Practice:** It is critically important for the profession of physiotherapy to create a pool of physiotherapists with core leadership competencies. The Leadership Division is excited to share with you a new program that will assist aspiring physiotherapists to move into leadership positions while developing their key leadership skills. This program will also help current physiotherapy leaders further develop their skills and thus meet their leadership challenges.

**Target Audience:** This session will be of interest to a broad range of professionals including clinicians, managers, professional leaders, educators, and researchers interested in developing leadership skills or those who wish to further develop their skills and the application to all areas of the health care system.

**Summary of Supporting Evidence:** Many professionals in the health care system are challenged to continue to grow and lead in the current realities of competing priorities and fiscal restraints. The profession of physiotherapy has made strong recent resources available to help all physiotherapists identify both roles and competencies for effective practice in Canada. The National Physiotherapy Advisory Group, and partners, published the Essential Competency Profile for Physiotherapists in Canada in October 2009. This profile describes the essential competencies required by physiotherapists in Canada at the beginning and throughout their career and provides guidance on how to build competencies over time. The framework for the Profile was adapted from the Royal College of Physicians and Surgeons of Canada Competency Framework for Physicians (the CanMEDS Roles). Throughout all seven roles for both professions, (Expert, Communicator, Collaborator, Manager, Advocate, Scholarly Practitioner and Professional) is the need for leadership knowledge, skills and behaviours.

Leadership and management training are one way that can help support life-long learning for professionals as needed in the quickly changing nature of health care practice environments. Hemmer et al (2007) and Stoller et al (2004), describe the leadership curriculum and training programs used for medical directors and residents. The concept of leadership from the perspective of physiotherapists in Canada has been recently surveyed by a group of final year physiotherapy Master students from the University of Toronto. These survey results will also be shared at Congress 2012 and support the need for physiotherapy specific leadership programs.

**Description of Session Format:** This session will be in a 60 minute workshop format. Participants will first learn, in lecture format, about the Leadership Division’s curriculum and core competency development and then will share examples and applications in an interactive small and large group discussion.

**Session Objectives:** Upon completion of this session, participants will be able to:

1. Discuss leadership competencies for the profession of physiotherapy;
2. Identify key considerations to assist aspiring physiotherapists to move into leadership positions and that can help current leaders further develop their leadership skills; and
3. Provide examples of how leadership competencies may be applied to their own practice and organizational setting.

**Keywords:** leadership, competencies, education, curriculum

## References

[B1] National Physiotherapy Advisory Group (2009). Essential Competency Profile for Physiotherapists in Canada.

[B2] Hemmer PR, Karon BS, Hernandez JS, Cuthbert C, Fidler ME, Tazelaar HD (2007). Leadership and management training for residents and fellows: a curriculum for future medical directors. Archives of Pathology and Laboratory Medicine.

[B3] Stoller JK, Rose M, Lee R, Dolgan C, Hoogwerf BJ (2004). Teambuilding and leadership training in an internal medicine residency training program. Journal of General Internal Medicine.

[B4] Frank JR, Jabbour M (2005). Report of the CanMEDS Phase IV Working Groups.

# Abstract: D008 – BALANCE AND FALLS IN CHRONIC OBSTRUCTIVE PULMONARY DISEASE

**Published:** 2012-05

**Authors:** L Denehy

**Affiliations:** Department of Physiotherapy Melbourne School of Health Sciences,The University of Melbourne, Australia

**Background:** Studies investigating the incidence of falls in patients with COPD are lacking in the literature (Roig et al 2009) and most data available regarding the frequency of falls in COPD have been collected using retrospective study designs (Beauchamp et al 2009; Lawlor et al 2003; Hellstom et al 2003) ] The use of prospective records of falls would accurately identify a fall risk profile for the COPD population. By identifying the fall risk profile in COPD, clinicians may be able to design effective preventive strategies.

**Relevance to Physiotherapy Practice:** This topic is novel and as yet under-researched. There is a theortical framework that supports the notion of poorer balance and increased falls risk in COPD, and there is strong evidence for the role of rehabilitation in these patients. Given there is also some evidence for increased falls in this population, physiotherapists working in all areas need to be aware of this and knowledgeable about incorporating assessment and treatment strategies as a component of their overall treatment plan.

**Target Audience:** Physiotherapists working with individuals with COPD, in both acute and rehabilitation settings. Additionally those physiotherapists working in balance and falls areas may be interested in the outcome measures we are using.

**Summary of Supporting Evidence:** Impaired balance performance is associated with an increased risk of falls amongst older healthy adults (Graafmans 1996), and this risk may be greater in patients with COPD due to its extra- pulmonary consequences, including peripheral muscle weakness and decreased levels of physical activity. Balance assessment is recommended within initial screening to identify elderly individuals who need a multifactorial falls risk assessment (American Geriatrics Society 2011). However, balance performance and self-efficacy assessments are not currently part of the management of COPD (Ries 2007) and there is no recognised specific balance performance test currently recommended for patients with chronic respiratory conditions. This analysis would inform health professionals about suitable tests of balance for people with COPD and help to identify those individuals who may be at risk of falling. An acute exacerbation of COPD may alter balance performance in these patients. During an exacerbation, deficits in peripheral muscle stregnth, physical activity and increased levels of systemic inflamation have been reported in these patients (Spruit et al 2003; Pitta et al 2006; Bathoorn 2009]. These factors may further affect balance performance in COPD. By assessing balance outcomes during an exacerbation of COPD we could determine whether balance performance differs across different clinical states in these patients.

**Description of Session Format:** Lecture

**Session Objectives:**

1. To understand the theory regarding balance and falls in COPD
2. To understand the possible reasons for reduced balance and increased falls risk in COPD
3. To identify specific outcomes for use in measuring balance and falls outcomes in COPD
4. To integrate this knowledge for application to a variety of clinical scenarios

**Keywords:** balance, falls, COPD

# Abstract: D009 – UTILIZATION OF EVIDENCE-BASED PHYSIOTHERAPY ASSESSMENT AND TREATMENTS FOR SELECT NEUROMUSCULOSKELETAL CONDITIONS IN ANIMALS

**Published:** 2012-05

**Authors:** T Ziegler, D LaRocque, L Clarke

**Affiliations:** * Canine Rehab Saskatoon, Saskatoon, Sask; † Furry Friends Animal Rehab, Stony Plain, Alta; ‡ Four Paws Rehabilitation, Grandora, Sask

**Background:** Comprehension and best utilization of skills, evidence, and methodology are important factors to applying

rehabilitative sciences to any patient when challenged with novel conditions or pathologies. Animal rehab is merely the application of these sciences in new ways. It is critical for the animal rehab therapist to understand animal pathologies and translate physiotherapy scientific literature in order to develop best practices for assessment and treatment of animal patients and for the development appropriate plans of care.

**Relevance to Physiotherapy Practice:** Animal rehab continues to be an emerging area of practice for physiotherapists. Those who are not engaged in animal practice may think that physiotherapy skills are specific to human patients. It is important to recognize however, that the basic sciences, systematic methodology, and evidence-informed guidelines (for assessment and treatment), which are utilized in human practice, can also be applied to animal patients. The topics selected: equine caudal cervical spondylomyelopathy; conservative management of canine hip dysplasia; iliopsoas strain management in dogs, are all common conditions. Animal rehab practitioners would benefit from an awareness of these pathologies and an understanding of how to plan treatment accordingly.

**Target Audience:** This presentation would be of interest to any physiotherapist with an interest in practising canine or equine rehabilitation, or physiotherapists merely interested in unique ways to implement physiotherapy evidence and skills into practice.

**Summary of Supporting Evidence:** Very little exists in the literature as it pertains to physical rehabilitation of specific animal conditions. Veterinary literature points towards pathology descriptives, however in order to formulate rehabilitation plans for animal patients with said pathologies, the animal rehab practitioner must look to human evidence for guidance with both assessment and treatment in particular.

Soft tissue injuries are an under-diagnosed cause of lameness in dogs. While veterinary literature does recognize and report their existence, human physiotherapy assessment techniques champion the practitioner’s ability to identify soft tissue injuries consistently and reproducibly. Evidence-based guidance (via systematic reviews) for non-surgical treatment of soft tissue injuries is entirely and extensively rooted in human literature.

Equine caudal cervical spondylomyelopathy is a condition resulting in spinal cord compression characterized by weakness and ataxia in all four limbs. Proper assessment for such cases requires a thorough understanding of the cervical mechanics and how to test the nervous system in a horse. Descriptive and retrospective studies predominate the literature on this equine pathology. Canine hip dysplasia is a debilitating condition, characterized by developmental laxity in the coxo-femoral joint, which progresses to an early advancement of osteoarthritis as the dog ages. Veterinary literature expounds upon either surgical or pharmaceutical interventions for this condition. However, evidence-informed treatment (via reviews, randomized controlled trials, and case reports/series) of human hip osteoarthritis is useful and effective in guiding physical rehabilitation for these cases in dogs.

**Description of Session Format:** This session will be a 1-hour lecture format panel presentation, utilizing case studies and allowing for questions & answers of the panel.

**Session Objectives:** Participants will be able to:

1. Recognize the signs and symptoms of an iliopsoas strain in dogs;
2. Understand how to identify caudal cervical spondylomyelopathy in horses;
3. Discern the signs and functional therapeutic needs for dogs with hip osteoarthritis; and
4. Understand how human physiotherapy literature can be utilized to guide assessment and treatments in animal rehab.

**Keywords:** animal rehab, iliopsoas, canine hip dysplasia, equine caudal cervical spondylomyelopathy

# Abstract: D010 – INTER-PROFESSIONAL COLLABORATION IN ANIMAL REHAB CLINICAL PRACTICE – VETERINARY AND PHYSIOTHERAPY PERSPECTIVES

**Published:** 2012-05

**Authors:** LM Edge-Hughes, S Budiselic

**Affiliations:** The Canine Fitness Centre Ltd., Calgary, Alta

**Background:** The two co-presenters (a physiotherapist and a veterinarian) have worked together collaboratively in a canine rehabilitation practice for over 2 years. Additionally, they have knowledge of and insight to collaborative practice models in animal rehab through colleagues, associates, and general observations from each of their professional perspectives. Inter-professional collaborative practice is in its infancy in veterinary medicine. Physiotherapists have a long history of working in multi-disciplinary teams in human health care and have much to offer the veterinary profession as partners in animal health care. The fundamental differences between physiotherapists and veterinarians in their approach to assessment and treatment are a result of the education and philosophies that underpinning each profession. It is useful to comprehend the differences between the veterinary and physiotherapy professions to better understand how collaboration might be possible in the field of animal rehab.

**Relevance to Physiotherapy Practice:** Human medicine has incorporated many health care professionals, whose collective goals are directed towards patient-centered care. Veterinary medicine in Canada however, does not yet formally utilize allied health care professionals. Physical therapists are the first professionals to approach the veterinary field with the full intent to work collaboratively within a veterinary framework and set a precedent regarding interdisciplinary health practice. Working with veterinarians and utilizing physiotherapy scientific knowledge and skills on animal patients is an evolution of the physiotherapy profession, which simultaneously promotes growth in the veterinary profession.

**Target Audience:** This session will be of interest to physiotherapists engaged in animal rehab, those with an interest or curiosity in the field, and policy decision-makers in both the physiotherapy and veterinary professions.

**Summary of Supporting Evidence:** Literature can be found in ’human’ medical references of scientific, theoretic and anecdotal nature, that promote multidisciplinary collaboration in a patient-centric model of care. Qualitative research further elucidates how inter-professional relationships are perceived, developed, and fostered by different professional groups. (Baxter & Brufitt 2008; Malcolm & Scott 2011) However, there is a complete void in veterinary literature on the topic of intra-professional collaboration. Surveys of the veterinary profession’s attitudes towards ’animal physiotherapy’ have been conducted, which may help to shed light on potential barriers as well as opportunities for collaborative practice. (Doyle & Horgan 2006)

The physiotherapy profession has long historically recognized the importance and legitimacy of a physical therapy/clinical diagnosis prior to treatment of a patient. (Sarhmann 1988) A patho-functional diagnosis by a physiotherapist evaluates physical function among other things, whereas a medical/patho-anatomic diagnosis serves only to identify the injured structure and does not, by nature, take into account the function of the patient. Differences in diagnostic approaches and conclusions between the physiotherapy profession and the veterinary profession are best highlighted by the fact that there is a complete lack of any one comprehensive text book or manual on veterinary orthopaedic assessment (outside of surgical texts – which expound largely on surgical reduction of orthopaedic pathologies).

**Description of Session Format:** This session will be a one-hour lecture format, with case examples to assimilate the information presented with time allotted for questions & answers.

**Session Objectives:** At the end of this session, participant will be able to:

1. Understand the barriers and opportunities for collaborative practice in animal rehabilitation;
2. Comprehend the fundamental differences between a physiotherapy assessment and a traditional veterinary assessment of an animal patient;
3. Identify knowledge gaps and areas of interventional need in veterinary medicine that can be fulfilled by physiotherapists and the science of physical rehabilitation.

**Keywords:** animal rehabilitation, inter-professional collaboration, multidisciplinary, physiotherapists, veterinarians

# Abstract: D011 – REHABILITATION FOLLOWING CRITICAL ILLNESS

**Published:** 2012-05

**Authors:** S Berney

**Affiliations:** Physiotherapy Department Austin Health, Victoria, Australia

**Background:** Post-intensive care syndrome has been identified as a significant problem. Survivors of critical illness may have long standing weakness, as well as, cognitive and mental health impairment. Consensus conferences in both the US and Europe have been devoted to describing the problem and developing research agendas to address the issues involved. One of the major concerns identified in the US consensus conference was that the broader medical community appears not to appreciate the multiple impairments associated with surviving an intensive care unit admission. It is essential as physiotherapists we understand the extent of the pathological processes that affect patients so that we are able to provide not only best treatment but can also be strong advocates for our patients.

**Relevance to Physiotherapy Practice:** The legacy of surviving an intensive care admission can include prolonged weakness and impaired neuro-cognitive and mental health function. This one hour session is intended to increase clinician’s understanding of the full spectrum of impairments associated with surviving critical illness. It is essential that physiotherapists rehabilitating people following critical illness understand the full extent of the pathological processes in order to provide comprehensive treatment. This session will also help clinicians identify effective and evidence-informed treatment strategies for the rehabilitation of survivors of critical illness across the spectrum of care from intensive care to community rehabilitation.

**Target Audience:** Physiotherapists working in the acute care setting, rehabilitation and community.

**Summary of Supporting Evidence:** The progress of intensive care medicine has resulted in improvements in rates of survival. However survivors of critical illness can be left with a legacy of prolonged weakness; reduced physical capacity and impaired neuro- cognitive and mental function that impacts their health related quality of life for up to five years following ICU discharge (Desai et al., 2011; Cuthbertson et al., 2010). This constellation of symptoms is called the post-intensive care syndrome (Needham et al., 2012). To date the causes of these impairments are poorly understood. These reports of sustained impairments in physical, cognitive and mental function have resulted in critical care clinicians redefining the research agenda to include patient-centred outcomes in addition to mortality (Vincent et al., 2009). There has been increased interest in the identification of the potential harmful effects of various treatments delivered in the ICU and the provision of interventions to prevent or ameliorate these symptoms (Desai et al, 2011; Needham et al., 2012).

One such intervention that has been described for the prevention and treatment of muscle weakness is rehabilitation (that includes early mobilization), commenced in the ICU (Desai et al., 2011). There is low level evidence to support the role of early mobilization in reducing the ICU length of stay and improving longer term outcomes such as readmission to hospital (Morris et al., 2008; Morris et al., 2011). In addition there is high level evidence that rehabilitation commenced in the ICU can improve functional capacity at hospital discharge (Burtin et al., 2009; Schweikert et al., 2009). The results of our own randomized controlled trial, where rehabilitation was continued from ICU onto the acute ward and through to an 8 week outpatient programme, demonstrated improved physical function at 12 months following ICU discharge. Current research is focused on developing diagnostic and outcome measures, identifying the contributing mechanisms that lead to long term sequelae of an intensive care admission, establishing the efficacy of interventions commenced early in an ICU admission and assessing the effects of rehabilitation commenced in the ICU and continued into the community on longer term patient centred outcomes.

**Description of Session Format:** Lecture

**Session Objectives:**

1. To develop an understanding of the physical, cognitive and mental health impairments associated with post-intensive care syndrome
2. To develop an understanding of the evidence regarding the role of rehabilitation for survivors of critical illness
3. To identify the outcome measures that have been used to establish the efficacy of rehabilitation programs for survivors of critical illness
4. To identify treatment strategies that can be implemented in clinical practice for the rehabilitation of survivors of critical illness across the continuum of care

**Keywords:** rehabilitation, critical illness

# Abstract: D012 – ADOLESCENTS WITH CANCER: LIVES INTERRUPTED

**Published:** 2012-05

**Authors:** A Rankin, M Juricic, E Peddie, A Johnson

**Affiliations:** BC Children’s Hospital Oncology Hematology and Bone Marrow Transplant Program, Vancouver, B.C.

**Background:** The adolescent diagnosed with cancer has a multitude of medical and psychosocial challenges. Research that has been completed is usually based on small populations and may be biased to those adolescents predisposed to physical activity or have more severe late effects. Late effects of treatment can have profound effects on quality of life, performance and participation. Facilities may not be able to physically accommodate the specific needs of this patient group. Exercise and the short and long term implications of physical activity will be discussed as well as barriers to exercise and programs that promote exercise adherence. A program at BC Children’s Hospital designed to meet the psychosocial needs of adolescents undergoing treatment will be highlighted.

**Relevance to Physiotherapy Practice:** Adherence to physical activity programs requires sensitivity to the developmental needs of the adolescent. With basic understanding of barriers to physical activity and methods to promote adherence, physical therapists may improve patient outcomes by reducing fatigue and improving strength, flexibility and cardiovascular endurance.

**Target Audience:** The target audience should have experience treating adolescent patients and a basic understanding of exercise testing.

**Summary of Supporting Evidence:** Prospective studies and surveys of patients have indicated that adolescents and adolescent survivors of childhood cancer have reduced strength; higher body mass index; lower bone density; reduced cardiovascular fitness and flexibility; and ongoing complaints of fatigue that interfere with quality of life (Berg et al., 2009; Brussel et al., 2005; Ness and Gurney, 2007; Jarvela et al., 2010). Many studies are based upon limited numbers and may include a bias towards selection of patients with more severe complications/late effects or more physically fit, depending on the targeted group of the study (Arroyave et al., 2008). Psychosocial needs of the adolescent are well identified (King et al., 2008) and need to be considered when designing programs for this patient population.

**Description of Session Format:** This session will be a lecture format, with opportunity for participant discussion regarding their facility’s solutions for challenges faced by this specific patient group.

**Session Objectives:** Upon completion of this session, participants will be able to:

1. Discuss the medical and psychosocial challenges facing the adolescent with cancer;
2. Define late effects of treatment versus complications of treatment;
3. Provide examples of late effects that occur in pediatric cancer survivors; and
4. Discuss alternate programs to promote exercise adherence and remove barriers to physical activity

**Keywords:** adolescent, cancer, survivor, psychosocial, medical, exercise

## References

[B5] Arroyave W, Clipp E, Miller P, Jones L, Ward D, Bonner M, Rosoff P, Snyder D, Demark-Wahnefried W (2008). Childhood cancer survivors’ perceived barriers to improving exercise and dietary behaviors. Oncology Nursing Forum.

[B6] Berg C, Neufeld P, Harvey J, Downes A, Hayashi R (2009). Late effects of childhood cancer, participation, and quality of life of adolescents. Occupational Therapy JR.

[B7] Brussel M, Takken T, Lucia A, Net J, Helders P (2005). Is physical fitness decreased in survivors of childhood leukemia? A systematic review. Leukemia.

[B8] Jarvela L, Niinikoski H, Lahteenmaki P, Heinonen O, Kapanen J, Arola M, Kemppainen J (2010). Physical activity and fitness in adolescent and young adult long-term survivors of childhood acute lymphoblastic leukaemia. Journal of Cancer Survivor.

[B9] King K, Tergerson J, Wilson B (2008). Effect of social support on adolescents’ perceptions of and engagement in physical activity. Journal of Physical Activity and Health.

[B10] Ness K, Gurney J (2007). Adverse late effects of childhood cancer and its treatment on health and performance. The Annual Review of Public Health.

[B11] White J, Flohr J, Winter S, Vener J, Feinauer L, Ransdell L (2005). Potential benefits of physical activity for children with acute lymphoblastic leukaemia. Pediatric Rehabilitation.

# Abstract: D013 – BRAIN MECHANISMS OF THE COGNITIVE, SENSORY AND EMOTIONAL SIDES OF CHRONIC PAIN

**Published:** 2012-05

**Authors:** DA Seminowicz

**Affiliations:** University of Maryland, Pain Imaging Lab, Baltimore Maryland, USA

**Background:** While it is largely accepted that chronic pain is a disease of the brain, our understanding of the role of specific brain regions and networks in pain processing is still very limited. Brain imaging studies have changed what we know about the brain’s role in acute and chronic pain, and are providing hope for patients by identifying novel targets for pain relief.Dr. Seminowicz will present recent findings from his research program out of the Pain Imaging Lab at the University of Maryland. The presentation will focus on measurable morphologic changes in the brain of people with chronic pain and how such findings might impact rehabilitation.

**Relevance to Physiotherapy Practice:** Pain is a multidimensional experience. The lecture will provide evidence that distinct aspects of the experience can affect the brain differentially. A good understanding of how pain and treatment affect brain function and quality of life will lead to better use of multidisciplinary pain medicine. Physiotherapists will gain an appreciation of brain plasticity and how such changes manifest in the clinical presentation and management of people with complex chronic pain.

**Target Audience:** The lecture will be accessible to researchers and clinicians with a basic understanding of research methods and the clinical issues related to chronic pain.

**Summary of Supporting Evidence:** The evidence discussed will be basic science studies in humans and animals, much of which will be Dr. Seminowicz’s own work, both published and yet to be published. Most of the literature discussed will be on brain neuroimaging in pain, of which there are currently several hundred studies published on various topics, primarily case-control and cohort studies. A sampling of Dr. Seminowicz’s peer-reviewed publications can be found here: http//www.ncbi.nlm.nih.gov/pubmed?term=seminowicz

**Description of Session Format:** This 1 hour session will be lecture style with opportunity for questions and interaction provided at the end.

**Session Objectives:** Upon completion of this session, participants will be able to describe:

1. how distinct brain areas and networks are related to the different dimensions of the pain experience.
2. current research in the field of brain imaging that is allowing us to better understand the role of the brain in pain.
3. how chronic pain affects the brain, and how treating pain might be able to reverse altered brain structure and function.

**Keywords:** magnetic resonance imaging, plasticity, brain, pain
